# The Balance of Stromal BMP Signaling Mediated by *GREM1* and *ISLR* Drives Colorectal Carcinogenesis

**DOI:** 10.1053/j.gastro.2020.11.011

**Published:** 2020-11-14

**Authors:** Hiroki Kobayashi, Krystyna A. Gieniec, Josephine A. Wright, Tongtong Wang, Naoya Asai, Yasuyuki Mizutani, Tadashi Lida, Ryota Ando, Nobumi Suzuki, Tamsin R. M. Lannagan, Jia Q. Ng, Akitoshi Hara, Yukihiro Shiraki, Shinji Mii, Mari Ichinose, Laura Vrbanac, Matthew J. Lawrence, Tarik Sammour, Kay Uehara, Gareth Davies, Leszek Lisowski, Ian E. Alexander, Yoku Hayakawa, Lisa M. Butler, Andrew C. W. Zannettino, M. Omar Din, Jeff Hasty, Alastair D. Burt, Simon J. Leedham, Anil K. Rustgi, Siddhartha Mukherjee, Timothy C. Wang, Atsushi Enomoto, Masahide Takahashi, Daniel L. Worthley, Susan L. Woods

**Affiliations:** 1 Adelaide Medical School, University of Adelaide, Adelaide, Australia; 2 South Australian Health and Medical Research Institute, Adelaide, Australia; 3 Department of Pathology, Nagoya University Graduate School of Medicine, Nagoya, Japan; 4 Division of Molecular Pathology, Center for Neurological Disease and Cancer, Nagoya University Graduate School of Medicine, Nagoya, Japan; 5 Department of Molecular Pathology, Graduate School of Medicine, Fujita Health University, Toyoake, Japan; 6 Department of Gastroenterology and Hepatology, Nagoya University Graduate School of Medicine, Nagoya, Japan; 7 Department of Gastroenterology, Graduate School of Medicine, The University of Tokyo, Tokyo, Japan; 8 Department of Cardiology, Nagoya University Graduate School of Medicine, Nagoya, Japan; 9 Colorectal Unit, Department of Surgery, Royal Adelaide Hospital, Adelaide, Australia; 10 Division of Surgical Oncology, Department of Surgery, Nagoya University Graduate School of Medicine, Nagoya, Japan; 11 UCB Pharma, Slough, Berkshire, United Kingdom; 12 Translational Vectorology Research Unit, Children’s Medical Research Institute, Faculty of Medicine and Health, The University of Sydney, Sydney, Australia; 13 Vector and Genome Engineering Facility, Children’s Medical Research Institute, Faculty of Medicine and Health, The University of Sydney, Westmead, Australia; 14 Military Institute of Hygiene and Epidemiology, The Biological Threats Identification and Countermeasure Centre, Puławy, Poland; 15 Gene Therapy Research Unit, Sydney Children’s Hospitals Network and Children’s Medical Research Institute, Faculty of Medicine and Health, The University of Sydney, Sydney, Australia; 16 Discipline of Child and Adolescent Health, Faculty of Medicine and Health, The University of Sydney, Australia; 17 GenCirq, Inc, San Diego, California; 18 Department of Bioengineering, University of California, San Diego, La Jolla, California; 19 Precision and Molecular Pathology, Newcastle University, Newcastle Upon Tyne, United Kingdom; 20 Intestinal Stem Cell Biology Lab, Wellcome Trust Centre Human Genetics, University of Oxford, Oxford, United Kingdom; 21 Herbert Irving Comprehensive Cancer Center, Division of Digestive and Liver Diseases, Department of Medicine, Columbia University, New York, New York; 22 Department of Medicine and Irving Cancer Research Center, Columbia University, New York, New York; 23 International Center for Cell and Gene Therapy, Fujita Health University, Toyoake, Japan

**Keywords:** Colorectal Cancer, Cancer-Associated Fibroblasts, Tumor Microenvironment, Bone Morphogenetic Protein

## Abstract

**Background & AIMS:**

Cancer-associated fibroblasts (CAFs), key constituents of the tumor microenvironment, either promote or restrain tumor growth. Attempts to therapeutically target CAFs have been hampered by our incomplete understanding of these functionally heterogeneous cells. Key growth factors in the intestinal epithelial niche, bone morphogenetic proteins (BMPs), also play a critical role in colorectal cancer (CRC) progression. However, the crucial proteins regulating stromal BMP balance and the potential application of BMP signaling to manage CRC remain largely unexplored.

**Methods:**

Using human CRC RNA expression data, we identified CAF-specific factors involved in BMP signaling, then verified and characterized their expression in the CRC stroma by in situ hybridization. CRC tumoroids and a mouse model of CRC hepatic metastasis were used to test approaches to modify BMP signaling and treat CRC.

**Results:**

We identified *Grem1* and *Islr* as CAF-specific genes involved in BMP signaling. Functionally, GREM1 and ISLR acted to inhibit and promote BMP signaling, respectively. *Grem1* and *Islr* marked distinct fibroblast subpopulations and were differentially regulated by transforming growth factor *β* and FOXL1, providing an underlying mechanism to explain fibroblast biological dichotomy. In patients with CRC, high *GREM1* and *ISLR* expression levels were associated with poor and favorable survival, respectively. A GREM1-neutralizing antibody or fibroblast *Islr* overexpression reduced CRC tumoroid growth and promoted *Lgr5^+^* intestinal stem cell differentiation. Finally, adeno-associated virus 8 (AAV8)–mediated delivery of *Islr* to hepatocytes increased BMP signaling and improved survival in our mouse model of hepatic metastasis.

**Conclusions:**

Stromal BMP signaling predicts and modifies CRC progression and survival, and it can be therapeutically targeted by novel AAV-directed gene delivery to the liver.

## Graphical abstract

**Figure:**
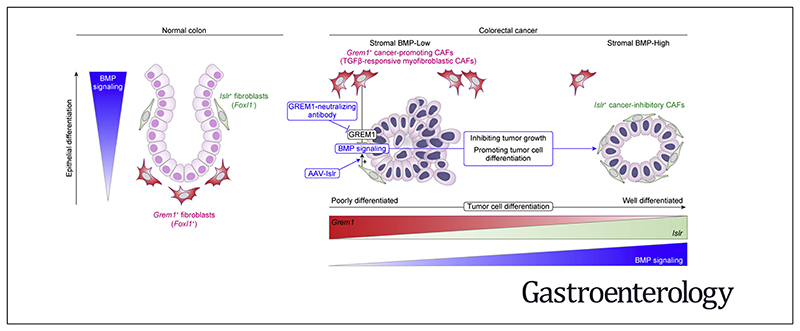

Colorectal cancer (CRC) is a major cause of cancer mortality.^1^ Despite advances in surgical techniques and medical therapies targeting tumor cells, endothelial cells, and immune cells, the majority of patients with metastatic CRC still die of their disease.^1^

Cancer-associated fibroblasts (CAFs), a key constituent of the tumor microenvironment, influence CRC initiation, progression, and dissemination and can promote drug resistance via secretion of growth factors, chemokines, extracellular matrix, and proangiogenic factors.^2^ CAFs are not a uniformly protumorigenic entity; rather, CAFs are composed of functionally heterogeneous subpopulations, including tumorpromoting CAFs and tumor-retarding CAFs.^2^ However, the markers and mechanisms underlying this CAF biological dichotomy are largely unknown, which has hampered therapeutic attempts to exploit these differences.^2^

One key family of growth factors secreted by CAFs, as well as cancer cells, are the bone morphogenetic proteins (BMPs).^3^ BMPs belong to the transforming growth factor *β*) (TGF-*β*) superfamily. Binding of BMP ligands, such as BMP2,4, and 7, to type I and II BMP receptors induces phosphorylation of SMAD1/5/8, which in turn binds SMAD4 to increase target gene expression such as *ID1,2,3*, and *4*.^3^ BMP gradients partly define the intestinal epithelial stem cell niche in the normal colon and serve to promote or retard cancer progression in a context-dependent manner.^3^ In the normal colon, the epithelial stem cell niche is maintained by low BMP and high Wnt at the crypt base, whereas epithelial cell differentiation is driven by increasing BMP and low Wnt toward the luminal surface.^4^ The BMP gradient is finely tuned by BMP inhibitors, such as GREM1 and NOGGIN, which are secreted by fibroblasts near the crypt base.^4–6^ In CRC, inactivation of BMP signaling through germline or sporadic mutations in BMP receptors and *SMAD4* contributes to CRC predisposition and progression.^4^ Numerous studies have shown the tumor-retarding role of BMP signaling in CRC cells themselves.^3,7,8^ BMP signaling has been shown to reduce stemness of intestinal stem cells such as *Lgr5*^+^ intestinal stem cells, leading to epithelial differentiation.^7,8^ Most CRC studies, however, have failed to address the function of stromal BMP signaling in CRC progression.

Here, we identified gremlin 1 (*GREM1*) and immunoglobulin superfamily containing leucine-rich repeat (*ISLR*), specifically and distinctly expressed by different types of CRC CAFs, as important regulators of BMP signaling within the tumor microenvironment.

*GREM1*, a ligand-sequestering antagonist for BMP2, 4, and 7, is expressed by mesenchymal stem/stromal cells in the bone marrow and intestinal fibroblasts.^6^ *GREM1* expressed by CAFs can accelerate tumor cell proliferation via inhibition of BMP signaling.^9^ In patients with CRC or breast cancer, high *GREM1* expression is associated with poor prognosis.^10–12^ GREM1 secreted by glioma cancer stem cells blocks BMP2-induced differentiation of glioma cells, thereby maintaining their proliferation and stemness.^13^ Furthermore, overexpression of *GREM1* in intestinal epithelial cells initiates colonic tumorigenesis, supporting the protumorigenic role of *GREM1*.^14,15^

ISLR (also known as Meflin), a glycosylphosphatidylinositol-anchored membrane protein, which is also secreted, was recently identified as a specific marker for mesenchymal stem/stromal cells and fibroblasts in various organs including the bone marrow, heart, and pancreas.^16,17^ In contrast to the tumor-promoting role of *GREM1*, our recent study found that *ISLR* defines a subset of tumor-retarding CAFs that are distinct from a-smooth muscle actin (*α*SMA)^+^ CAFs in pancreatic cancer.^16^ ISLR interacts with BMP7 to augment BMP7-Smad1/5 signaling.^17^ Although a recent report has indicated that *ISLR* is highly expressed by fibroblasts in the inflamed colon and CRC,^18^ the biological role of ISLR in CRC related to BMP signaling remains unknown.

In this study, after the identification and validation of *GREM1* and *ISLR* as 2 functionally opposing BMP-related genes specifically expressed by CRC CAFs, we examined the prognostic significance of *GREM1* and *ISLR* expression levels in human CRC. Then, we characterized distinct expression patterns of *GREM1* and *ISLR* in both normal colon and CRC and the potential mechanism by which this CAF polarization occurs. Next, we explored whether a GREM1-neutralizing antibody or conditioned medium transfer from *Islr*-overexpressing colonic fibroblasts could restrain CRC organoid growth. Finally, we investigated whether adeno-associated virus (AAV) 8-mediated ectopic overexpression of *Islr* in hepatocytes could retard CRC liver metastasis progression in mice as a novel therapeutic approach to restrain CRC metastasis progression.

## Materials and Methods

### Statistical Analysis

A comparison of 2 groups was performed by using 2-tailed unpaired *t* tests or Mann-Whitney *U* tests. For multiple comparisons, we used analysis of variance (ANOVA) with subsequent Tukey or Sidak post hoc analysis (for parametric tests) or Kruskal-Wallis test followed by Dunn post-hoc multiple comparisons (for nonparametric tests). For survival analyses, Kaplan-Meier survival estimation with a log rank (Mantel-Cox) test was performed. Statistical analyses were conducted using GraphPad Prism 8.00 (GraphPad) or SPSS Statistics, version 25 (IBM). *P* values of less than .05 were considered statistically significant.

For all other Materials and Methods, see the Supplementary Materials.

## Results

### Identification of Cancer-Associated Fibroblast–Specific Expression of the Bone Morphogenetic Protein Antagonist GREM1 and the Bone Morphogenetic Protein Potentiator ISLR in Colorectal Cancer

**Figure 1:**
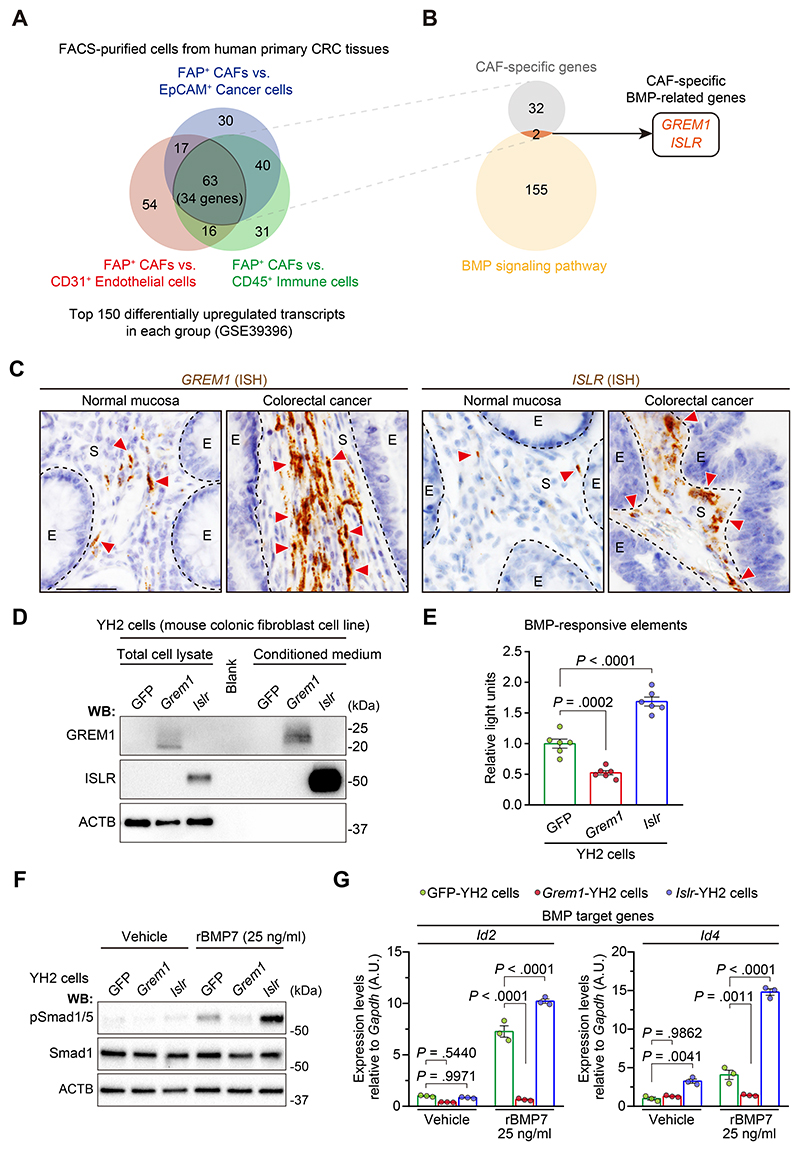
Identification of *GREM1* and *ISLR* as a BMP antagonist and potentiator, respectively, specifically expressed by CRC CAFs. (*A*, *B*) Analysis of expression microarray data from FACS-purified cells from human primary CRC tissues. (*A*) Venn diagram depicting the overlap of the top 150 differentially up-regulated transcripts in the 3 groups as indicated. (B) Venn diagram showing the overlap of 34 CAF-specific genes and 157 BMP-related genes identified by GO of the BMP signaling pathway (GO: 0030509) and Hara et al.^17^ (C) ISH for *GREM1* and *ISLR* in the human normal colorectal mucosa and CRC. Dotted lines indicate the borders between epithelial cells (E) and the stroma (S). Red arrowheads denote *GREM1* or *ISLR* expression. Scale bar, 50 *μ*m. (D–G) Lentivirus-mediated overexpression of *Grem1* and *Islr* in a mouse colonic fibroblast cell line, YH2 cells, represses and augments BMP signaling, respectively. (D) Western blotting (WB) showing *Grem1* and *Islr* overexpression in the total cell lysates and CM. (E) Luciferase assays of BMP-responsive elements; n = 6. (F, G) YH2 cells were stimulated with recombinant BMP7, followed by (F) WB and (G) qRT-PCR; n = 3. Mean ± standard error of the mean (SEM). One-way ANOVA *(E)* or 2-way ANOVA (G) with post hoc Tukey multiple comparisons. A.U., arbitrary unit.

To identify which BMP-related genes are specifically expressed by CAFs, we first analyzed expression microarray data from a study of fluorescence-activated cell sorting (FACS)–purified cells from human primary CRC tissues.^19^ The top 150 differentially expressed gene probes up-regulated in CAFs in each group (FAP^+^ CAFs vs EpCAM^+^ cancer cells, FAP^+^ CAFs vs CD31^+^ endothelial cells, and FAP^+^ CAFs vs CD45^+^ immune cells) were selected for our analysis, resulting in the identification of 34 genes specifically expressed in human CRC CAFs (Figure 1*A*, Supplementary Figure 1*A* and *B*, and Supplementary Table 1). Next, to examine for genes involved in BMP signaling, we compared the 34 CAF-specific genes with BMP-relevant genes listed in Gene Ontology (GO) and identified by literature review (BMP signaling pathway; GO0030509)^17^ (Figure 1*B*). This analysis identified 2 genes, *GREM1* and *ISLR*, as human CRC CAF-specific genes relevant to BMP signaling.

To validate *GREM1* and *ISLR* expression in human CRC CAFs, we performed RNA in situ hybridization (ISH) on CRC patient samples and confirmed that both *GREM1* and *ISLR* are highly expressed by fibroblastic cells in the human CRC stroma compared to the normal colorectal stroma (Figure 1*C* and Supplementary Figure 2). Consistent with the microarray data, single-molecule fluorescent ISH (smFISH) for *GREM1* and *ISLR* followed by FAP immunofluorescence showed that *GREM1* and *ISLR* were expressed by FAP^+^ CAFs in human CRC sections (Supplementary Figure 3). Moreover, the fibroblast-specific expression pattern of *GREM1* and *ISLR* was corroborated by analyses of publicly available single-cell RNA sequencing (scRNA-seq) data from human normal colon mucosa and primary CRC^20^ (Supplementary Figure 4*A* and *B*).

We next sought to verify the functional roles of GREM1 and ISLR in the regulation of BMP signaling, using lentivirus-mediated overexpression of *Grem1* or *Islr* in a mouse colonic fibroblast cell line, YH2 cells. GREM1 and ISLR overexpression was detected in the conditioned medium from *Grem1*-overexpressing and Islr-overexpressing YH2 cells, respectively, suggesting that GREM1 and ISLR were secreted into the medium (Figure 1D). Luciferase assays of BMP-responsive elements showed that GREM1 overexpression suppressed, whereas ISLR overexpression augmented, BMP signaling (Figure 1E). Furthermore, GREM1 overexpression inhibited BMP7-mediated phosphorylation of Smad1/5, a downstream effector of BMP signaling, thereby preventing the BMP7-induced increase in the expression of BMP target genes *Id2* and *Id4* (Figure 1F and G). Conversely, these surrogates for the BMP signaling pathway were increased by ISLR overexpression. When Grem1-overexpressing YH2 cells were admixed with Islr-overexpressing YH2 cells, GREM1 and ISLR counteracted each other’s effect on BMP7 signaling (Supplementary Figure 5). Similar to the GREM1 antagonism of BMP7 signaling, GREM1 overexpression also prevented the BMP2-induced increase in *Id2* and Id4. In contrast, ISLR overexpression promoted the BMP2-mediated increase in Id4, but not Id2 (Supplementary Figure 6). Collectively, these data indicate that 2 functionally opposing stromal regulators of BMP signaling, *GREM1* and *ISLR*, are up-regulated in CRC CAFs and may contribute to fine-tuning of BMP signaling within the CRC stroma.

### *GREM1* and *ISLR* Expression Are Up-regulated During Colorectal Carcinogenesis in Humans

**Figure 2:**
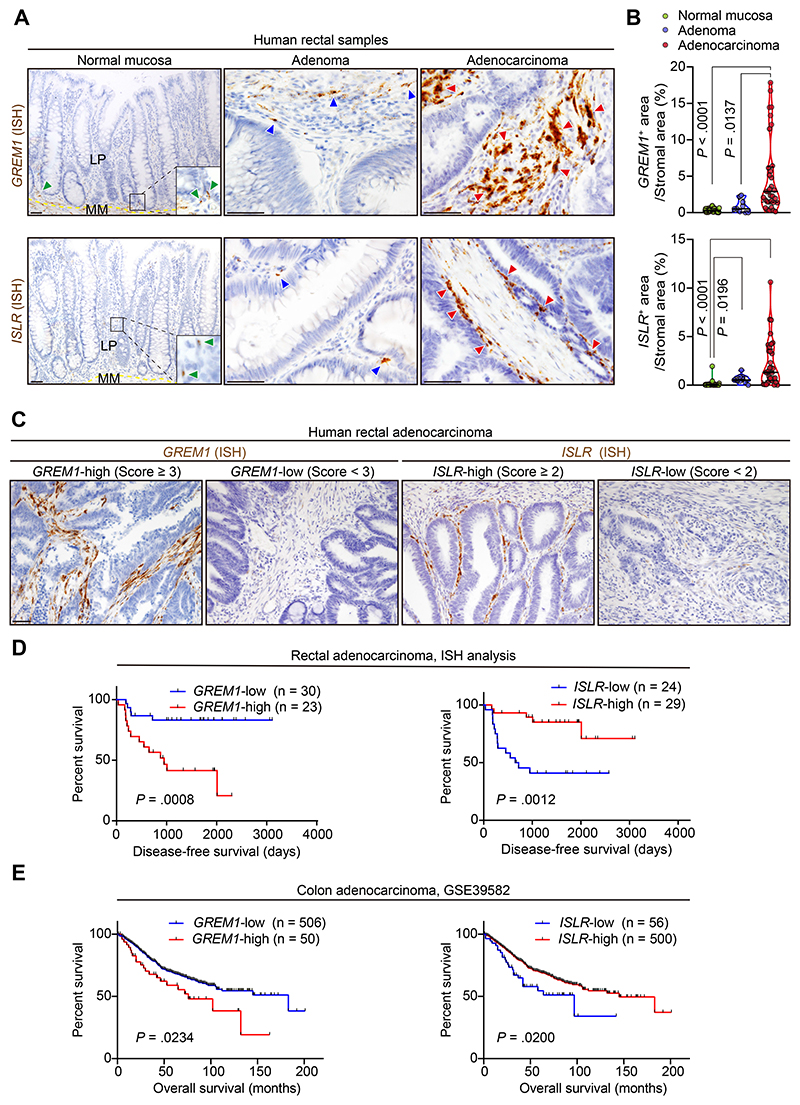
*GREM1* and *ISLR* expression levels are associated with poor and favorable prognosis in patients with CRC, respectively. (*A*, *B*) ISH for *GREM1* and *ISLR* using human rectal samples. (*A*) Representative images. Yellow dotted lines indicate the borders between the lamina propria (LP) and muscularis mucosa (MM). Green, blue, and red arrowheads denote *GREM1* or *ISLR* expression in the normal mucosa, adenoma, and adenocarcinoma, respectively. (*B*) Violin plots depicting *GREM1* and *ISLR* ISH signal^+^ areas in the stroma. Three high-power fields (400×) per patient: 11 patients with normal mucosa, 3 with adenoma, and 11 with adenocarcinoma. Solid black lines indicate the median; dotted black lines indicate quartiles. (*C*, *D*) ISH analysis of 53 human primary rectal cancer surgical samples. (*C*) Representative images. Cases with a score of ≥3 and a score of ≥2 were defined as *GREM1*-high and ISLR-high, respectively. (*D*) Kaplan-Meier survival curves. (*E*) Kaplan-Meier survival curves in expression microarray data from 556 patients with primary colon cancer. Kruskal-Wallis test followed by Dunn post hoc multiple comparisons (*B*) and log rank test (*D* and *E*). Scale bars, 50 *μ*m.

Next, we investigated whether expression of GREM1 and *ISLR* is up-regulated during CRC progression. ISH for *GREM1* and *ISLR*, as well as scRNA-seq and expression microarray analyses, showed that *GREM1* and *ISLR* expression were increased during human colorectal carcinogenesis (Figure 2A and B and Supplementary Figures 4A and 7A–C). *GREM1* and *ISLR* up-regulation was also observed in the stroma of liver metastases of human CRC compared to the normal liver tissues (Supplementary Figure 8A–C). Furthermore, in line with the fibroblast-specific expression of *GREM1* and *ISLR*, analyses of The Cancer Genome Atlas and expression microarray data showed that the highest expression levels of *GREM1* and *ISLR* were observed in a stroma-rich molecular subtype of CRC (consensus molecular subtype 4 [CMS4]) (Supplementary Figure 9A and B). This is consistent with a recent article showing that CMS4 tumors displayed the highest *GREM1* transcript levels.^10^ Overall, these data suggest that *GREM1* and *ISLR* expression are up-regulated in the CRC stroma during colorectal carcinogenesis.

### *GREM1* and *ISLR* Expression Levels Are Associated With Poor and Favorable Clinical Outcomes in Patients With Colorectal Cancer, Respectively

To investigate the clinical significance of *GREM1* and *ISLR* expression in CRC CAFs, we evaluated *GREM1* and *ISLR* expression by ISH in 53 rectal cancer surgical samples (Figure 2C and Supplementary Table 2). Survival analyses showed that high *GREM1* expression (score of ≥3) and high *ISLR* expression (score of ≥2) were independent prognostic factors for poor and favorable disease-free survival, respectively, in patients with rectal cancer (Figure 2D and Supplementary Tables 2 and 3).

Furthermore, analysis of expression microarray data from 556 patients with primary colon cancer confirmed that the *GREM1*-high and *ISLR*-low groups each independently exhibited poor overall survival (Figure 2E and Supplementary Table 4). No patients in this cohort had both *GREM1*-high and *ISLR*-low, suggesting that *GREM1*-high patients and *ISLR*-low patients were 2 separate patient subgroups (Supplementary Table 5). Together, these data indicate that *GREM1* and *ISLR* expression levels may serve as prognostic biomarkers in human CRC, with *GREM1* expression associated with poorer and *ISLR* expression associated with improved survival.

### *Grem1*^+^ Fibroblasts Are Distinct From *Islr*^+^ Fibroblasts in the Normal Mouse Colon, With the Majority of *Grem1*^+^ Fibroblasts Marked by *Foxl1*

**Figure 3:**
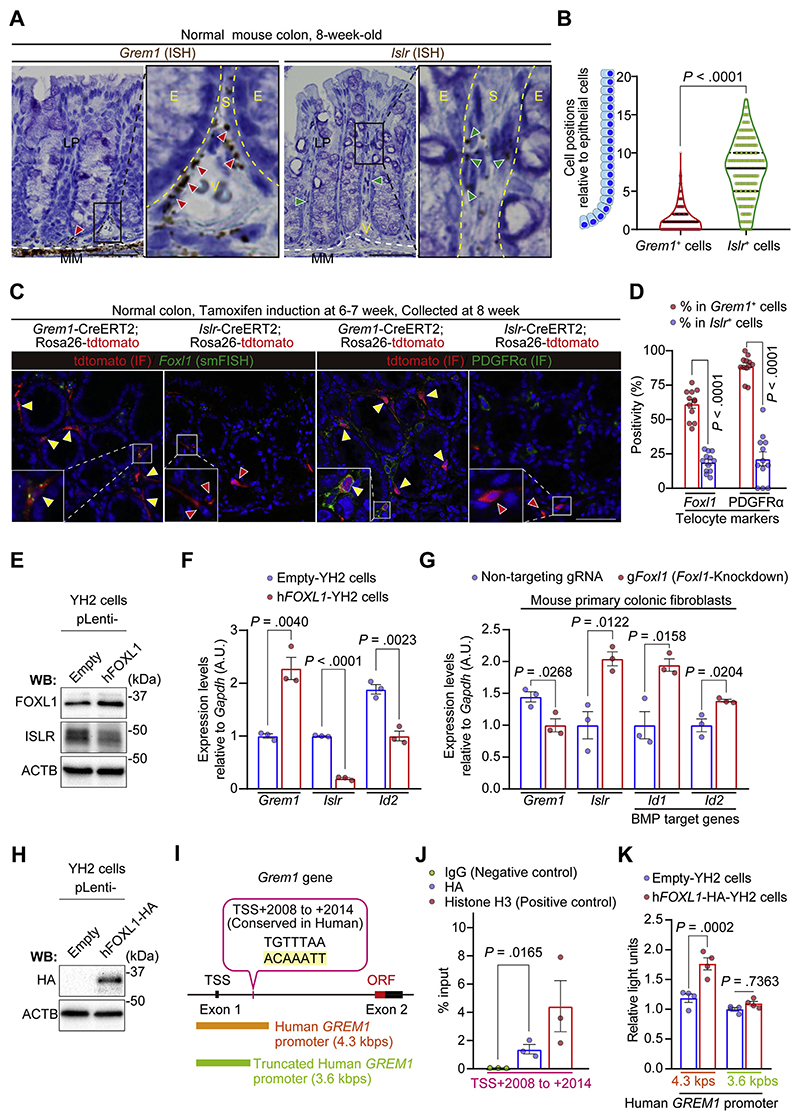
*Grem1* and *Islr* identify distinct subpopulations of intestinal fibroblasts in the normal mouse colon and are differentially regulated by FOXL1. (*A*, *B*) ISH for *Grem1* and *Islr* in the adult normal mouse colon. (*A*) Representative images. Red and green arrowheads denote *Grem1*^+^ cells and *Islr*^+^ cells, respectively. Yellow dotted lines delineate the boundaries between epithelial cells (E) and stromal cells (S). White dotted lines indicate the borders between the lamina propria (LP) and muscularis mucosa (MM). V indicates blood vessels. (*B*) Violin plots depicting the positions of mesenchymal cells expressing *Grem1* or *Islr* relative to the adjacent epithelial position; 344 *Grem1*^+^ cells and 512 *Islr*^+^ cells from 20 well-oriented crypts/mouse, 4 mice each. Black solid lines indicate the median; black dotted lines indicate quartiles. (*C*, *D*) smFISH for *Foxl1* and IF for PDGFR*α* in Grem1-CreERT2 mice and Islr-CreERT2 mice. (*C*) Representative pictures. Yellow arrowheads indicate double-positive cells (*Grem1*^+^*Foxl1*^+^ cells or Grem1^+^PDGFR*α*^+^ cells). Red arrowheads denote *Islr* single-positive cells. (*D*) *Foxl1* positivity and PDGFR*α* positivity in the *Grem1*^+^ cells and *Islr*^+^ cells. Four high-power fields (400×)/mouse, 3 mice each. (*E*, *F*) Lentivirus-mediated human *FOXL1 (hFOXL1)* overexpression in YH2 cells induces *Grem1* up-regulation and decreases *Islr* expression. (*E*) Western blot. (*F*) qRT-PCR (n = 3). (*G*) CRISPR/Cas9-mediated knockdown of *Foxl1* reduces *Grem1* expression while upregulating *Islr* expression in primary mouse colonic fibroblasts as assessed by qRT-PCR (n = 3 mice each). (*H*–*K*) FOXL1 interacts with a *Grem1* intron region. (*H*) Western blot showing hFOXL1-HA overexpression in YH2 cells. (*I*) Schematic representation of a FOXL1-binding site in the mouse *Grem1* intron (*highlighted with yellow*) and corresponding human *GREM1* promoter regions used in luciferase assays. (*J*) ChIP–quantitative PCR in YH2 cells (n = 3). (*K*) Luciferase assays of a human *GREM1* promoter (4.3 kbps) and a truncated human *GREM1* promoter (3.6 kbps) that lacks the FOXL1-binding site (n = 4). Mean ± SEM. Mann-Whitney *U* test (*B*), 2-tailed unpaired Student *t* test (*D*, *F*, *G*, and *J*), and 2-way ANOVA with Tukey post hoc multiple comparisons (*K*). Scale bars, 50 μm. ORF, open reading frame; TSS, transcriptional start site.

We next sought to characterize the specific stromal cell types expressing *Grem1* and *Islr* in the normal colon. To this end, we performed *Grem1* and *Islr* ISH using normal mouse colons (Figure 3A and B). As shown elsewhere,^14^ our ISH data confirmed that *Grem1* expression was observed in fibroblastic cells near the base of the colonic crypts in the lamina propria, as well as in muscularis mucosae cells. Interestingly, however, *Islr*^+^ fibroblasts were located near the middle of the colonic crypts, suggesting that *Grem1*^+^ intestinal fibroblasts were topographically distinct from *Islr*^+^ intestinal fibroblasts in the normal colonic crypts.

To further define the fibroblast subpopulations expressing *Grem1* and *Islr*, we studied *Grem1*-CreERT2^6^;Rosa2^6^-LSL-tdtomato mice and *Islr*-CreERT2^17^;Rosa26-LSL-tdtomato mice and costained with intestinal fibroblast markers (Figure 3C and D). A recent report has illustrated that subepithelial telocytes identified by expression of *Foxl1* and platelet-derived growth factor receptor alpha (*Pdgfra*) provide key intestinal stem cell niche signaling molecules such as Wnts and *Grem1*.^21^ Indeed, analysis of RNA-seq data from FACS-purified *Foxl1*-lineage intestinal telocytes and *non–Foxl1*-lineage intestinal mesenchymal cells^21^ suggested that *Grem1* expression was observed in *Foxl1*-lineage^+^ telocytes, but not in *Foxl1*-lineage^−^ cells (Supplementary Figure 10). Consistent with this, *Foxl1* smFISH or PDGFR*α* immunofluorescence (IF) using *Grem1*-CreERT2 mice showed that the majority of the *Grem1*^+^ fibroblasts, also known as *Grem1*^+^ intestinal reticular stem cells (iRSCs),^6^ expressed the telocyte markers *Foxl1* and PDGFR*α* in the lamina propria of the normal colon (Figure 3C and D). In contrast, *Islr*^+^ fibroblasts exhibited lower positivity for *Foxl1* and PDGFR*α* than *Grem1*^+^ iRSCs. These data implied a high degree of overlap between *Grem1*^+^ iRSCs and *Foxl1*^+^ telocytes. This prompted us to investigate whether *Foxl1* might drive *Grem1* expression at the expense of *Islr* expression.

### *FOXL1* Directly Up-regulates *Grem1* Transcription While Repressing *Islr* Expression in Mouse Colonic Fibroblasts

To assess the effect of FOXL1 on the regulation of *Grem1* and *Islr* expression, human FOXL1-overexpressing YH2 cells were generated by lentiviral transduction (Figure 3E). Consistent with our earlier colocalization analyses, FOXL1 overexpression in YH2 cells induced *Grem1* up-regulation at the expense of *Islr* expression, accompanied by decreased *Id2* expression (Figure 3F). Similarly, luciferase reporter assays showed that FOXL1 overexpression increased the activity of the GREM1-promoter reporter and reduced the activity of the *ISLR*-promoter reporter in comparison to control empty YH2 lines (Supplementary Figure 11). Conversely, clustered regularly interspaced short palindromic repeats (CRISPR)/Cas9–mediated Foxl1-knockdown in mouse primary colonic fibroblasts attenuated *Grem1* expression while inducing up-regulation of *Islr* and BMP target genes (Figure 3G and Supplementary Figure 12).

To explore whether FOXL1 is directly involved in regulating *Grem1* expression, we generated *FOXL1*-hemagglutinin (HA) tag-overexpressing YH2 cells (Figure 3H) and performed chromatin immunoprecipitation (ChIP) using an anti-HA antibody. ChIP–quantitative polymerase chain reaction (PCR) showed enrichment of FOXL1-HA binding to the *Grem1* promoter (transcriptional start site, +2008 to +2014 bps; an intronic region) compared to immunoglobulin controls (Figure 3I and J). Consistent herewith, a luciferase assay using the human *GREM1* promoter region in YH2 cells confirmed that FOXL1-mediated augmentation of *GREM1* expression was abrogated by truncation of the *GREM1* promoter to remove the FOXL1-binding region (Figure 3K). Collectively, these data indicate that FOXL1 is recruited to the *Grem1/GREM1* promoter to drive *Grem1* expression, providing mechanistic insight into the overlap between *Grem1*^+^ iRSCs and *Foxl1*^+^ telocytes.

### *GREM1*^+^ Cancer-Associated Fibroblasts Are Myofibroblastic Cancer-Associated Fibroblasts, Which Are Distinct From *ISLR*^+^ Cancer-Associated Fibroblasts, in Mouse and Human Colorectal Cancer

**Figure 4:**
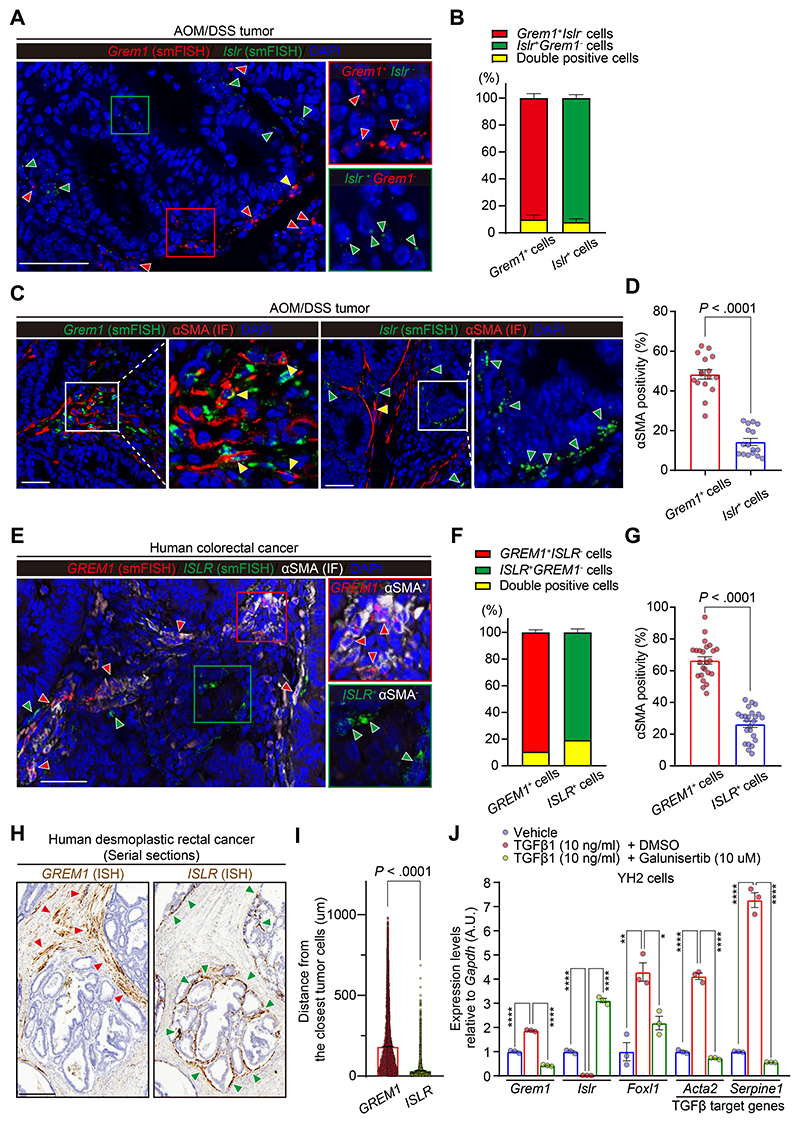
*GREM1*^+^ CAFs are myofibroblastic CAFs, which are distinct from *ISLR*^+^ CAFs in human and mouse CRC. (*A*, *B*) Dual smFISH for *Grem1* and *Islr* in an azoxymethane/dextran sodium sulfate mouse model of CRC. (*A*) Representative pictures. Red, green, and yellow arrowheads denote *Grem1*^+^*Islr*^−^, *Grem1*^−^*Islr*^+^, and *Grem1*^+^*Islr*^+^ CAFs, respectively. (*B*) Semiquantification of the ratio of double-positive (*Grem1*^+^*Islr*^+^) cells in *Grem1*^+^ cells and *Islr*^+^ cells. Four high-power fields (400×)/mouse, 4 mice. (*C*, *D*) *Grem1* smFISH or *Islr* smFISH followed by *α*SMA IF in azoxymethane/dextran sodium sulfate tumors. (*C*) Representative pictures. Yellow and green arrowheads indicate double-positive cells (*Grem1*^+^*α*SMA^+^ cells or *Islr*^+^*α*SMA^+^ cells) and *Islr*^+^*α*SMA^−^ cells, respectively. (*D*) *α*SMA positivity in *Grem1*^+^cells and *Islr*^+^ cells. Four high-power fields/mouse, 4 mice each. (*E*–*G*) Dual smFISH for *GREM1* and *ISLR* followed by *α*SMA IF in human CRC. (*E*) Representative pictures. Red and green arrowheads denote *GREM1*^+^*α*SMA^+^ cells and *ISLR*^+^*α*SMA^−^ cells, respectively. (*F*) Semiquantification of the ratio of double-positive (*GREM1*^+^*ISLR*^+^) cells in *GREM1*^+^ cells and *ISLR*^+^ cells. (*G*) *α*SMA positivity in *GREM1*^+^ cells and *ISLR*^+^ cells; 4–6 high-power fields/patient, 5 patients. (*H*, *I*) *GREM1*^+^ CAFs are spatially distinct from *ISLR*^+^ CAFs in human desmoplastic rectal cancer. (*H*) Representative pictures of *GREM1* and *ISLR* ISH on human desmoplastic rectal cancer samples. Red and green arrowheads denote *GREM1* and *ISLR* expression, respectively. (*I*) Quantification of the minimum distance between *GREM1* or *ISLR* ISH signals and the closest tumor cells; n = 38,396 (*GREM1*) and 18,028 DAB^+^ signals (*ISLR*) from 3 low-power fields (100×)/patient, 7 patients each. (*J*) YH2 cells were stimulated with a vehicle, recombinant TGF-*β*1, or recombinant TGF*β*1 + Galunisertib for 24 hours, followed by qRT-PCR (n = 3). Mean ± SEM. Mann-Whitney *U* test (*D*, *G*, and *I*) and 1-way ANOVA with Tukey post hoc multiple comparisons (*J*). The boxed areas are magnified in the adjacent panels (*A*, *C*, and *E*). Scale bars, 50 *μ*m (*A*, *C*, and *E*) and 250 *μ*m (*H*). ****P < .0001, **P = .0013, *P = .0122. AOM, azoxymethane; DMSO, dimethyl sulfoxide; DSS, dextran sodium sulfate; M, mol/L.

Next, we sought to characterize *GREM1*^+^ CAFs and *ISLR*^+^ CAFs in the CRC mesenchyme. Using azoxymethane/dextran sulfate sodium mouse CRC and human CRC samples, we carried out *Grem1/GREM1* and *Islr/ISLR* smFISH as well as IF for *α*SMA, a well-established marker of myofibroblastic CAFs. We found that, both in mouse and human CRC, *Grem1/GREM1*^+^ CAFs were distinct from *Islr/ISLR*^+^ CAFs and that *α*SMA positivity was higher in *Grem1/GREM1*^+^ CAFs than *Islr/ISLR*^+^ CAFs (Figure 4A–G). A collagen gel contraction assay also showed that Grem1-overexpressing YH2 cells exhibited increased contraction, a hallmark of activated myofibroblasts^2^ (Supplementary Figure 13A and B). Consistent with our smFISH data, scRNA-seq data from human CRC tissues^20^ confirmed that *GREM1* expression levels were inversely correlated with *ISLR* expression (Supplementary Figure 14A) and that *GREM1*, but not *ISLR*, transcripts were positively correlated with *ACTA2* expression in CRC CAFs (Supplementary Figure 14B). Furthermore, the scRNA-seq data set showed that *GREM1* was predominantly expressed by myofibroblasts. High-*ISLR* transcripts were observed not only in myofibroblasts but also in Stromal 2 fibroblasts that are characterized by spatial proximity to epithelial cells and high expression of BMP ligands, including BMP7^20^ (Supplementary Figure 14C and D). Interestingly, *GREM1*^+^ CAFs were spatially distinct from *ISLR*^+^ CAFs in desmoplastic human CRC, with *ISLR*^+^ CAFs located in closer proximity to cancer cells than *GREM1*^+^ CAFs (Figure 4*H* and *I*).

As TGF-*β*1 has well-characterized functions in inducing myofibroblastic differentiation of CAFs,^2^ we examined whether TGF-*β*1 is involved in controlling the differential expression of *Grem1* and *Islr*. Stimulation of YH2 cells with recombinant TGF-*β*1 increased transcript levels of TGF-*β* target genes *Serpine1* and *Acta2*, but also *Foxl1* and Grem1, while decreasing *Islr* expression. This was rescued by cotreatment with Galunisertib, a specific inhibitor for TGF-*β* receptor 1 (Figure 4J). In keeping with the TGF-*β*-induced upregulation of *Foxl1* and *Grem1* in vitro, *GREM1*^+^ CAFs showed a higher degree of colocalization with *FOXL1* in human CRC sections than *ISLR*^+^ CAFs did (Supplementary Figure 15). Together, our data suggest that TGF-*β*1 up-regulates *Foxl1* in fibroblasts that, in turn, bind to the *Grem1* promoter to up-regulate *Grem1* expression. TGF-*β*1 and Foxl1 also reduce the expression of *Islr*. This signaling pathway may be involved in the selective development or differentiation of *α*SMA^+^*Grem1*^+^ myofibroblastic CAFs, which are distinct from *Islr*^+^ *α*SMA^-^ CAFs, in mouse and human CRC.

### Blocking Bone Morphogenetic Protein Antagonism by Using a GREM1-Neutralizing Antibody Promotes Colorectal Cancer Organoid Differentiation and Restrains Growth

What relevance do these stromal changes have on the cancer? We investigated whether augmenting BMP signaling either by *Grem1* inhibition or *Islr* overexpression could retard CRC progression. To this end, we took advantage of CRISPR/Cas9 genome engineering and generated luciferase-expressing *Apc*^Δ/Δ^, *Trp53*^Δ/Δ^ mouse CRC organoids (henceforth referred to as *AP tumoroids*) (Supplementary Figure 16A and B). To disrupt BMP signaling, we sequentially mutated *Smad4*, a downstream effector of BMP signaling, to generate *Smad4*-mutant AP tumoroids (hereafter termed *APS tumoroids*) (Supplementary Figure 16C and D).

First, to test the role of GREM1 in CRC organoid growth, conditioned medium (CM) from *Grem1*-YH2 cells or control green fluorescent protein (GFP)–YH2 cells was transferred to either AP or APS tumoroids (Supplementary Figure 17A). As expected, the expression of BMP target genes was repressed by CM transfer from Grem1-YH2 cells only in AP tumoroids but not in APS tumoroids (Supplementary Figure 17B). Luciferase activity was used to assess an effect of the treatment on viable cell number in luciferaseexpressing AP or APS tumoroid cultures. CM from *Grem1*-YH2 cells increased the tumoroid-derived luciferase signals in AP tumoroids (Supplementary Figure 17C). This was ameliorated by the loss of *Smad4* in APS tumoroids, indicating that the pro-proliferation effect of GREM1 occurred via antagonism of BMP signaling. Consistent with the role of BMP signaling in promoting epithelial cell differentiation,^4,22^ CM from Grem1-YH2 cells decreased the expression of *Krt20*, a marker for differentiated CRC cells, in AP tumoroids but not in APS tumoroids (Supplementary Figure 17D).

**Figure 5:**
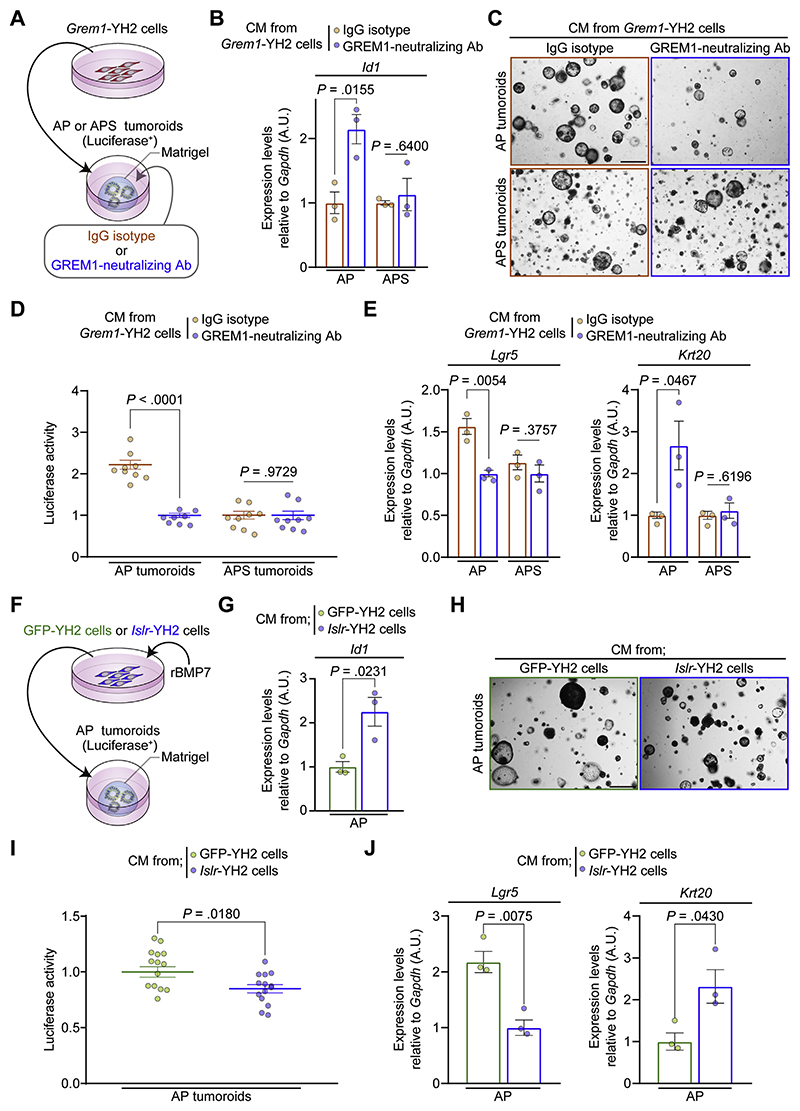
A GREM1-neutralizing antibody or CM from Islr-overexpressing intestinal fibroblasts restrains CRC tumoroid growth and promotes *Lgr5*^+^ stem cell differentiation via increased BMP signaling in tumoroids. (*A*) Experimental schematic depicting CM transfer from *Grem1*-overexpressing YH2 cells to AP (Apc^Δ/Δ^ and *Trp53*^Δ/Δ^) tumoroids or APS (*Apc*^Δ/Δ^, *Trp53*^Δ/Δ^, and *Smad4*^Δ/Δ^) tumoroids. Either an IgG isotype or a GREM1-neutralizing antibody was added to the tumoroids. (*B*) qRT-PCR for *Id1* in AP and APS tumoroids (n = 3). (*C*) Representative pictures of AP tumoroids and APS tumoroids. (*D*) Luciferase signals from AP tumoroids and APS tumoroids (n ≥ 8). (*E*) qRT-PCR for *Lgr5* and *Krt20* in AP and APS tumoroids (n = 3). (*F*) Experimental schematic depicting CM transfer from Islr-overexpressing YH2 cells to AP tumoroids. CM was collected from Islr- or GFP-overexpressing YH2 cells incubated with 10 ng/mL of recombinant BMP7 (rBMP7). (*G*) qRT-PCR for *Id1* in AP tumoroids (n = 3). (*H*) Representative pictures of AP tumoroids. (*I*) Luciferase signals from AP tumoroids (n = 14). (*J*) qRT-PCR for *Lgr5* and *Krt20* in AP tumoroids (n = 3). Scale bars, 500 *μ*m. Mean ± SEM. Two-tailed unpaired Student *t* test (*B*, *D*, *E*, *G*, *I*, and *J*). Note that data normalization was performed within the AP and APS tumoroid groups separately (*B*, *D*, and *E*). Ab, antibody.

We next examined whether restoring BMP signaling with a GREM1-neutralizing antibody could repress CRC organoid growth. For this purpose, either a GREM1-neutralizing antibody or an IgG isotype was added to the AP and APS tumoroids incubated in CM from Grem1-YH2 cells (Figure 5A). The effect of the GREM1-neutralizing antibody to abolish GREM1-mediated BMP antagonism was validated by quantitative reverse-transcription PCR (qRT-PCR) of *Id2* in YH2 cells (Supplementary Figure 18A and B). Blocking the antagonism of BMP signaling by GREM1 using the GREM1-neutralizing antibody restored BMP target gene expression (Figure 5B) and reduced tumoroid-derived luciferase signals and tumoroid size in AP tumoroids but not in APS tumoroids (Figure 5C and D and Supplementary Figure 19A). Moreover, treatment with the GREM1-neutralizing antibody decreased *Lgr5* expression with a concomitant increase in *Krt20* expression in AP tumoroids. This effect was abrogated in APS tumoroids (Figure 5E). Collectively, our in vitro data suggest that restoring BMP signaling with the GREM1-neutralizing antibody promoted differentiation of *Lgr5*^+^ intestinal stem cells and attenuated tumoroid growth of *Smad4*–wild-type, but not *Smad4*-mutant, CRC.

### Conditioned Medium From *Islr*-Overexpressing Intestinal Fibroblasts Increases Bone Morphogenetic Protein Signaling, Facilitates Colorectal Cancer Organoid Differentiation, and Attenuates Growth

Next, we tested whether CM from *Islr*-overexpressing YH2 cells could augment BMP signaling in CRC tumoroids and thus inhibit CRC tumoroid growth. Given that ISLR overexpression promoted BMP signaling in the presence of recombinant BMP7 (Figure 1F and G), we collected CM from *Islr*- or GFP-overexpressing YH2 cells incubated with recombinant BMP7 and transferred the CM to AP tumoroids (Figure 5F). In keeping with our hypothesis of the opposing roles of GREM1 and ISLR, CM from *Islr*-YH2 cells increased *Id1* expression in AP tumoroids, suggesting that ISLR overexpression in fibroblasts enhanced BMP signaling in CRC tumoroids in a paracrine manner (Figure 5G). Moreover, medium conditioned by *Islr*-YH2 cells decreased AP tumoroid-derived luciferase signals and tumoroid size (Figure 5H and I and Supplementary Figure 19B). Similar to the GREM1-neutralizing antibody, this tumoroid growth inhibition by *Islr*-YH2 cell media was accompanied by a reduction in *Lgr5* transcripts and increased *Krt20* expression in AP tumoroids (Figure 5J). Taken together, our in vitro studies indicate that modulating stromal, secreted BMP regulators of BMP signaling through either GREM1-neutralizing antibody or *Islr*-overexpression facilitates *Lgr5*^+^ intestinal stem cell differentiation and diminishes CRC tumoroid growth.

### Modifying the Metastatic Niche by Adeno-associated Virus 8–Mediated In Vivo Overexpression of *Islr* in Hepatocytes Retards Colorectal Cancer Hepatic Metastasis

**Figure 6:**
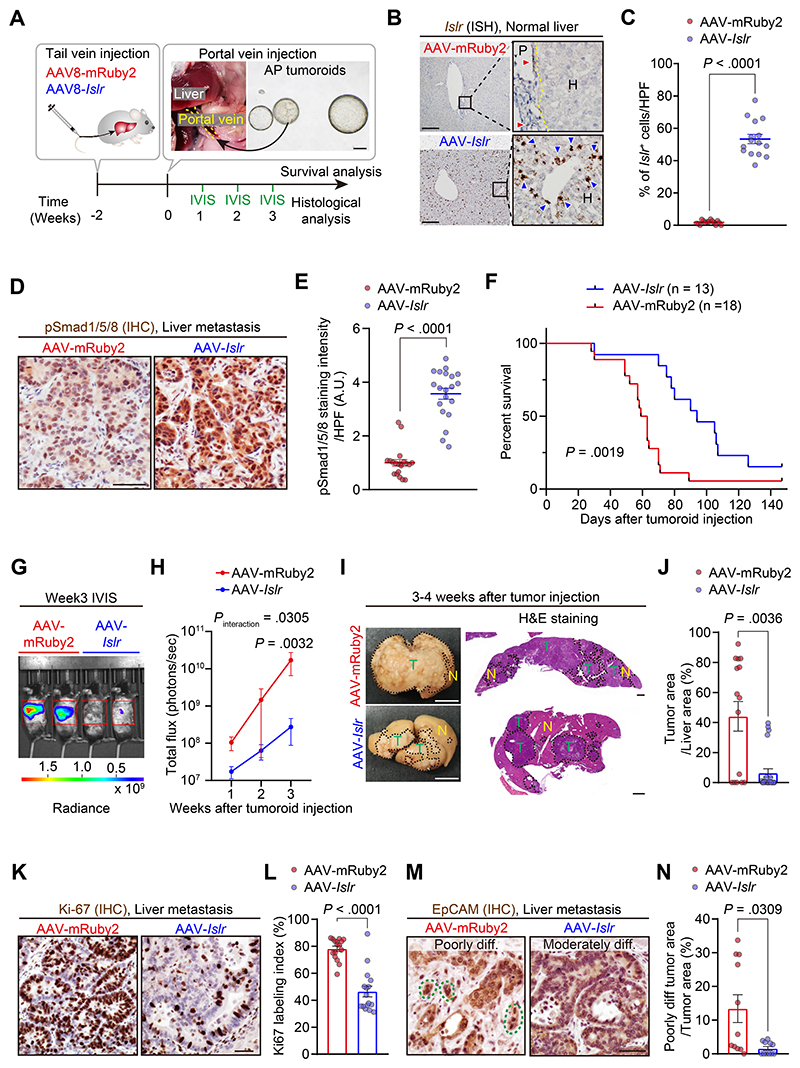
AAV8-mediated *Islr* overexpression in hepatocytes augments BMP signaling and retards CRC hepatic metastasis growth. (*A*) Experimental scheme. Yellow dotted lines outline the portal vein. (*B*, *C*) ISH for *Islr* in the liver 2 weeks after tail vein injection of AAV8-*Islr* or AAV8-mRuby2. (*B*) Representative images. Red and blue arrowheads denote the endogenous expression of *Islr* in fibroblastic cells in the portal area and ectopic overexpression of *Islr* in hepatocytes, respectively. The yellow dotted line indicates the border between the portal area (P) and hepatocytes (H) (*C*) Semiquantification; 5 high-power fields (400×)/mouse, 3 mice each. (*D*, *E*) Immunohistochemistry for pSmad1/5/8 in liver metastases. (*D*) Representative pictures. (*E*) Quantification of 3,3′-diaminobenzidine (DAB) intensity: 5 high-power fields/mouse, 4 mice each. (*F*) Kaplan-Meier survival curves. (*G*, *H*) Luciferase signals from AP tumoroids were assessed by an in vivo imaging system (IVIS). (*G*) Representative images. (*H*) Growth kinetics. Signals within red rectangles in *F* were quantified; n = 5 (AAV-mRuby2) and 8 (AAV-*Islr*) mice. (*I*) Representative macroscopic pictures and H&E-stained sections of liver metastases. Dotted lines indicate borders between tumors (T) and the normal liver (N). (*J*) Quantification of tumor areas using H&E stained sections; 3 liver pieces/mouse, 5 (AAV-mRuby2) and 8 (AAV-Islr) mice. (*K*, *L*) Immunohistochemistry for Ki-67: (*K*) representative pictures and (*L*) Ki-67 positivity in total epithelial cells. Four high-power fields/mouse, 4 mice each. (*M*, *N*) Evaluation of tumor cell differentiation status. (*M*) Representative pictures of immunohistochemistry for EpCAM. Green dotted lines indicate tumor budding. (*N*) The ratio of poorly differentiated tumor areas in the total tumor areas; 11 liver pieces each group from 5 (AAV-mRuby2) and 8 (AAV-*Islr*) mice. Scale bars represent 200 *μ*m (*A*, *B*), 1 cm (macroscopic pictures in *I*), 1 mm (H&E staining in *I*), and 50 *μ*m (*D*, *K*, and *M*). Mean ± SEM. Mann-Whitney *U* test (*C*, *E*, *J*, *L*, and *N*), log rank test (*F*), and 2-way repeated-measures ANOVA with post hoc Sidak multiple comparison test at week 3 (*H*). diff, differentiated; IHC, immunohistochemistry; IVIS, in vivo imaging system.

Hepatic metastasis is the major cause of CRC death,^1^ and, through portal vein dissemination, complicates most advanced gastrointestinal adenocarcinomas. Therefore, we explored whether enhancing BMP signaling, either by the GREM1-neutralizing antibody or *Islr* overexpression, could impair hepatic metastagenesis of CRC in vivo. For this purpose, we generated a mouse model of CRC hepatic metastasis using intraportal injection of AP tumoroids (Figure 6A).

Initially, we examined whether treatment with the GREM1-neutralizing antibody could retard CRC hepatic metastasis and improve survival in comparison to an IgG isotype–treated control group (Supplementary Figure 20A). Consistent with our earlier observation in human CRC liver metastases (Supplementary Figure 8A and B), ISH for *Grem1* confirmed that *Grem1* was expressed by fibroblastic cells in the stroma of mouse CRC hepatic metastases (Supplementary Figure 20B). Immunohistochemistry for pSmad1/5/8 showed that the treatment with GREM1-neutralizing antibody restored BMP signaling in metastatic CRC (Supplementary Figure 20C and D). The GREM1-neutralizing antibody–treated group showed a trend toward prolonged overall survival and a trend toward decreased tumor growth, although the groups, as dosed in this study, did not show significant differences in survival (Supplementary Figure 20E and F).

Thus, we then focused on the other key stromal BMP signaling regulator, *Islr*. Inspired by recent advances in AAV-mediated gene therapy in human diseases,^23,24^ we reasoned that augmenting BMP signaling, via ectopic overexpression of *Islr* in hepatocytes, a liver cell type shown to contribute to a metastatic niche,^25^ could potentially ameliorate the progression of CRC hepatic metastasis. We injected AAV8 (an AAV serotype with tropism for murine hepatocytes^26^) encoding either *Islr* or, as a control, a red fluorescent protein (mRuby2) via mouse tail vein (Figure 6A) to generate ectopic *Islr* overexpression in hepatocytes in vivo (Figure 6B and C). Two weeks after the tail vein injection, AP tumoroid cells were injected directly into the portal vein to generate CRC hepatic metastases. The liver-directed delivery of *Islr* enhanced BMP signaling in CRC hepatic metastasis as well as in normal hepatocytes (Figure 6D and E and Supplementary Figure 21A–C) and significantly prolonged mouse survival compared to the AAV8-mRuby2 control group (Figure 6F). Notably, there was no histologic evidence of liver injury induced by AAV8-*Islr* (Supplementary Figure 22A and B).

To evaluate alterations in growth kinetics and histopathology by AAV8-*Islr*, we next monitored the growth of CRC hepatic metastases with an in vivo imaging system and harvested all mice 3 to 4 weeks after tumor injection (Figure 6A). In line with improved survival, the AAV8-*Islr* group showed reduced tumor-derived luminescence signal, histologic tumor area, and Ki-67 cell proliferation index (Figure 6G–L). Consistent with our earlier in vitro data showing that stromal *Islr* overexpression promoted CRC tumoroid differentiation, *Islr* overexpression in hepatocytes yielded more differentiated CRC histology (Figure 6M and N). Furthermore, in agreement with antifibrotic roles of BMP7 signaling,^17^ *α*SMA immunostaining and Picro-Sirius red staining showed that fibrosis was reduced in the AAV8-Islr group (Supplementary Figure 23A–D). Taken together, these data suggest that BMP modulation could be an attractive target in CRC metastasis and that leveraging hepatocytes to augment BMP signaling by AAV8-*Islr* could represent an exciting, novel therapeutic opportunity in metastatic CRC.

## Discussion

Initially identified more than 50 years ago,^27^ BMP is now known to be important in regulating intestinal epithelial homeostasis and cancer cell proliferation.^3,4^ The regulation of BMP in the tumor microenvironment and the role of BMP in tumor management, however, are still largely unknown. In the present study, we have shown that *GREM1* and *ISLR* are CAF-specific factors that exert opposing effects on BMP signaling in colonic fibroblasts and define distinct sub-populations of fibroblasts in the normal colon and CRC. FOXL1 and TGF-*β* may explain, at least in part, the polarization of CAFs into tumor-promoting *GREM1*^+^ CAFs and tumor-retarding *ISLR*^+^ CAFs. Moreover, *GREM1* and *ISLR* expression levels were associated with poor and favorable outcomes in patients with CRC. Using organoid culture and a preclinical mouse model, our data support that BMP signaling imbalance, regulated by *Grem1* and *Islr*, drives CRC progression and is a key target for cancer treatment. We provided the therapeutic proof of principle that augmenting BMP signaling, either by using a GREM1-neutralizing antibody or AAV8-Islr, represents an attractive future approach to treat CRC.

Previous studies have shown that stromal deletion of BMPR2 could facilitate carcinogenesis in CRC and breast cancer, suggesting tumor-suppressive functions of mesenchymal BMP signaling.^28,29^ Moreover, augmentation of BMP signaling mediated either by increased BMP ligand or decreased BMP antagonist secretion from the tumor mesenchyme, which occurs as a consequence of stromal Hedgehog signaling activation, restrains bladder cancer or CRC progression.^22,30^ Our work supports these findings and provides novel insights into regulatory mechanisms of BMP signaling in the CRC tumor microenvironment. Given that loss of stromal BMPR2 expression increases cytokine production in a mouse model of breast cancer,^28^ further studies are warranted to investigate whether mesenchymal BMP signaling, modulated by *GREM1* and *ISLR*, could also be involved in shaping the immunosuppressive tumor microenvironment.

High stromal TGF-*β* signaling is associated with worse outcomes for patients with CRC.^19^ Here, we provide a potential mechanism underlying CAF heterogeneity, initiated by TGF-*β* and FOXL1, that polarizes fibroblasts toward *Grem1*^high^*Islr*^low^ CAFs or *Islr*^high^*Grem1*^low^ CAFs. TGF-*β* drives *Foxl1* expression in CAFs (Figure 4f). FOXL1, in turn, directly up-regulates *Grem1* expression to antagonize BMP signaling (Figure 3E–K) and promote cancer progression by suppressing differentiation of *Lgr5*^+^ stem cells while inducing epithelial proliferation. In contrast, relatively low levels of TGF-*β* in the microenvironment of some tumors result in lower levels of FOXL1, which permits higher *Islr* expression and a relative tumorsuppressive and higher-BMP signaling milieu (Figures 3E–G and 4f). This potential TGF-*β*–FOXL1–*Grem1/Islr* axis that modulates BMP signaling in the colon provides a novel mechanism, to our knowledge, to help understand the polarization of CAFs within the tumor microenvironment and presents a promising target for future cancer treatment.

By using scRNA-seq from normal mouse small intestines, one recent article suggested that *Grem1* is expressed mainly by *Pdgfra*^low+^*Cd81*^+^ trophocytes that are distinct from *Foxl1*^+^ telocytes.^5^ Consistent with this, our *Foxl1* smFISH in *Grem1*-CreERT2 mice showed that there were fewer *Foxl1* and *Grem1* double-positive fibroblasts in the small intestine than in the colon (Supplementary Figure 24). This observation raises the possibility of organ-dependent *Foxl1* expression patterns within the context of gastrointestinal fibroblast heterogeneity, which warrants further research.

One limitation of the present study is that we have not unraveled the origins and lineage hierarchy of *Grem1*^+^ iRSCs and *Islr*^+^ fibroblasts despite presenting a potential mechanism for fibroblast polarization. Whether *Grem1*^+^ CAFs and *Islr*^+^ CAFs arise from their local progenitors, are recruited from the bone marrow, or are simply new expression profiles within existing cells requires further investigation. Considering the possible plasticity of CAFs,^2^ it is plausible that *Grem1*^+^ CAFs and *Islr*^+^ CAFs could undergo phenotypic interconversion during tumor development, a state of dynamic flux between a relatively polarized cancer-retarding or cancer-promoting microenvironment.

We also provided the first experimental evidence that the GREM1-neutralizing antibody promoted differentiation of *Lgr5*^+^ intestinal stem cells and retarded CRC tumoroid growth only in a *Smad4*–wild-type setting, but not in *Smad4*-mutant tumoroids. Our data reinforce the importance of stratifying patients who may benefit from the GREM1-neutralizing antibody according to the mutation status of BMP-related genes such as *SMAD4* and BMP receptors in future preclinical and clinical trials. Our preliminary data with an in vivo experimental model of CRC hepatic metastases implied that the GREM1-neutralizing antibody still requires further optimization of the therapeutic setting (metastatic prevention vs treatment), dosage regimens, route of administration, and in combination with other agents. However, the combined in vitro and in vivo findings are encouraging.

To our knowledge, our study is the first to use AAV8 to target hepatocytes to treat hepatic metastasis.^23,31^ Clinical trials have shown that in vivo gene delivery by AAV holds great promise in patients with nonneoplastic diseases such as inherited genetic diseases and degenerative neuromuscular disorders.^23^ Excitingly, recent clinical trials have shown that hepatocyte-directed gene transfer of coagulation factors by AAV substantially improved clinical symptoms in patients with hemophilia.^23,24^ In light of new human liver-tropic bioengineered AAVs,^26^ our work suggests that AAV-mediated hepatocyte-directed therapy could, in the future, serve as a novel and well-tolerated cancer therapy. Furthermore, our findings pave the way for AAV-mediated delivery of payloads to modulate not only BMP signaling but any other number of the relevant biological hallmarks of cancer.^32^

In conclusion, our data show that stromal BMP signaling, inhibited by *GREM1* and promoted by *ISLR*, is biologically relevant in CRC growth, spread, and survival. By targeting the upstream determinants of mesenchymal expression, such as TGF-*β* and FOXL1 or by targeting the downstream drivers of BMP signaling such as GREM1 and ISLR, one may identify new approaches to prevent and treat cancer.

## Supplementary Materials and Methods

Supplementary Materials and Methods

## What You Need to Know

### Background and Context

Colorectal cancer (CRC) progression is promoted or restrained by cancer-associated fibroblasts (CAFs). However, the biological mechanisms underlying CAF heterogeneity and therapeutic strategies to target the CRC stroma remain largely unexplored.

### New Findings

Mesenchymal BMP signaling increased by ISLR and decreased by GREM1 regulates CRC progression. Augmenting BMP signaling by adeno-associated virus (AAV)-mediated delivery of *Islr* to hepatocytes inhibited CRC hepatic metastasis growth.

### Limitations

This study was performed using mouse models and human tissue samples. Future studies are necessary to determine the therapeutic efficacy of modulating stromal BMP signaling in patients with CRC.

### Impact

Targeting BMP signaling in the mesenchyme by novel approaches such as AAV-directed gene delivery to the liver, could prove effective adjuvant approaches to treat CRC.

## Abbreviations used in this paper

*α*SMA: *α* smooth muscle actin
AAV: adeno-associated virus
AP: *Apc*^Δ/Δ^ and *Trp53*^Δ/Δ^
APS: *Apc*^Δ/Δ^, *Trp53*^Δ/Δ^, and *Smad4*^Δ/Δ^
BMP: bone morphogenetic protein
CAF: cancer-associated fibroblast
ChIP: chromatin immunoprecipitation
CRISPR: clustered regularly interspaced short palindromic repeats
CM: conditioned medium
CRC: colorectal cancer
EpCAM: epithelial cell adhesion molecule
FACS: fluorescence-activated cell sorting
FAP: fibroblast activation protein alpha
GFP: green fluorescent protein
GO: Gene Ontology
HA: hemagglutinin
IF: immunofluorescence
iRSC: intestinal reticular stem cell
ISH: in situ hybridization
Meflin: mesenchymal stromal cell- and fibroblast-expressing Linx paralogue
PCR: polymerase chain reaction
qRT-PCR: quantitative reverse-transcription polymerase chain reaction
scRNA-seq: single-cell RNA sequencing
SEM: standard error of the mean
smFISH: single-molecule fluorescent in situ hybridization
TGF-*β*: transforming growth factor *β*
