# Supplementary material for: The Balance of Stromal BMP Signaling Mediated by GREM1 and ISLR Drives Colorectal Carcinogenesis
Source: Gastroenterology. Author manuscript; Available in PMC 2024 Dec 9. (PMC7617122; doi:10.1053/j.gastro.2020.11.011)
Supplement: Supplementary Materials and Methods [file EMS181861-supplement-Supplementary_Materials_and_Methods.pdf]

Supplementary Materials and Methods

Human and Animal Ethics

Human CRC samples were obtained at the time of surgery from patients who had provided informed consent. This study was approved by the Ethics Committee of Nagoya University Graduate School of Medicine (2017-0127). All animal protocols were approved by the Animal Ethics committees of SAHMRI and Nagoya University Graduate School of Medicine (SAM322, SAM189, and 31434).

Antibodies

The following antibodies were used in this study. Goat polyclonal anti-GREM1 antibody (1:1000 for Western blotting [WB]; AF956, R&D Systems), rabbit polyclonal anti-ISLR antibody (1:1000 for WB; HPA050811, Atlas Antibodies), mouse monoclonal anti-ACTB (1:5000 for WB; sc-47778, Santa Cruz Biotechnology), rabbit polyclonal anti-RFP (1:500 for IF; 600-401-379, Rockland), rabbit monoclonal anti–phospho-Smad1/5 (1:1000 for WB; clone 41D10, no. 9516, Cell Signaling Technology), rabbit polyclonal anti-Smad1 (1:1000 for WB; Cell Signaling Technology, no. 9743), goat polyclonal anti-mouse PDGFRA (1:50 for IF; AF1062, R&D Systems), rabbit polyclonal anti-HA antibody (1:7500 for WB; ab9110, Abcam), rabbit polyclonal anti-FOXL1 (1:1000 for WB; ABC937, Merck Millipore), mouse monoclonal antiαSMA (1:500 for immunohistochemistry [IHC]; clone 1A4, Dako), rabbit monoclonal anti-Smad4 (1:5000 for WB; ab40759, Abcam), rabbit monoclonal anti-Ki67 (1:200 for IHC; ab16667, Abcam), rabbit polyclonal anti-EpCAM (1:150 for IHC; abcam71916, Abcam), sheep polyclonal anti-FAP (1:100 for IF; AF3715, R&D Systems), and rabbit polyclonal anti-phospho-Smad1/5/8 (1:100 for IHC; AB3848-I, Merck Millipore). Mouse monoclonal GREM1-neutralizing antibody (Ab7326) and mouse monoclonal IgG1 isotype (Ab101.4) were provided by UCB Pharma.

RNA In Situ Hybridization

All ISH analyses were performed on formalin-fixed and paraffin-embedded human and mouse tissue samples by using RNAscope technology (RNAscope 2.5 HD Detection Kit or RNAscope Multiplex Fluorescent Reagent Kit v2, Advanced Cell Diagnostics [ACD]) following the manufacturer's instructions. Briefly, tissue sections were baked in a dry oven (HybEZ II Hybridization System, ACD) at 60°C for 1 hour and deparaffinized, followed by incubation with Pretreat 1, 2, and 3 (ACD). Slides were incubated with relevant probes for 1 hour at 40°C, followed by successive incubations with Amp1–6 reagents. Staining was visualized with diaminobenzidine (DAB) or TSA Plus Cyanine 5 (NEL745001KT, PerkinElmer). For survival analysis with human CRC samples, ISH samples were quantified by a clinical pathologist in a blinded manner, following a semi-quantitative scoring method recommended by the manufacturer (ACD), where each signal was evaluated as 0 (no staining or <1 dot/cell), 1 (1–3 dots/cell), 2 (4–9 dots/cell with no or very few dot clusters), 3 (10–15 dots/cell and <10% of dots in clusters), or 4 (>15 dots/cell and >10% of dots in clusters). For other ISH analyses, cells with a score of 0 were defined as negative, and cells with a score of 1 or more were defined as positive. In normal colorectal samples, only lamina propria areas were quantified (Figures 2*B* and 3*B* and *D*).

Probes Used for In Situ Hybridization

ISH (RNAscope) probes used in the study were human *PPIB* (a positive control probe, NM_000942.4, regions 139–989; catalogue number 313901), bacterial *DapB* (a negative control probe, EF191515, regions 414–862; catalogue number 310043), human *ISLR* (NM_005545.3, region 275–1322; catalogue number 455481 or 455481-C2), mouse *Islr* (NM_012043.4, regions 763–1690; catalogue number 450041 or NM_012043.4, regions 277–2225; catalogue number 453321-C2), human *GREM1* (NM_013372.6, regions 175–1472; catalogue number 312831 or 312831-C2), mouse *Grem1* (NM_011824.4, regions 398–1359; catalogue number 314741), human *FOXL1* (NM_005250.2, regions 1610–2981; catalogue number 558081), and mouse *Foxl1* (NM_008024.2, regions 954–1931; catalogue number 407401).

Luciferase Reporter Assay

DNA fragments of 4.3 kilo base pairs (–1524 to +2841; +1 is the transcriptional start site (TSS) of exon 1 of human *GREM1*) spanning human *GREM1* promoter and intron regions were generated by custom gene synthesis by Gene Universal and inserted into the upstream of the luciferase (NanoLuc) reporter gene in the pNL2.1 vector (Promega). This region contained a putative hFOXL1-binding site at TSS +2103 to +2109 (corresponding to TSS +2008 to +2014 in mouse *Grem1*), as analyzed by the JASPAR database.[1] Sequences from TSS +2103 to +2841, which contains the putative hFOXL1-binding site, were deleted from the *GREM1* promoter (4.3 kbps)–NanoLuc plasmid, resulting in the generation of the truncated *GREM1* promoter (3.6 kbps)–NanoLuc vector.

The 3.5–kilo base pair human *ISLR* promoter and intron regions (–2321 to +1179, +1 is the TSS of exon 1 of human *ISLR*) were inserted upstream of the luciferase (NanoLuc) reporter gene in the vector pNL2.1 (Promega), as described previously.[2]

The pNL2.1 vectors containing the *GREM1* or *ISLR* promoter regions were cotransfected with pEF1a (human elongation factor 1 alpha promoter)–firefly into YH2 cells by using Lipofectamine 2000 (11668019, Thermo Fisher Scientific). Cell lysates were collected 48 hours after transfection by using a passive lysis buffer (E1941, Promega). The luminescence values of cell lysates were measured by using a Nano-Glo Dual-Luciferase Reporter Assay System (N1610, Promega) and a GloMax microplate reader (GM3000, Promega) following the manufacturer's instructions. The NanoLuc luminescence levels were normalized to the firefly luminescence levels.

To evaluate BMP signaling activation in GFP-, *Islr*-, and *Grem1*-overexpressing YH2 cells, YH2 cells were cotransfected with pGL3 BMP-responsive element–firefly (Addgene; plasmid no. 45126) and pTK-*Renilla* (E2241, Promega). Cells were grown in Dulbecco's modified Eagle medium (DMEM) containing 1% fetal bovine serum (FBS), and cell lysates were collected 48 hours after transfection by using passive lysis buffer (E1941, Promega). Luminescence values of cell lysates were measured by using a Dual-Luciferase Reporter Assay System (E1960, Promega) and a GloMax microplate reader (GM3000, Promega). The firefly luminescence levels were normalized to the *Renilla* luminescence levels. Each assay was performed with at least 2 technical replicates.

Promoter Analyses and Chromatin Immunoprecipitation

Promoter analyses were performed by using the JASPAR database.[1] Promoter analyses of the genomic area around the *Grem1* TSS for putative FOXL1-binding sites identified a binding motif for FOXL1 at TSS +2008 to +2014 bps, an intronic region of the mouse *Grem1* gene, which was conserved in humans.

Chromatin was prepared from 3 independent replicates of *FOXL1*-HA–overexpressing YH2 cells by using a truChIP Chromatin Shearing Kit with Formaldehyde (520154, Covaris) according to the manufacturer's instructions. Chromatin shearing was performed by using a Covaris M220 focused ultrasonicator. ChIP assay was performed by using the EpiQuik Chromatin Immunoprecipitation Kit (P-2002, Epigentek) and IgG isotype (ab171870, Abcam), anti-HA antibody (ab9110, Abcam), and anti-Histone H3 antibody (ab1791, Abcam) as per the manufacturer's protocol. Prepared DNA samples were analyzed by quantitative PCR with the following primers: mouse *Grem1* promoter (forward: 5′-gcaccgttggattaaggctc-3′; reverse: 5′-tgaagatcataggaaagctgtgaag-3′).

Histopathologic Examination

All histopathologic examinations were performed by using formalin-fixed and paraffin-embedded tissues. Paraffin blocks were cut into 4–5-μm sections. H&E staining and Picro-Sirius red staining were performed by Histology Services (University of Adelaide) or Sept Sapie (Tokyo, Japan).

Immunohistochemistry and Immunofluorescence

Formalin-fixed and paraffin-embedded tissue sections were deparaffinized with xylene and rehydrated with phosphate-buffered saline (PBS), followed by antigen retrieval by boiling samples in antigen retrieval buffer (pH 6 or pH 9; H-3300, Vector Laboratories; S1699, Dako; or S2367, Dako) for 30 minutes. Inactivation of endogenous peroxidase was performed with 0.5% H_2O_2 in methanol for 15 minutes, followed by washing with PBS. Then, sections were treated with blocking buffer (X0909, Dako) for 30 minutes, incubated with the indicated primary antibodies overnight at 4°C, and washed with PBS. Sections were incubated with horseradish peroxidase (HRP)–polymer secondary antibody (ab214879 or ab214880, both from Abcam) for 30 minutes, followed by signal detection with DAB solution (K3468, Dako).

For IF studies, deparaffinized sections were treated with blocking buffer (X0909, Dako) for 30 minutes, incubated with the indicated primary antibodies overnight at 4°C, and washed with PBS. Sections were then incubated with Alexa Fluor 488/594/647–conjugated secondary antibodies (all from Thermo Fisher Scientific) for 1 hour at room temperature. The sections were then mounted with ProLong Gold antifade reagent containing 4′6-diamidino-2-phenylindole (DAPI) (Thermo Fisher Scientific), and fluorescence was examined by using a confocal laser-scanning microscope (TCS SP8 MP, Leica) or an inverse IF microscope BZ-X710 (Keyence) with optical sectioning.

Combined Single-Molecule Fluorescence In Situ Hybridization and Immunofluorescence

Combined smFISH and IF were implemented by first performing smFISH, followed by IF. After smFISH, the sections were blocked with blocking buffer (X0909, Dako) and then incubated with a primary antibody overnight at 4°C. The sections were washed in 1× Phosphate-buffered saline containing 0.05% TWEEN 20 (P1379, Sigma) 3 times and then incubated with Alexa Fluor 555–conjugated secondary antibody (Thermo Fisher Scientific) for 60 minutes at room temperature. The sections were then mounted with ProLong Gold antifade reagent containing DAPI (Thermo Fisher Scientific). Fluorescence was examined by using an inverse IF microscope BZ-X710 (Keyence) with optical sectioning.

Western Blot Analysis

Cells were lysed in a lysis buffer (78501, Thermo Fisher Scientific) supplemented with Complete Protease Inhibitor (Roche) and PhosSTOP Phosphatase Inhibitor cocktails (4906845001, Roche). Lysates were clarified by centrifugation at 12,000*g* for 10 minutes at 4°C. Then, sodium dodecyl sulfate (SDS) sample buffer (10 mmol/L Tris-HCl, 2% SDS, 2 mmol/L EDTA, 0.02% bromophenol blue, 6% glycerol; pH 6.8) was added. To prepare CM and total cell lysates for WB, YH2 cells were incubated for 72 hours in protein-free and FBS-free medium (Freestyle 293 expression medium; 12338018, Gibco). The medium was collected, centrifuged at 400*g* for 5 minutes, and filtered through a 0.45-μm filter (16533, Sartorius) to remove cell debris. The medium was concentrated 10-fold by using Amicon Ultra 3-kDa centrifugal filters (UFC500396, Millipore).

The separation was performed by SDS–polyacrylamide gel electrophoresis using a precast gel (4568094, Bio-Rad). Proteins were then transferred to polyvinylidene difluoride membranes (1620177, Bio-Rad) by using a semidry transfer system (1703940, Bio-Rad). The membranes were blocked in 5% milk in PBS containing 0.05% Tween 20 and then incubated with primary antibodies. Proteins were detected by HRP–conjugated secondary antibodies (NA9310V, GE Healthcare; N934VS, GE Healthcare; ab97120, Abcam), followed by signal development using an

HRP substrate (WBLUR0500, Millipore). The blots were imaged by using ChemiDoc MP (Bio-Rad) and quantified with Image Lab software, version 6.0.1 (Bio-Rad).

Plasmids

The cloning of mouse *Islr* complementary DNA (cDNA) was described previously.[3] A DNA fragment for the codon-optimized mouse *Grem1* gene was generated by using a custom gene synthesis service by Gene Universal. These genes were subcloned into bidirectional pLenti-EF1a–multiple cloning site–PGK-Puro vectors, resulting in the generation of pLenti-EF1a-*Islr*-PGK-Puro and pLenti-EF1a-*Grem1*-PGK-Puro.

To overexpress FOXL1 using a lentiviral expression system, DNA fragments for mouse codon–optimized human *FOXL1* gene with either no epitope tags or 3× HA tags at the amino terminus were generated by using a custom gene synthesis service by Gene Universal. These genes were subcloned into unidirectional pLenti-EF1a-GFP-p2A-Puro-EF1a–multiple cloning site vectors, resulting in the generation of pLenti-EF1a-GFP-p2A-Puro-EF1a-*FOXL1* and pLenti-EF1a-GFP-p2A-Puro-EF1a-*FOXL1*–3× HA.

To enable CRISPR/Cas9–mediated mouse *Foxl1* knockdown, the following mouse nontargeting gRNA (guide RNA) sequence or gRNA sequence targeting mouse *Foxl1* was subcloned into pLentiCRISPR v2 eSpCas9 plasmid by GenScript:

Nontargeting gRNA: 5′- AAAAAGTCCGCGATTACGTC-3′

g*Foxl1*: 5′-GGGCTGTACACGTACAACAG-3′

To overexpress *Islr* using the AAV expression system, mouse *Islr* cDNA was subcloned into a self-complementary AAV plasmid (VPK-430, Cell Biolabs), and a pAAV-CMV-*Islr* vector was generated. As a control, a self-complementary AAV plasmid expressing a red fluorescent protein, mRuby2 (pAAV-CAG-mRuby2, Addgene plasmid no. 99123), was used.

Lentivirus Production and Transduction

293T cells were cotransfected with psPAX2 (Addgene, plasmid no. 12260), pMD2.G (Addgene; plasmid no. 12259), and a lentivirus vector plasmid. At 48 and 72 hours after transfection, viral supernatants were harvested, filtered through a 0.45-μm filter, and concentrated by using Amicon Ultra Centrifugal Filters (Merck Millipore, UFC910024). Concentrated lentivirus particles were used for transduction. At 48 hours after transduction, positively transduced cells were selected with 2–4 μg/mL puromycin if the lentivirus vectors contain a puromycin resistance gene.

Retrovirus Production and Transduction

The 293T cells were cotransfected with the pEQ ecotropic-packaging vector[4] and a retrovirus vector plasmid. At 48 and 72 hours after transfection, viral supernatants were harvested, filtered through a 0.45-μm filter, and concentrated by using an ultracentrifuge (Optima XPN, Beckman Coulter). Concentrated retrovirus particles were used for transduction.

Cell Culture

A mouse colonic fibroblast cell line, YH2 cells,[5] was generously provided by Professor Tony Burgess (The Walter and Eliza Hall Institute of Medical Research). Normal mouse colonic organoids were isolated from a Rosa26-Cas9 mouse (JAX stock no. 024858; C57BL/6 × 129 genetic background) housed under pathogen-free conditions in the SAHMRI Bioresources facility. YH2 cells were cultured in DMEM (Sigma) supplemented with 10% FBS (Gibco).

All cell lines used were routinely screened for *Mycoplasma* contamination by MycoAlert Mycoplasma Detection Kit (LT07-118, Lonza).

In Figure 1*F* and *G* and Supplementary Figures 5 and 6, YH2 cells were serum-starved in DMEM containing 1% FBS for 48 hours before stimulation with recombinant human BMP7 (PHC9541, Thermo Fisher) or BMP2 (cyt-261, Prospec) at the concentration indicated in figure legends. Proteins and RNAs were collected 30 minutes and 24 hours, respectively, after the addition of the recombinant BMP7 or BMP2. In Figure 4*J*, YH2 cells were stimulated with a vehicle, 10 ng/mL of recombinant human TGF-β1 (240-B-002, R&D systems) + DMSO, or 10 ng/mL of recombinant human TGF-β1 + 10 μmol/L of galunisertib (ADV465749242; AChemBlock) in DMEM containing 1% FBS for 24 hours. In Supplementary Figure 18*A* and *B*, recombinant human BMP2 (cyt-261, Prospec), recombinant human GREM1 (provided by UCB Pharma), GREM1-neutralizing antibody (Ab7326), and mouse monoclonal IgG1 isotype (Ab101.4) were used for the experiment.

Isolation of Primary Mouse Colonic Fibroblasts

The isolation and culture of primary mouse colonic fibroblasts were performed by using the protocol described elsewhere[6] with modifications. Briefly, colons were harvested from 8–16-week-old C57BL/6J mice and thoroughly washed with cold PBS. The colon tissues were incubated in PBS supplemented with 3 mmol/L EDTA and 0.05 mmol/L dithiothreitol for 60 minutes at room temperature to remove epithelial cells. After washing the colon 3 times with PBS, the colons were digested in 1 mg of collagenase type IV (17104-019, Gibco), 1 mg of dispase (17105-041, Gibco), and 2000 units of DNase I (D4527, Sigma) in 15 mL of RPMI 5 media (described as follows) at 37°C for 30 minutes. The supernatant was filtered through a 70-μm cell strainer, and the cell suspension was plated on T75 flasks. Cells were cultured in RPMI 5 media: RPMI media (R8756, Sigma) supplemented with 5% FBS (Gibco), 2 mmol/L L-glutamine, 1% penicillin/streptomycin, 10 mmol/L HEPES, 1 mmol/L sodium pyruvate, and 0.05 mmol/L 2-mercaptoethanol.[6] At passage 2, cells were transduced with lentivirus encoding Cas9 protein and nontargeting gRNA or gRNA targeting *Foxl1*. RNA and protein collections were performed at passage 4.

Organoid Culture and Organoid Genome Editing

The basal culture medium for mouse colon organoids was Advanced DMEM (ADMEM)/F12 (Life Technologies) supplemented with 1× gentamicin/antimycotic/antibiotic

(Life Technologies), 10 mmol/L HEPES, 2 mmol/L GlutaMAX, 1× B27 (Life Technologies), and 1× N2 (Life Technologies). The following niche factors were used: 50 ng/mL mouse recombinant epidermal growth factor (Peprotech), 100 ng/mL mouse recombinant noggin (Peprotech), 20% R-spondin-2 CM, and 50% Wnt-3A CM. Organoids were plated in 50 μL growth factor-reduced Matrigel (356231, Corning) on a 24-well dish and 500 μL of the medium was added to organoids

gRNAs specific for each target gene were either previously published or designed de novo by using the CRISPR design tool.[7] *Apc* and *p53* gRNA oligos were cloned into pLentiGuide-CMV-dtomato (modified from Addgene plasmid no. 17452). *Smad4* gRNA oligos were cloned into plentiGuide-Puro (Addgene Plasmid no. 52963).

Guide RNA Sequences. *Apc*: 5′-GGAAGCCTTGTGGGACATGG-3′

Trp53: 5′-GTGTAATAGCTCCTGCATGG-3′[8]

Smad4: 5′-CAAAAGCGATCTCCTCCCGA-3′[9]

Normal colon organoids isolated from a Rosa26-Cas9 mouse (JAX stock no. 024858)[8] were transduced with lentivirus expressing sg*Apc* and sg*Trp53*. Three days later, media was changed to ADMEM (no Wnt, no Rspo, +Nutlin-3a; SML0580, Sigma) to enrich for correctly targeted clones (Supplementary Figure 16). After confirmation of correctly targeted monoclonal lines by amplicon sequence, retrovirus expressing firefly (Retro-SFG-NES-HSV1-tk-GFP-Luciferase)[10] was transduced to the AP ($Apc^{\Delta/\Delta}$, $Trp53^{\Delta/\Delta}$) tumoroid line. Then, a monoclonal line that showed high luciferase signals was selected by evaluating the luciferase signals by a luminometer. The luciferase-expressing AP monoclonal tumoroid was further transduced with lentivirus expressing sg*Smad4* and a puromycin resistance gene. Three days later, media was changed to ADMEM (no Wnt, no Rspo, no Noggin, +puromycin, +Nutlin-3a; SML0580, Sigma) to enrich for correctly targeted clones, followed by handpicking of monoclonal lines.

Handpicked monoclonal organoid lines were screened for loss of function insertions/deletions by using amplicon sequences using the following primers, as described.[11] Overhang sequences are underlined.

Apc forward: 5′-<u>CTGAGACTTGCACATCGCAGC</u>TTAATTCAGGCAAATCCTAAGAGAG-3′

Apc reverse: 5′-<u>GTGACCTATGAACTCAGGAGTC</u>GGTCTGTTTGCCATGAGATTCC-3′

Trp53 forward: 5′-<u>GTGACCTATGAACTCAGGAGTC</u>TAGTGAGGTAGGGAGCGACTTC-3′

Trp53 reverse: 5′-<u>CTGAGACTTGCACATCGCAGC</u>CCAAAGAGCGTTGGGCATGTG-3′

Smad4 forward: 5′-<u>CTGAGACTTGCACATCGCAGC</u>CTGGTGCTCCATTGCTTACT-3′

Smad4 reverse: 5′-<u>GTGACCTATGAACTCAGGAGTC</u>ACTTAATTCCTCGATATTTAAGCTC-3′

To detect a large deletion in *Smad4* in APS tumoroids, PCR was performed by using the following primers: *Smad4* forward: 5′-TTGTGTCAGCTCAGAGTGGGTC-3′ and *Smad4* reverse: 5′-GCAAACCACACGACGATGC-3′. After gel electrophoresis and purification of PCR products, Sanger sequencing for the PCR products was performed to examine mutations.

Conditioned Medium Experiments

In Figure 5*A–E* and Supplementary Figure 17*A–D*, monolayer GFP-overexpressing or *Grem1*-overexpressing YH2 cells were cultured for 72 hours in DMEM (Gibco) containing 1% FBS (Gibco), 1% L-glutamine, and 1% penicillin/streptomycin. In Figure 5*F–J*, monolayer GFP-overexpressing or *Islr*-overexpressing YH2 cells were cultured for 72 hours in DMEM containing 1% FBS, 1% L-glutamine, and 1% penicillin/streptomycin with 10 ng/mL recombinant human BMP7 (PHC9541, Thermo Fisher Scientific). The medium was collected and filtered through a 0.45-μm filter (16533, Sartorius) to remove cell debris. Trypsinized tumoroid fragments equivalent to 2500 cells were plated in 50 ul growth factor-reduced Matrigel (356231, Corning) on a 24-well dish. At 30 minutes later, 500 mL of the CM was added to tumoroids. In Figure 5*A–E*, a mouse GREM1-neutralizing antibody (Ab7326, 100 μg/mL) or a mouse IgG1 isotype (Ab101.4, 100 μg/mL) was added to each well every time CM was added to the tumoroids throughout the course of the experiments.

Organoid area measurements, luciferase assays, and qRT-PCR were performed 8 days and 12 days after plating AP tumoroids and APS tumoroids, respectively, in experiments using *Grem1*-overexpressing YH2 cells. In experiments using *Islr*-overexpressing YH2 cells, organoid area measurements, luciferase assays, and qRT-PCR were performed 7 days after plating AP tumoroids.

Luciferase Assays for Firefly-Expressing Tumoroids

For luciferase assays using firefly-expressing tumoroids (AP tumoroids and APS tumoroids), after the removal of medium, tumoroids and Matrigel were lysed with 200 μL of 2× passive lysis buffer (E1941, Promega). Firefly luminescence values of cell lysates were measured with Luciferase Assay Reagent II (E1960, Promega) and a GloMax microplate reader (GM3000, Promega) following the manufacturer's instructions.

Tumoroid Area Quantification

The images of each well (1 picture/independent replicate) were captured by using an inverted microscope (IX53, Olympus; a 2× objective lens). Each organoid was quantified by using ImageJ software (National Institutes of Health)[12] to outline an organoid shape and measure an organoid area.

Quantitative Polymerase Chain Reaction

Total RNA was extracted by using TRIzol reagent (Invitrogen) and an RNeasy Mini Kit (Qiagen). Purified RNA samples were reverse-transcribed by using Transcriptor Universal cDNA Master (Roche) according to the manufacturer's instructions, followed by the dilution of cDNA at 1:5.

qRT-PCR of the cDNAs was performed with KAPA PROBE or SYBER FAST qPCR Master Mix (KAPABiosystems) and was run on a QuantStudio 7 Flex Real-Time PCR System (Thermo Fisher Scientific). The data were analyzed using the 2-ΔΔCt method and normalized to *Gapdh* expression levels.

Probes or primers (generated by IDT) used in this study are as follows. Primers were designed with the Primer-BLAST online program.[13]

Probes. Mouse *Grem1* (Mm.PT.58.11631114), mouse *Islr* (Mm.PT.58.12037488), mouse *Id1* (Mm.PT.58.6622645.g), mouse *Id2* (Mm.PT.58.13116812.g), mouse *Id3* (Mm.PT.58.29482466.g), mouse *Id4* (Mm.PT.58.6851535), mouse *Acta2* (Mm.PT.58.16320644), and mouse *Serpine1* (Mm.PT.58.6413525), mouse *Gapdh* (Mm.PT.39a.1), mouse *Lgr5* (Mm.PT.58.12492947), and mouse *Krt20* (Mm.PT.58.43092140).

Primers. Mouse *Grem1* (forward: 5′-GCTCTCCTTCGTCTTCCTC-3′, reverse: 5′- AGTG-TATGCGGTGCGATTC-3′), mouse *Islr* (forward: 5′-TGCGAG-CAATCCAGTCCTTAGATG-3′, reverse: 5′-AGCCCAACAAAGCAGGCACAG-3′), mouse *Foxl1* (forward: 5′-GTCGCTCAACGAGTGCTTCG-3′, reverse: 5′-TGCGCCGA-TAATTGCCGTTC-3′), and mouse *Gapdh* (forward: 5′-CCTCGTCCCGTAGACAAAATG-3′, reverse: 5′- TGTAGTT-GAGGTCAATGAAGGG-3′)

Analyses of Publicly Available Complementary DNA Gene Expression Microarray and RNA-Sequencing Data Sets

For GSE39396,[14] microarray data were downloaded by using the GEOquery R package, version 2.54.1. Differential gene expression was analyzed as described in the limma software manual.[15] Briefly, a linear modeling approach was used by the function lmFit, followed by the empirical Bayes estimation through functions eBayes and topTable. Differentially up-regulated genes were defined as genes that showed a $\log_2$ fold change of greater than or equal to 2 and false discovery rate of less than 0.05. The top 150 differentially up-regulated gene probes in FAP^+ CAFs in each group (FAP^+ CAFs vs $EpCAM^+$ cancer cells, FAP^+ CAFs vs $CD31^+$ endothelial cells, and FAP^+ CAFs vs $CD45^+$ immune cells) were selected based on the $\log_2$ fold change. Volcano plots were generated by using the ggplot2 package, version 3.2.1. A human gene list in a Gene Ontology (BMP signaling pathway; GO0030509) was downloaded from AmiGO 2 (http://amigo.geneontology.org/amigo/search/ontology).

Expression microarray data (GSE17538, 39582, and 41258) and RNA-seq data (GSE94072) were obtained from the Gene Expression Omnibus (GEO) (https://www.ncbi.nlm.nih.gov/geo/). For survival analyses, gene expression levels for the following probes were used: 218469_PM_s_at for *GREM1* and 207191_PM_s_at for *ISLR*. Patient information in GSE41258,[16] GSE39582,[17] and GSE17538[18] were also obtained from the GEO. False delivery rate (FDR)–adjusted *P* values were calculated by GEO2R (https://www.ncbi.nlm.nih.gov/geo/geo2r). In GSE41258, normal colon samples, polyp (adenoma) samples, primary colon tumor samples, and liver metastasis samples included in a previous analysis[16] were analyzed by choosing samples with a label of “Included in analysis; Yes” on the GEO website. Optimal cutoff levels for survival analyses were determined by using the X-tile software.[19] Patients with survival time 0 (events at the time of patient enrolment) were not included in survival analysis by Prism 8 software (GraphPad; https://www.graphpad.com/guides/prism/8/statistics/stat_qa_survival_analysis.htm). In GSE94072, fragments per kilobase of exon per million fragments (FPKM) data were directly downloaded from the GEO website (https://www.ncbi.nlm.nih.gov/geo/query/acc.cgi?acc=GSE94072).

Analyses of GREM1 *and* ISLR *Expression Levels in the Consensus Molecular Subtypes of Colorectal Cancer*

For The Cancer Genome Atlas data, the consensus molecular subtype (CMS) labels were downloaded from the supplementary materials of a previous publication.[20,21] Expression values and sample identifiers were downloaded from the Genomic Data Commons data portal (https://portal.gdc.cancer.gov/). Expression *Z*-scores for tumor samples were calculated based on the standard deviation and mean value for the normal colorectal tissues. Violin plots were then generated by using the ggplot2 package, version 3.2.1.

The CMS classifier[20] was applied to the downloaded GSE39582 expression microarray data to classify patients with colon cancer into CMSs. This allowed us to classify 566 colon cancer samples into 70 CMS1 tumors, 177 CMS2 tumors, 97 CMS3 tumors, 107 CMS4 tumors, and 115 unclassified tumors.

Analyses of Publicly Available Single-Cell RNA-Sequencing Data

scRNA-seq data (GSE132465)[22] were analyzed by using BBrowser2, version 2.6.4 (BioTuring). Unique molecular identifier counts available on the GEO website (https://www.ncbi.nlm.nih.gov/geo/) were used for normalization by the software. Processed scRNA-seq data (GSE81861)[23] were directly downloaded from the GEO website, and $\log_2$ (FPKM + 1) values were used to plot *GREM1* and *ISLR* expression.

In Supplementary Figure 4*A*, fibroblasts include myofibroblasts and stromal 1, stromal 2, and stromal 3 fibroblasts.[22] Endothelial cells include tip-like endothelial cells, stalk-like endothelial cells, proliferative endothelial cells, and lymphatic endothelial cells.[22] In Supplementary Figure 4*A* and *B*, immune cells comprise T cells, B cells, myeloid cells, and mast cells.[22,23] In Spearman correlation analyses, given our focus on *GREM1*- or *ISLR*-expressing CAFs, 1156 CRC CAFs that show detectable *GREM1* or *ISLR* transcripts (log-normalized *GREM1* expression of >0 or log-normalized *ISLR* expression of >0) were analyzed among all CRC CAFs (1501 cells; myofibroblasts and stromal 1, stromal 2, and stromal 3 fibroblasts from CRC tissues). Log-normalized expression values in the 1156 CRC CAFs were

transformed to *Z*-scores in the Spearman correlation analyses.

Tamoxifen Administration to Mice

Grem1-CreERT2 (JAX stock no. 027039);Rosa26-LSL (LoxP-stop-LoxP)–tdtomato (JAX stock no. 007909) and *Islr*-CreERT2[2];Rosa26-LSL-tdtomato were subjected to tamoxifen administration. First, 6 mg of tamoxifen (T5648, Sigma) dissolved in peanut oil was administered by oral gavage 4 times every other day to 6-week-old adult mice to sufficiently induce CreERT2-mediated recombination, followed by harvest of the 8-week-old mice. The colons and small intestines were removed, opened longitudinally, and fixed in 10% formalin overnight at room temperature. Tissues were subsequently dehydrated and paraffin embedded, and 4-μm sections were used for histologic analysis (ISH/IF and IF).

Mouse Genotyping

Genomic DNA extracted from mouse ear or tails was used for PCR genotyping. The sequences of the primers were as follows:

Islr–wild type (WT) forward: 5′-ACACACGACCTTGGCAAGTCCCAGC-3′, *Islr*-WT reverse: 5′-GTCTGCAATCTGGAAGCCATACTTCTCC-3′;

Islr-CreERT2 forward: 5′-ACACACGACCTTGGCAAGTCCCAGC-3′, *Islr*-CreERT2 reverse: 5′-CGATCCCTGAACATGTCCATCAGG-3′;

Grem1-CreERT2 forward: 5′-TTAATCCATATTGGCAGAACGAAAACG-3′, *Grem1*-CreERT2 reverse: 5′-CAGGCTAAGTGCCTTCTCTACA-3′.

Rosa26-LSL-tdtomato forward: 5′-AGATCCACCAGGCCCTGAA-3′, Rosa26-LSL-tdtomato reverse: 5′-GTCTTGAACTCCACCAGGTAG-3′;

Rosa26-Cas9 forward: 5′-CGTCGTCCTTGAAGAAGATGGT-3′, Rosa26-Cas9 reverse: 5′-CACATGAAGCAGCACGACTT-3′; and

Rosa26-WT forward: 5′-TTCCCTCGTGATCTGCAACTC-3′, Rosa26-WT reverse: 5′-CTTTAAGCCTGCCCAGAAGACT-3′.

An Azoxymethane/Dextran Sulfate Sodium Mouse Model of Colorectal Cancer

A colitis-associated AOM/DSS mouse model of CRC was generated as described.[24] Briefly, AOM (Sigma, A5486; 10 mg/kg) was administered by intraperitoneal injection to C57BL/6J mice obtained from Charles River Laboratories Japan. One week later, the mice commenced the first of 3 cycles of DSS to induce inflammation (MP Biomedicals, 160110; molecular weight, 36,000–50,000 Da). Each cycle comprised 5 days of 3% DSS (weight/volume), then 16 days of normal drinking water. At 10 weeks after AOM injection, mice were killed, and colons were removed, opened longitudinally, and fixed in 10% formalin overnight at room temperature. Tissues were subsequently dehydrated and paraffin embedded, and 4-μm sections were used for histologic analysis.

Mouse Models of Colorectal Cancer Liver Metastasis

Tumoroids were isolated from Matrigel and dissociated to single cells by using TrypLE. The cells were filtered through 40-μm meshes to remove cell clumps and suspended in cold PBS containing 10 μmol/L Y-27632 (In Vitro Technologies); 5.0 $\times$ 10^5 cells in 100 μL were injected directly into the portal vein using a 33G syringe.

Male and female Rosa26-Cas9 mice (JAX stock no. 024858; C57BL/6 $\times$ 129 genetic background; 6 to 24 weeks old), housed under pathogen-free conditions in the SAHMRI Bioresources facility, were used for portal vein injection.

In all animal experiments, mice were allocated randomly to different treatment groups. Sample sizes were determined based on pilot experiments and sample availability.

Adeno-Associated Virus Packaging

AAV8 preparation was performed by the Vector and Genome Engineering Facility, Children's Medical Research Institute. The AAV vectors used in this study were packaged by using a standard transient transfection as described previously.[25] In short, pAAV transfer vector, pAd5 helper plasmid,[26] and AAV helper plasmid encoding rep2 and cap8 (pR2C8) were transfected by using PEI (Polysciences; catalogue no. 239662) into HEK293 cells. Assembled vector particles were purified by using iodixanol density gradients as described.[27] Vector genomes were quantified by using qRT-PCR as previously described.[28]

Intravenous Adeno-Associated Virus 8 Injection

Male and female Rosa26-Cas9 mice (JAX stock no. 024858, C57BL/6 $\times$ 129 genetic background, 6 to 24 weeks old) were administered intravenously via the tail vein, with a 150-μL injection of 1.0 $\times$ 10^{11} viral genomes of AAV8-mRuby2 or AAV8-*Islr* per mouse. A 2-week interval after AAV injection was allowed for protein expression to occur before portal vein injection of tumoroids. In Figure 6*D*, *E*, and *I*–*N*, AAV8-treated mice were harvested for histopathologic analyses 3–4 weeks after tumor injection, before prominent necrosis complicated histologic assessment.

Subcutaneous Injection of the GREM1-Neutralizing Antibody

Subcutaneous injection of the GREM1-neutralizing antibody or IgG isotype was commenced 1 week after tumoroid injection. A 30-mg/kg dose of the GREM1-neutralizing antibody (Ab7326) or IgG isotype (Ab101.4) was subcutaneously administered to each mouse twice a week until endpoint criteria were met.

In Vivo Imaging System

Liver metastasis tumor growth kinetics were assessed with an in vivo imaging system (IVIS) by using a Xenogen IVIS Spectrum Imaging System (PerkinElmer) 10 minutes after intraperitoneal injection of 150 mg/kg of D-luciferin

(L-8220, Biosynth Carbosynth). Luciferase activity was quantified by using Living Image software (PerkinElmer).

A Humane Endpoint in Animal Experiments

In survival analyses, to determine the humane endpoint in animal experiments, a clinical record score was used. Scores were obtained by giving 1 point for the presence of each of the following observations: weight loss of >15%, hunched/ruffled coat, dehydration, absence of movement, or facial grimace. Once a score of 3 was reached, the mice were killed.

Collagen Gel Contraction Assay

Collagen gels were prepared by mixing YH2 cells with PureCol bovine type I collagen (Advanced Biomatrix), 1× PBS, and 1 mol/L NaOH (final concentration: 1.0 mg/mL PureCol). The mixture containing 3.0×10^5 cells/mL was seeded in 24-well cell culture plates and allowed to polymerize for 20 minutes. DMEM supplemented with 10% FBS and 1% penicillin/streptomycin was added, and gels were released from the wells. The gel area was measured with ImageJ. Representative images were processed using Adobe Photoshop CC (Adobe).

Human Colorectal Samples

All ISH was performed by using surgery samples from patients with CRC who were diagnosed with adenocarcinoma of the colon or rectum at Nagoya University Hospital, Japan. This study was conducted in accordance with the Helsinki Declaration for Human Research and approved by the Ethics Committee of Nagoya University Graduate School of Medicine (approval no. 2017-0127).

Quantitative Image Analysis

IHC or ISH images were processed into separate channels representing nuclei staining (hematoxylin) and IHC or ISH staining (DAB) by using a color deconvolution function in Fiji software (ImageJ). Then, binary images were generated by intensity thresholds, and DAB^+ areas were calculated by ImageJ. To evaluate pSmad1/5/8 staining intensity, after an application of the lower threshold, the DAB staining intensity per high-power field was calculated by ImageJ.

In Figure 2*B*, (1) normal rectal mucosa adjacent to adenocarcinoma, (2) adenoma in carcinoma, and (3) adenocarcinoma were randomly selected from ISH samples and were used for quantification. Stromal areas (total areas excluding epithelial cells) visualized by hematoxylin counterstaining were outlined and measured by a clinical pathologist by using Adobe Photoshop CC (Adobe). In the normal mucosa, only lamina propria regions were quantified.

The Ki-67 labeling index was calculated as the percentage of Ki-67^+ cells in the total epithelial cells as visualized by hematoxylin counterstaining.

The MRI fibrosis tool (https://github.com/MontpellierRessourcesImagerie/imagej_macros_and_scripts/wiki/MRI_Fibrosis_Tool) was used to quantify the percentage of fibrosis areas (Picro-Sirius red^+ areas) at default settings.

Distance Analysis Using In Situ Hybridization Samples

In Figure 4*H* and *I*, serial sections of human desmoplastic rectal cancer samples, in which the stroma accounted for more than 40% of the total tumor area, were evaluated. The minimum edge-to-edge distance between ISH signals and the closest tumor glands was calculated by the DiAna plugin[29] for ImageJ. Briefly, tumor glands in ISH images were outlined by a clinical pathologist. Then, to identify ISH signals (DAB^+ signals), the ISH images were subjected to color deconvolution before application of intensity thresholding and a watershed algorithm in Fiji software. ISH signals were segmented by using a minimum size threshold by the DiAna plugin. Then, the smallest edge-to-edge distance between each ISH signal and the closest tumor gland was computed by the DiAna plugin.

Evaluation of Tumor Differentiation and Tumor Budding

Evaluation of tumor differentiation and tumor budding was performed by a clinical pathologist. Poorly differentiated adenocarcinomas were defined as cancers that exhibit glandular differentiation in 5%–50% of the tumor, as described.[30]

Tumor budding was defined as a single tumor cell or a cell cluster of up to 4 tumor cells, as described.[31]

Data and Materials Availability

Data that support the findings of this study are available from the corresponding author upon reasonable request. Noncommercially available materials described in this study may be obtained with a material transfer agreement. Requests for materials should be addressed to Susan L. Woods.

Supplementary References

1. Fornes O, Castro-Mondragon JA, Khan A, et al. JASPAR 2020: update of the open-access database of transcription factor binding profiles. Nucleic Acids Res 2020;48(D1):D87–D92.
2. Mizutani Y, Kobayashi H, Iida T, et al. Meflin-positive cancer-associated fibroblasts inhibit pancreatic carcinogenesis. Cancer Res 2019;79:5367–5381.
3. Maeda K, Enomoto A, Hara A, et al. Identification of meflin as a potential marker for mesenchymal stromal cells. Sci Rep 2016;6:22288.
4. Persons DA, Mehaffey MG, Kaleko M, et al. An improved method for generating retroviral producer clones for vectors lacking a selectable marker gene. Blood Cells Mol Dis 1998;24:167–182.
5. Hirokawa Y, Yip KH, Tan CW, et al. Colonic myofibroblast cell line stimulates colonoid formation. Am J Physiol Gastrointest Liver Physiol 2014;306:G547–G556.
6. Khalil H, Nie W, Edwards RA, et al. Isolation of primary myofibroblasts from mouse and human colon tissue. J Vis Exp 2013;80:e50611.

7. Ran FA, Hsu PD, Wright J, et al. Genome engineering using the CRISPR-Cas9 system. Nat Protoc 2013;8:2281–2308.
8. Platt RJ, Chen S, Zhou Y, et al. CRISPR-Cas9 knockin mice for genome editing and cancer modeling. Cell 2014;159:440–455.
9. Doench JG, Fusi N, Sullender M, et al. Optimized sgRNA design to maximize activity and minimize off-target effects of CRISPR-Cas9. Nat Biotechnol 2016;34:184–191.
10. Ponomarev V, Doubrovin M, Serganova I, et al. A novel triple-modality reporter gene for whole-body fluorescent, bioluminescent, and nuclear noninvasive imaging. Eur J Nucl Med Mol Imaging 2004;31:740–751.
11. Aubrey BJ, Kelly GL, Kueh AJ, et al. An inducible lentiviral guide RNA platform enables the identification of tumor-essential genes and tumor-promoting mutations in vivo. Cell Rep 2015;10:1422–1432.
12. Schneider CA, Rasband WS, Eliceiri KW. NIH Image to ImageJ: 25 years of image analysis. Nat Methods 2012;9:671–675.
13. Ye J, Coulouris G, Zaretskaya I, et al. Primer-BLAST: a tool to design target-specific primers for polymerase chain reaction. BMC Bioinformatics 2012;13:134.
14. Calon A, Espinet E, Palomo-Ponce S, et al. Dependency of colorectal cancer on a TGF-β-driven program in stromal cells for metastasis initiation. Cancer Cell 2012;22:571–584.
15. Phipson B, Lee S, Majewski IJ, et al. Robust hyperparameter estimation protects against hypervariable genes and improves power to detect differential expression. Ann Appl Stat 2016;10:946–963.
16. Sheffer M, Bacolod MD, Zuk O, et al. Association of survival and disease progression with chromosomal instability: a genomic exploration of colorectal cancer. Proc Natl Acad Sci U S A 2009;106:7131–7136.
17. Marisa L, de Reynies A, Duval A, et al. Gene expression classification of colon cancer into molecular subtypes: characterization, validation, and prognostic value. PLoS Med 2013;10:e1001453.
18. Smith JJ, Deane NG, Wu F, et al. Experimentally derived metastasis gene expression profile predicts recurrence and death in patients with colon cancer. Gastroenterology 2010;138:958–968.
19. Camp RL, Dolled-Filhart M, Rimm DL. X-tile: a new bioinformatics tool for biomarker assessment and outcome-based cut-point optimization. Clin Cancer Res 2004;10:7252–7259.
20. Guinney J, Dienstmann R, Wang X, et al. The consensus molecular subtypes of colorectal cancer. Nat Med 2015;21:1350–1356.
21. The Cancer Genome Atlas Network. Comprehensive molecular characterization of human colon and rectal cancer. Nature 2012;487(7407):330–337.
22. Lee HO, Hong Y, Etlioglu HE, et al. Lineage-dependent gene expression programs influence the immune landscape of colorectal cancer. Nat Genet 2020;52:594–603.
23. Li H, Courtois ET, Sengupta D, et al. Reference component analysis of single-cell transcriptomes elucidates cellular heterogeneity in human colorectal tumors. Nat Genet 2017;49:708–718.
24. Neufert C, Becker C, Neurath MF. An inducible mouse model of colon carcinogenesis for the analysis of sporadic and inflammation-driven tumor progression. Nat Protoc 2007;2:1998–2004.
25. Xiao X, Li J, Samulski RJ. Production of high-titer recombinant adeno-associated virus vectors in the absence of helper adenovirus. J Virol 1998;72:2224–2232.
26. Parmiani G. Immunological approach to gene therapy of human cancer: improvements through the understanding of mechanism(s). Gene Ther 1998;5:863–864.
27. Strobel B, Miller FD, Rist W, et al. Comparative analysis of cesium chloride- and iodixanol-based purification of recombinant adeno-associated viral vectors for preclinical applications. Hum Gene Ther Methods 2015;26:147–157.
28. Wang Q, Dong B, Firrman J, et al. Efficient production of dual recombinant adeno-associated viral vectors for factor VIII delivery. Hum Gene Ther Methods 2014;25:261–268.
29. Gilles J-F, Dos Santos M, Boudier T, et al. DiAna, an ImageJ tool for object-based 3D co-localization and distance analysis. Methods 2017;115:55–64.
30. Jackstadt R, van Hooff SR, Leach JD, et al. Epithelial NOTCH signaling rewires the tumor microenvironment of colorectal cancer to drive poor-prognosis subtypes and metastasis. Cancer Cell 2019;36:319–336.
31. Lugli A, Kirsch R, Ajioka Y, et al. Recommendations for reporting tumor budding in colorectal cancer based on the International Tumor Budding Consensus Conference (ITBCC) 2016. Mod Pathol 2017;30:1299–1311.
32. Fleming NI, Jorissen RN, Mouradov D, et al. *SMAD2, SMAD3* and *SMAD4* mutations in colorectal cancer. Cancer Res 2013;73:725–735.

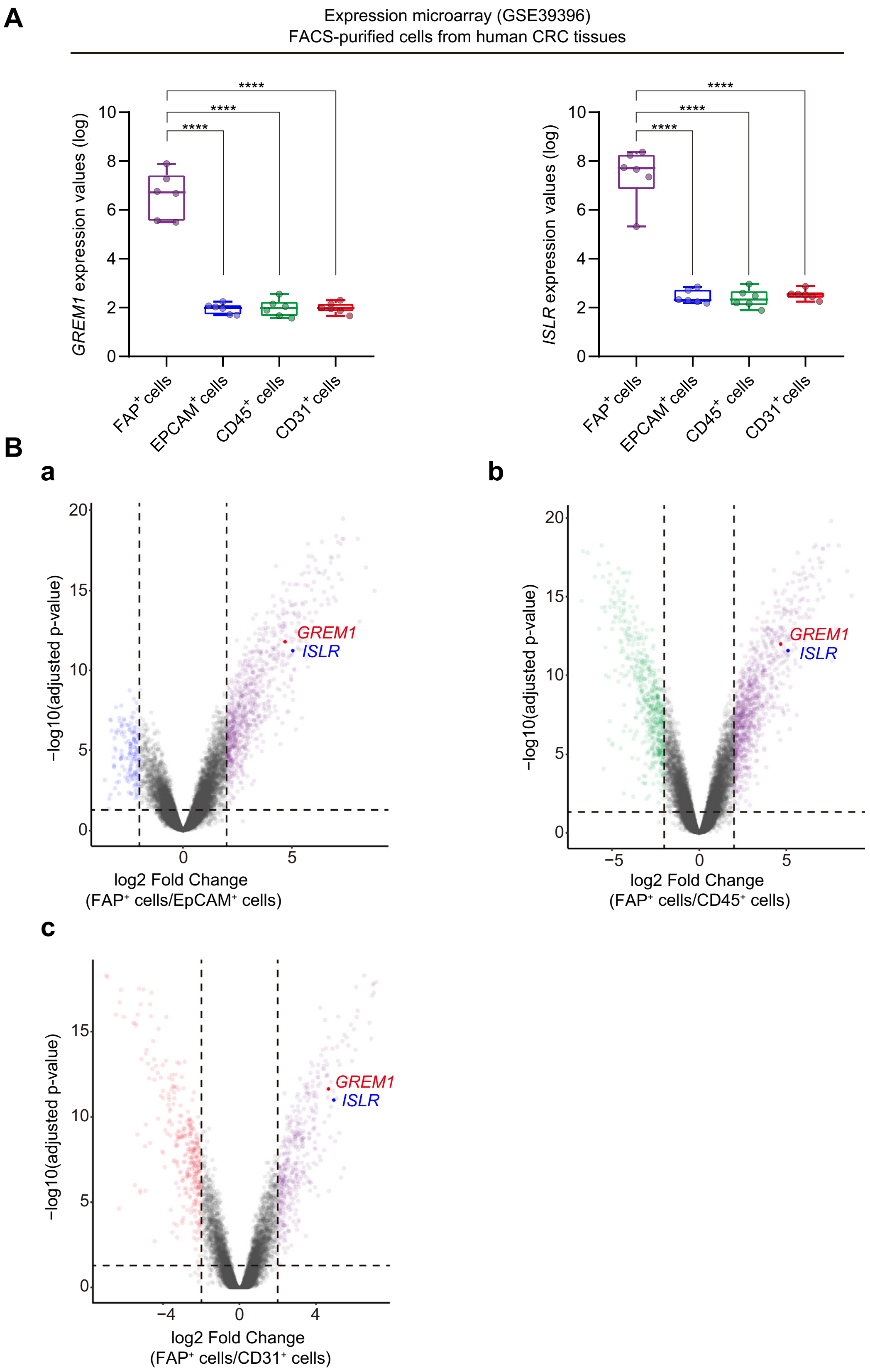
A
Expression microarray (GSE39396)
FACS-purified cells from human CRC tissues
GREM1 expression values (log)
ISLR expression values (log)
****
FAP+ cells
EPCAM+ cells
CD45+ cells
CD31+ cells
B
a
b
c
−log10(adjusted p-value)
GREM1
ISLR
log2 Fold Change
(FAP+ cells/EpCAM+ cells)
(FAP+ cells/CD45+ cells)
(FAP+ cells/CD31+ cells)

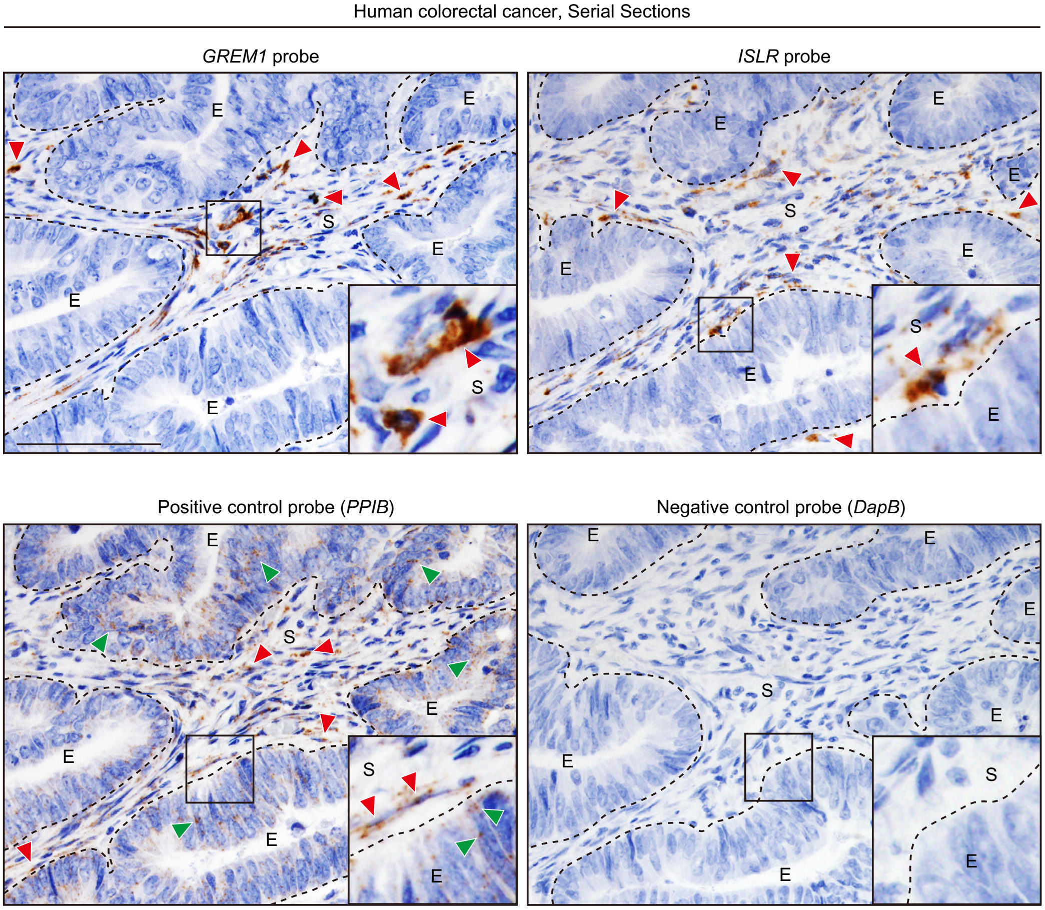

Supplementary Figure 2. Validation of ISH staining using positive and negative control probes. Representative ISH pictures using serial sections from human colorectal cancer. ISH for *GREM1* and *ISLR* shows 3,3′-DAB^{+} staining specifically in the stroma, whereas positive control probe (*PPIB*, a ubiquitously expressed gene) staining is observed both in the epithelial cells and stroma. No apparent background staining is seen in ISH for a negative control probe that targets the bacterial *DapB* gene. Dotted lines indicate the borders between epithelial cells (E) and the stroma (S). Red and green arrowheads denote DAB^{+} staining in the stroma and epithelial cells, respectively. The boxed areas are magnified in the insets. Scale bar, 100 μm.

Supplementary Figure 1. Analyses of a human CRC expression microarray data set reveal that *GREM1* and *ISLR* are highly expressed in FAP^{+} CAFs compared to EpCAM^{+} epithelial cells, CD45^{+} immune cells, and CD31^{+} endothelial cells. (*A*) *GREM1* and *ISLR* expression levels in a cDNA expression microarray from FACS-purified cells from human primary CRC tissues (GSE39396); n = 6 patients each. One-way ANOVA followed by Tukey post hoc multiple comparisons. ****$P < .0001$. Box plots have whiskers of maximum and minimum values; the boxes represent first, second (median), and third quartiles. (*B*) Volcano plots showing differentially expressed transcripts between FAP^{+} CAFs and EpCAM^{+} epithelial cells (*a*), between FAP^{+} CAFs and CD45^{+} immune cells (*b*), and between FAP^{+}CAFs and CD31^{+} endothelial cells (*c*) (GSE39396); n = 6 patients each. The adjusted *P* value cutoffs and the $\log_2$ fold change cutoffs for differentially expressed genes were 0.05 and 2, respectively. Semitransparent purple, blue, green, and red dots denote differentially expressed transcripts, which are up-regulated in FAP^{+} CAFs, EpCAM^{+} epithelial cells, CD45^{+} immune cells, and CD31^{+} endothelial cells, respectively. Gray dots represent transcripts that are not differentially expressed. *GREM1* and *ISLR* gene probes used for the analyses are as follows: 218468_PM_s_at (*GREM1*) and 207191_PM_s_at (*ISLR*).

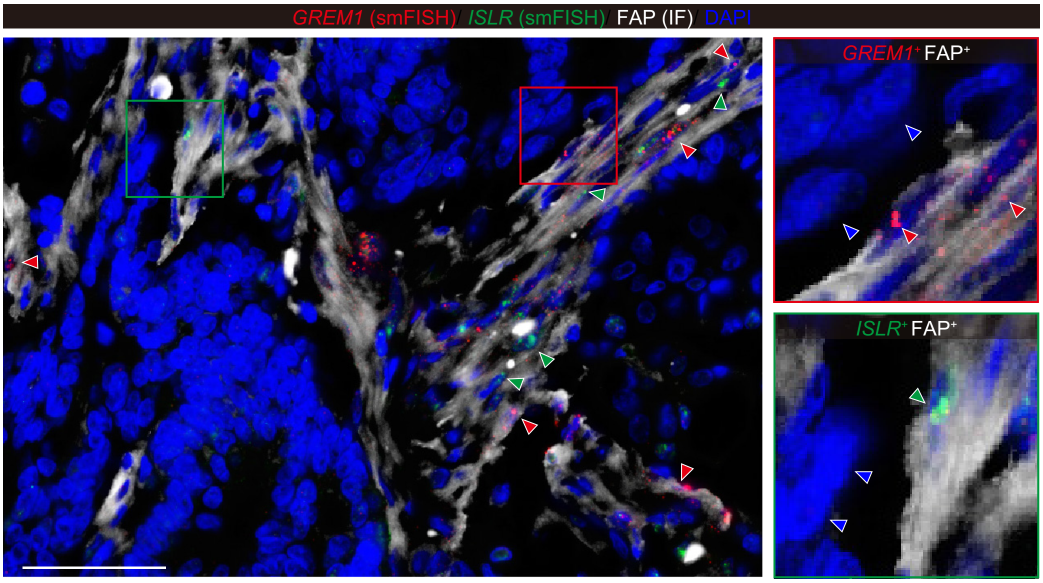

Supplementary Figure 3. *GREM1* and *ISLR* transcripts are detected in FAP$^+$ CAFs in single-molecule FISH on human CRC sections. Representative picture of *GREM1* and *ISLR* dual smFISH followed by FAP IF, using human CRC tissue sections. Red and green arrowheads denote *GREM1*$^+$FAP$^+$ cells and *ISLR*$^+$FAP$^+$ cells, respectively. Blue arrowheads indicate FAP$^-$ cells that do not express *GREM1* or *ISLR*. N = 3 patients. Scale bar, 50 μm.

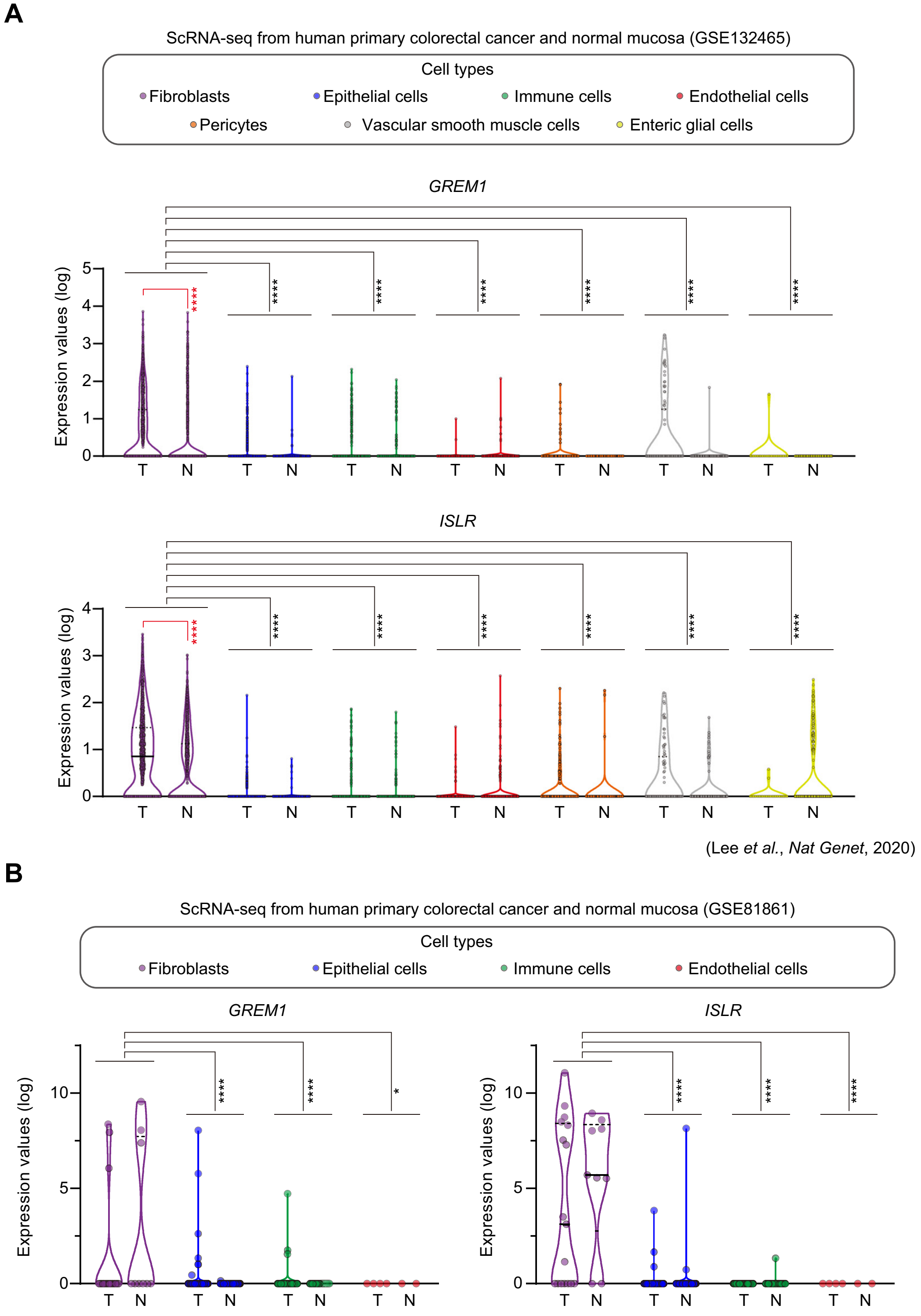

Supplementary Figure 4. *GREM1* and *ISLR* are highly expressed in fibroblasts in single-cell RNA-seq data from human normal colorectal mucosa and primary CRC. (*A*) Violin plots depicting *GREM1* and *ISLR* expression levels in scRNA-seq of unsorted cells from human primary CRC tissues and normal colorectal tissues (GSE132465).[22] In fibroblasts, *GREM1* and *ISLR* transcripts are differentially up-regulated in tumors compared with normal samples (Wilcoxon tests, red asterisks); n = 1501; 1961; 17,469; 1070; 27,080; 12,137; 768; 739; 353; 92; 91; 123; 23; and 282 cells (*left to right*). Tumor samples from 23 patients. Normal samples from 10 of the patients. (*B*) Violin plots showing *GREM1* and *ISLR* expression levels in scRNA-seq of unsorted cells from human primary CRC tissues and normal colorectal tissues (GSE81861)[23]; n = 17, 9, 272, 160, 71, 42, 4, and 2 cells (*left to right*). Tumor samples from 11 patients. Normal samples from 7 of the patients. Kruskal-Wallis tests followed by Dunn post hoc multiple comparisons were used to compare *GREM1* or *ISLR* expression levels in fibroblasts from tumor and normal samples with those in other cell populations (*black asterisks*). ****$P < .0001$, *$P = .0132$. Solid black lines, median; dotted black lines, quartiles. N, normal samples; T, tumor samples.

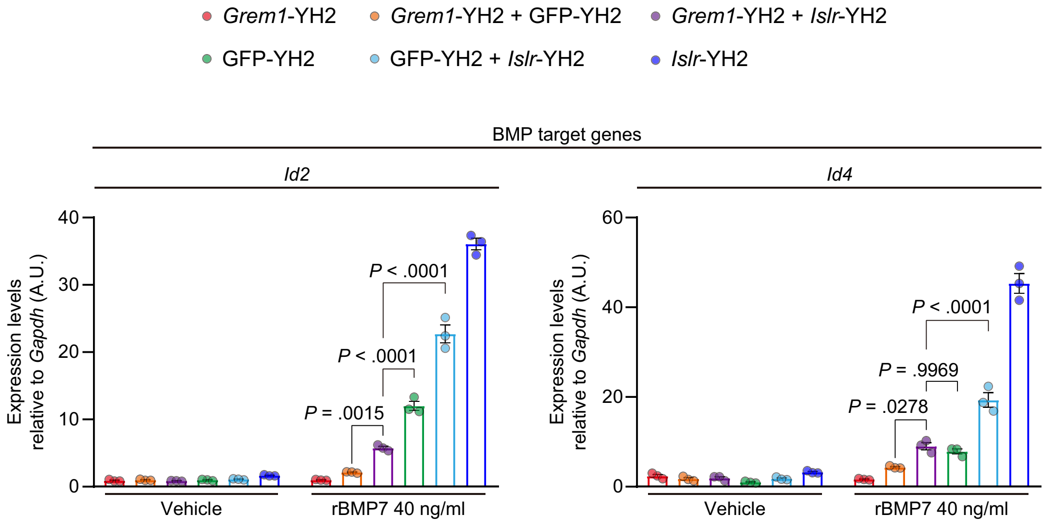

Supplementary Figure 5. GREM1 and ISLR overexpression counteract each other's effects on BMP7-*Id2/4* signaling in vitro. qRT-PCR for BMP target genes *Id2* and *Id4*. YH2 cells that overexpress GREM1, GFP, or ISLR were admixed at a 1:1 ratio and stimulated with 40 ng/mL of recombinant BMP7 for 24 hours (*orange, purple, and aqua*). *Grem1*-YH2 cells, GFP-YH2 cells, or *Islr*-YH2 cells alone were used as controls (*red, green, and blue, respectively*). The same number of cells (a total of 5.0×10^4 cells/well) were seeded in a 6-well plate in each condition. n = 3 each; mean ± SEM. Two-way ANOVA with post hoc Tukey multiple comparisons. A.U., arbitrary unit.

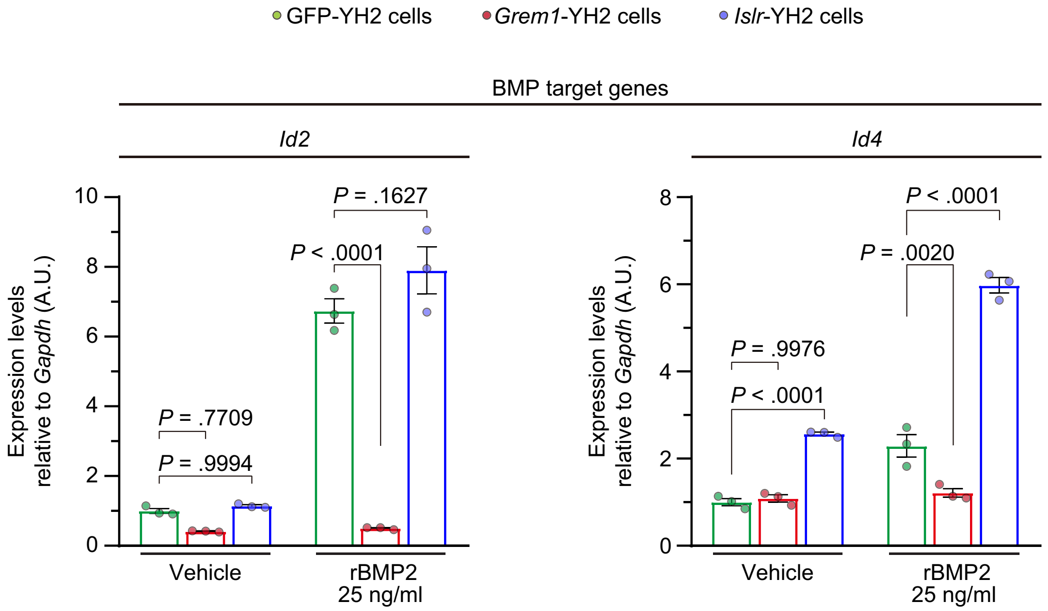

Supplementary Figure 6. BMP2-induced increase in *Id4* is prevented by GREM1 and augmented by ISLR in YH2 cells. qRT-PCR for *Id2* and *Id4* in YH2 cells. GFP-, *Grem1*-, or *Islr*-overexpressing YH2 cells were treated with 25 ng/mL of recombinant BMP2 for 24 hours. Note that GREM1 prevented the BMP2-induced increase in *Id2*, whereas ISLR did not significantly promote the BMP2-mediated increase in *Id2* in YH2 cells. n = 3 each. Mean ± SEM. Two-way ANOVA with post hoc Tukey multiple comparisons.

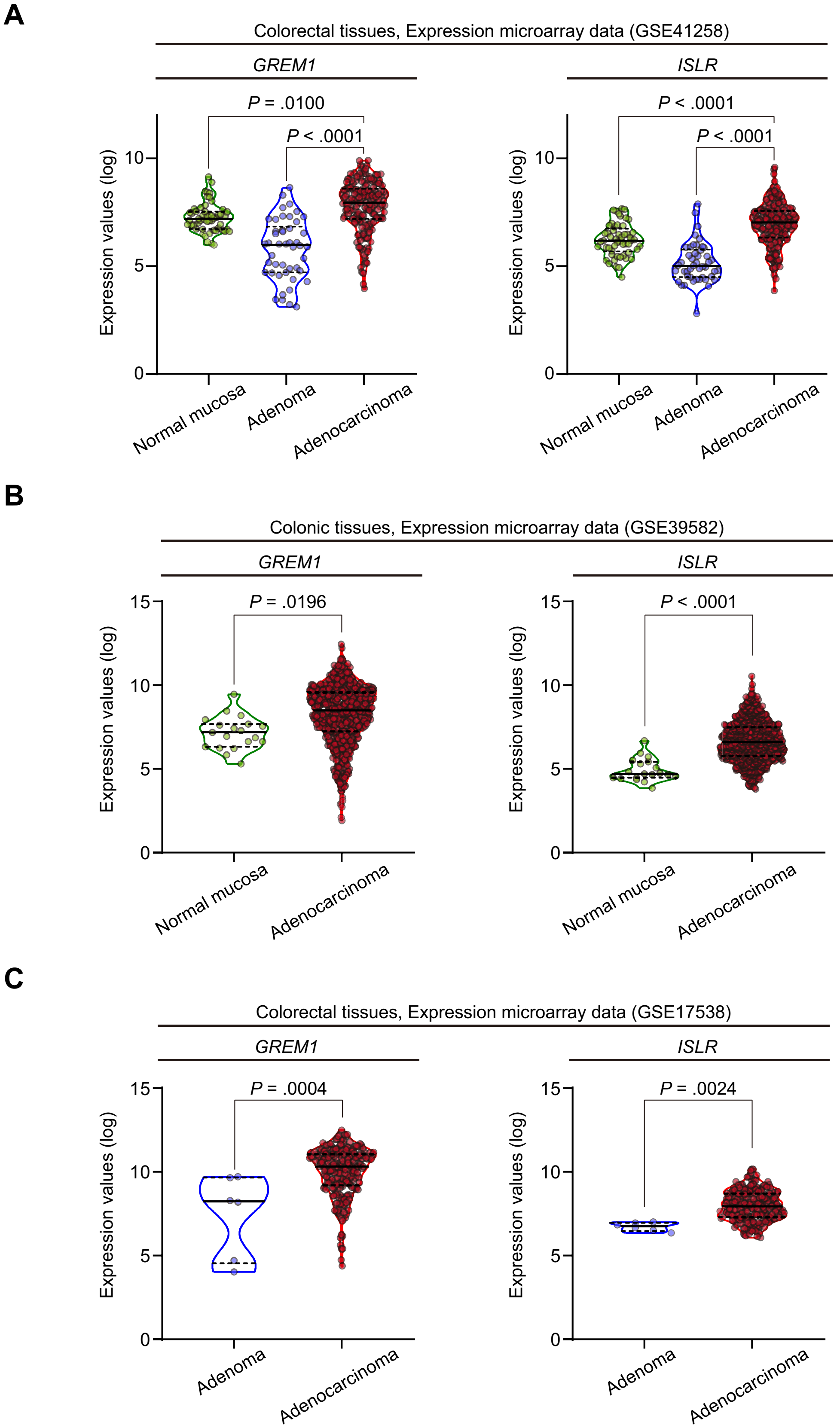

Supplementary Figure 7. *GREM1* and *ISLR* expression levels are up-regulated during human CRC carcinogenesis. (*A*) Violin plots depicting *GREM1* and *ISLR* expression in cDNA expression microarray data from human normal colorectal mucosa, colorectal adenoma, and primary colorectal adenocarcinoma (GSE41258).[16] n = 53 (normal mucosa), 46 (adenoma), and 182 patients (adenocarcinoma). (*B*) Violin plots depicting *GREM1* and *ISLR* expression in cDNA expression microarray data from human normal colon mucosa and primary colon adenocarcinoma (GSE39582).[17] n = 19 (normal colon mucosa) and 566 patients (primary colon adenocarcinoma). (*C*) Violin plots depicting *GREM1* and *ISLR* expression in cDNA expression microarray data from human colorectal adenoma and primary colorectal adenocarcinoma (GSE17538).[18] n = 6 (colorectal adenoma) and 232 patients (primary colorectal adenocarcinoma). One-way ANOVA followed by Tukey post hoc multiple comparisons (*A*). FDR-adjusted *P*- values are shown (*B* and *C*). Solid black lines, median; dotted black lines, quartiles.

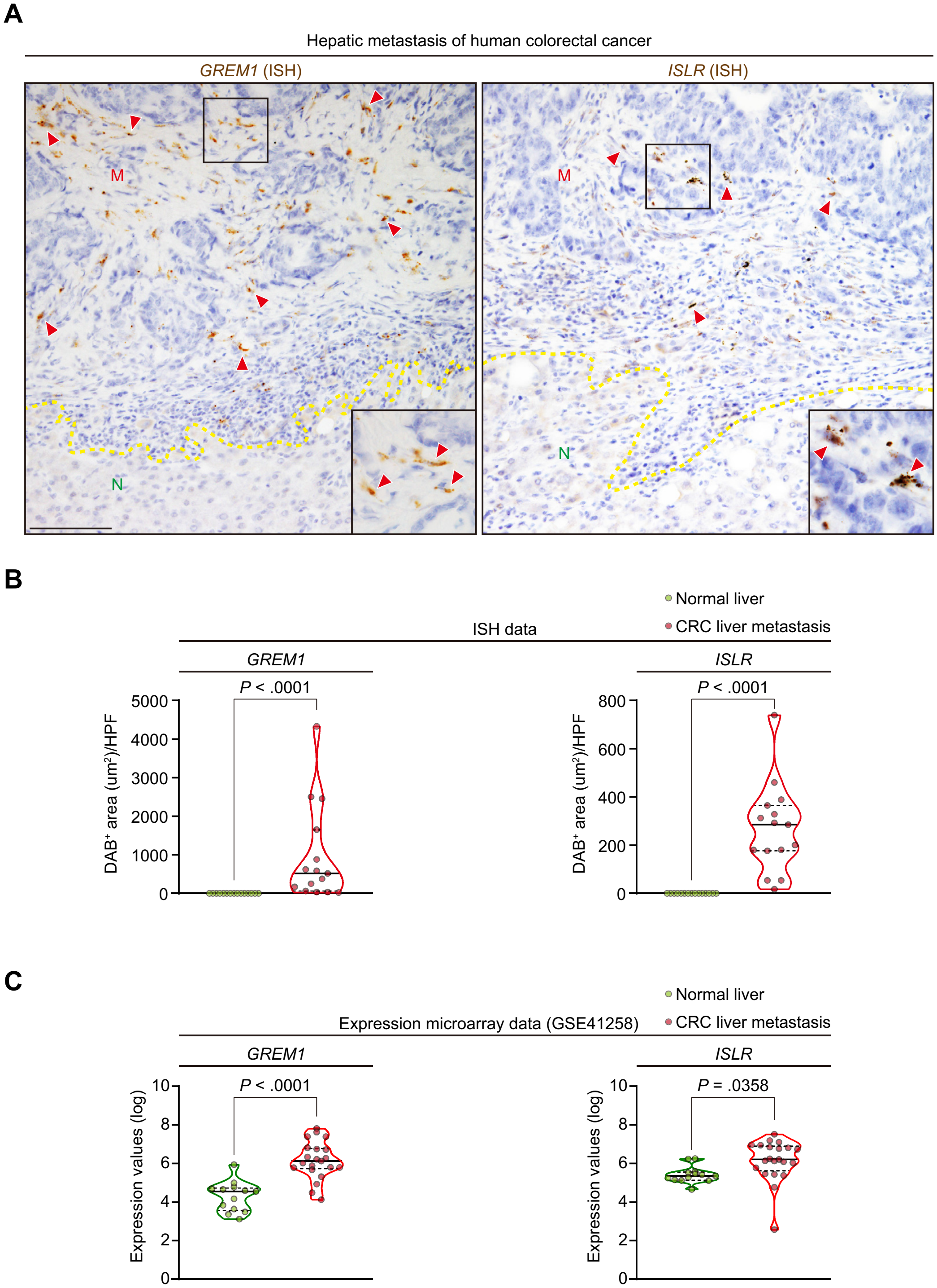

Supplementary Figure 8. *GREM1* and *ISLR* are expressed in the stroma of human CRC hepatic metastases. (*A*, *B*) ISH for *GREM1* and *ISLR* in the hepatic metastases of human CRC. (*A*) Representative images. *GREM1* and *ISLR* are expressed by fibroblastic cells in the stroma of the liver metastases. Dotted yellow lines indicate the borders between the normal liver (N) and CRC liver metastases (M). Red arrowheads denote *GREM1* or *ISLR* expression. The boxed areas are magnified in the insets. Scale bars, 100 μm. (*B*) Violin plots showing quantification of 3,3′-DAB^{+} areas by ImageJ; 5 HPFs/patient, 3 patients each. Solid black lines, median; dotted black lines, quartiles. (*C*) Violin plots depicting *GREM1* and *ISLR* expression in cDNA expression microarray data from human normal liver and liver metastasis of CRC (GSE41258). n = 13 (normal liver) and 21 patients (CRC liver metastasis). Solid black lines, median; dotted black lines, quartiles. Mann-Whitney *U* test (B). FDR-adjusted *P* values are shown in (*C*). HPF, high-power field.

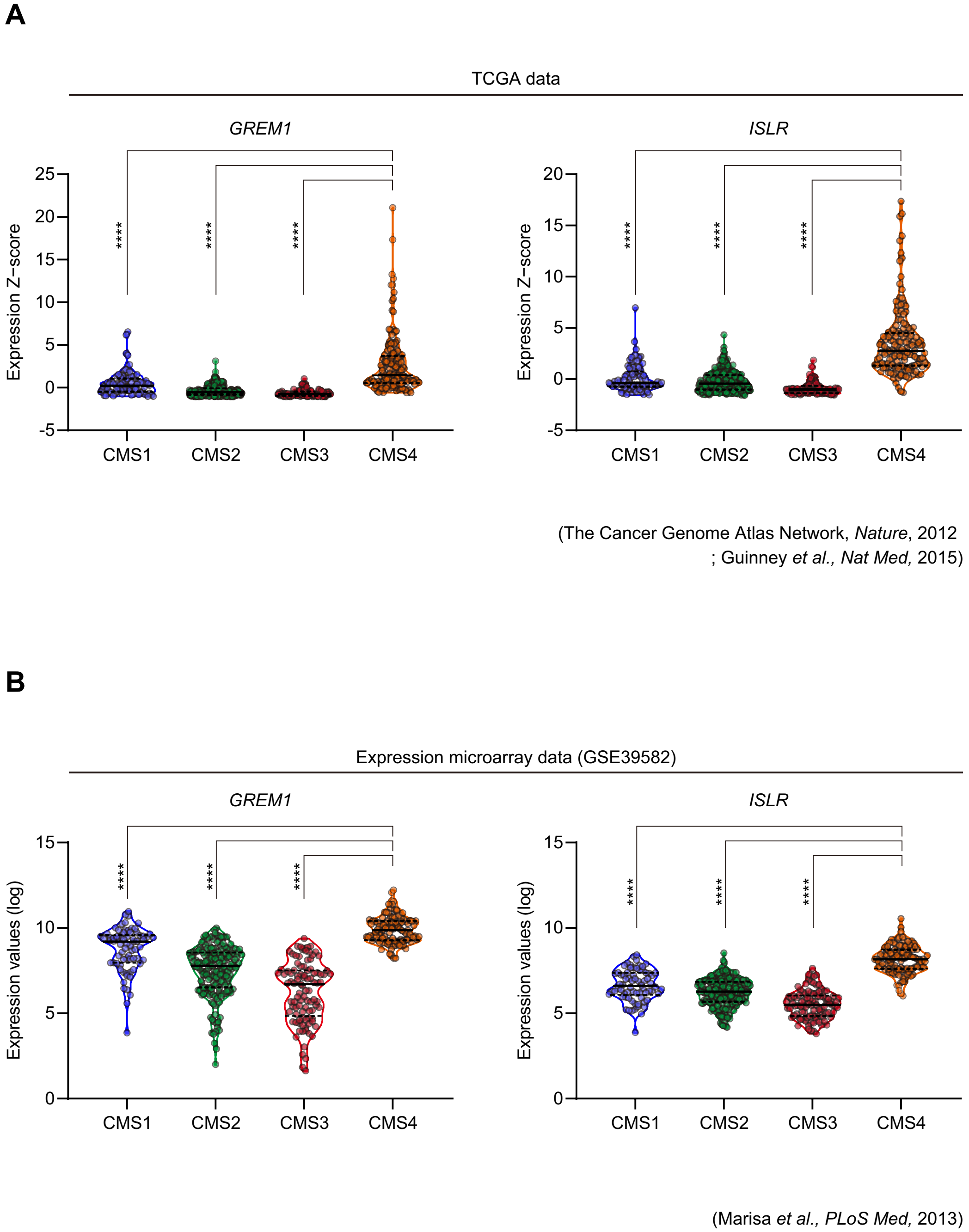

Supplementary Figure 9. *GREM1* and *ISLR* are highly expressed in CMS 4 CRC. (*A*) Violin plots showing expression levels of *GREM1* and *ISLR* in 4 CMSs.[20] Primary colon and rectal adenocarcinoma samples in TCGA data were analyzed; n = 76 (CMS1), 220 (CMS2), 72 (CMS3), and 143 patients (CMS4). (*B*) Violin plots depicting *GREM1* and *ISLR* expression levels in 4 CMSs. Primary colon adenocarcinoma samples were analyzed by using cDNA expression microarray data (GSE39582).[17] n = 70 (CMS1), 177 (CMS2), 97 (CMS3), and 107 patients (CMS4). Kruskal-Wallis test followed by Dunn post hoc multiple comparisons. Solid black lines, median; dotted black lines, quartiles. ****$P < .0001$. TCGA, The Cancer Genome Atlas.

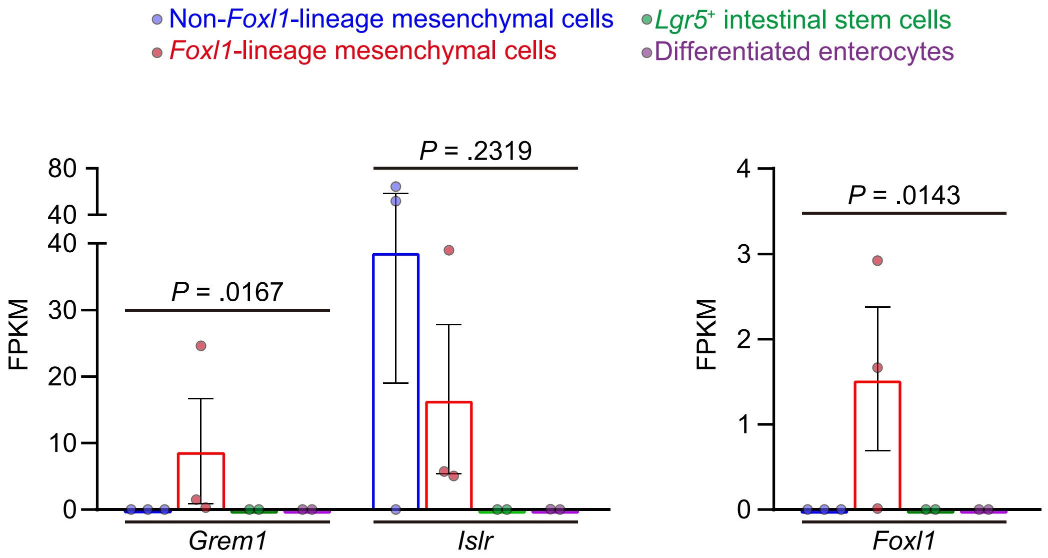

Supplementary Figure 10. *Grem1* is highly expressed in *Foxl1*-lineage telocytes in the mouse small intestine. Expression levels of *Grem1*, *Islr*, and *Foxl1* in RNA-seq data from FACS-purified non–*Foxl1*-lineage mesenchymal cells, *Foxl1*-lineage mesenchymal cells (sorted from *Fox1*-Cre;Rosa26-YFP or *Foxl1*-Cre;Rosa26-mT/mG), *Lgr5*$^{+}$ intestinal stem cells (sorted by *Lgr5*-eGFP^{high} expression), and differentiated enterocytes (GSE94072). Note that FPKM values for *Grem1* and *Foxl1* were 0 in all 3 biological replicates of non-*Foxl1* lineage mesenchymal cells. n = 3 (non–*Foxl1*-lineage mesenchymal cells), 3 (*Foxl1*-lineage mesenchymal cells), 2 (*Lgr5*$^{+}$ intestinal stem cells), and 2 (differentiated enterocytes) mice. All data are represented as mean ± SEM. Statistical analyses were performed using the Kruskal-Wallis test.

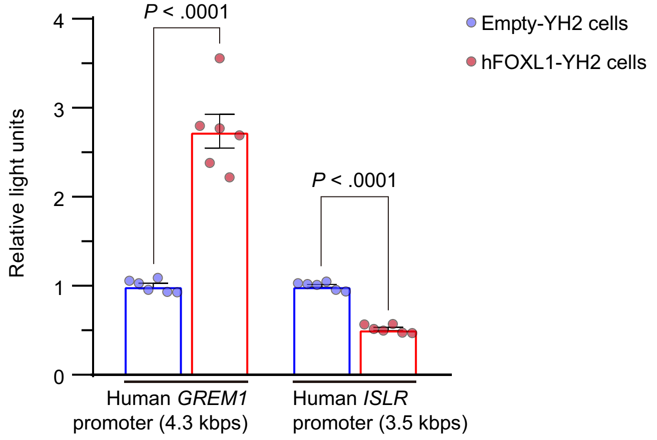

Supplementary Figure 11. Lentivirus-mediated FOXL1 overexpression increases *GREM1*-promoter activity and decreases *ISLR*-promoter activity. Luciferase assays of human *GREM1*- or *ISLR*-promoter regions using human FOXL1-overexpressing YH2 cells and control empty YH2 cells. n = 6 each. Data are represented as mean ± SEM. Statistical analysis was performed by using the 2-tailed unpaired Student *t* test.

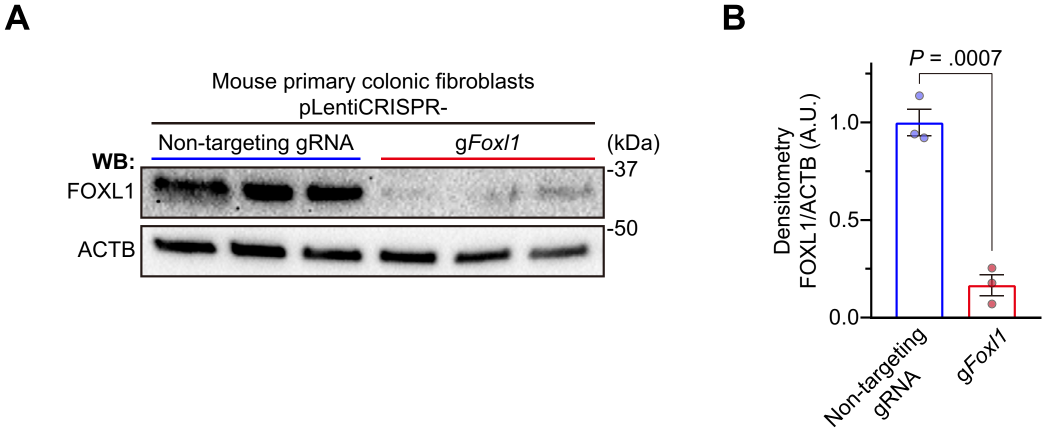

Supplementary Figure 12. Validation of CRISPR/Cas9–mediated FOXL1 knockdown in mouse primary colonic fibroblasts by WB. (*A*) WB showing FOXL1 knockdown by lentiviral transduction. (*B*) Densitometry of the WB in *A* showing decreased FOXL1 protein expression by lentiviral transduction. n = 3 mice each. Data are represented as mean ± SEM. Statistical analysis was performed by using the 2-tailed unpaired Student *t* test.

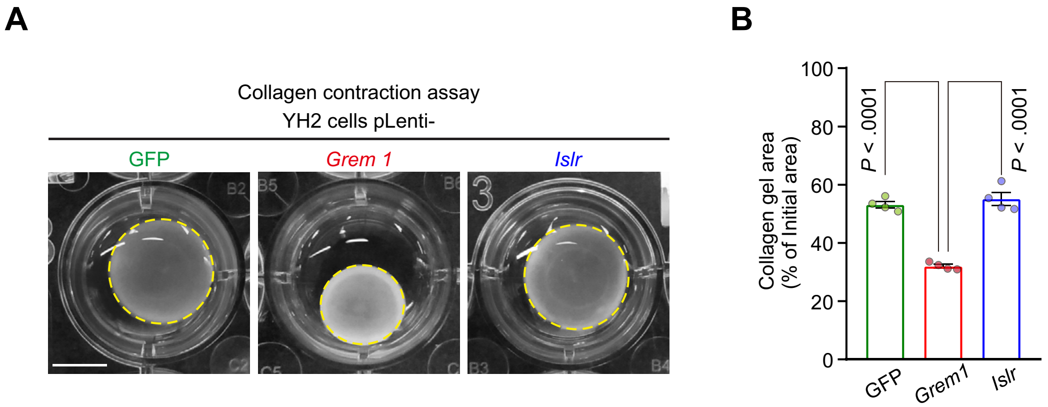

Supplementary Figure 13. Increased collagen gel contraction in *Grem1*-overexpressing YH2 cells. (*A*, *B*) Collagen gel contraction assay using GFP-, *Grem1*- and *Islr*-overexpressing YH2 cells. (*A*) Representative pictures. Collagen gel areas are outlined by dotted yellow lines. Scale bars, 5 mm. (*B*) Quantification of collagen gel areas. Collagen gel areas were evaluated 12 hours after seeding cells; n = 4 each. Data are represented as mean ± SEM. Statistical analysis was performed by using 1-way ANOVA with Tukey post hoc multiple comparisons.

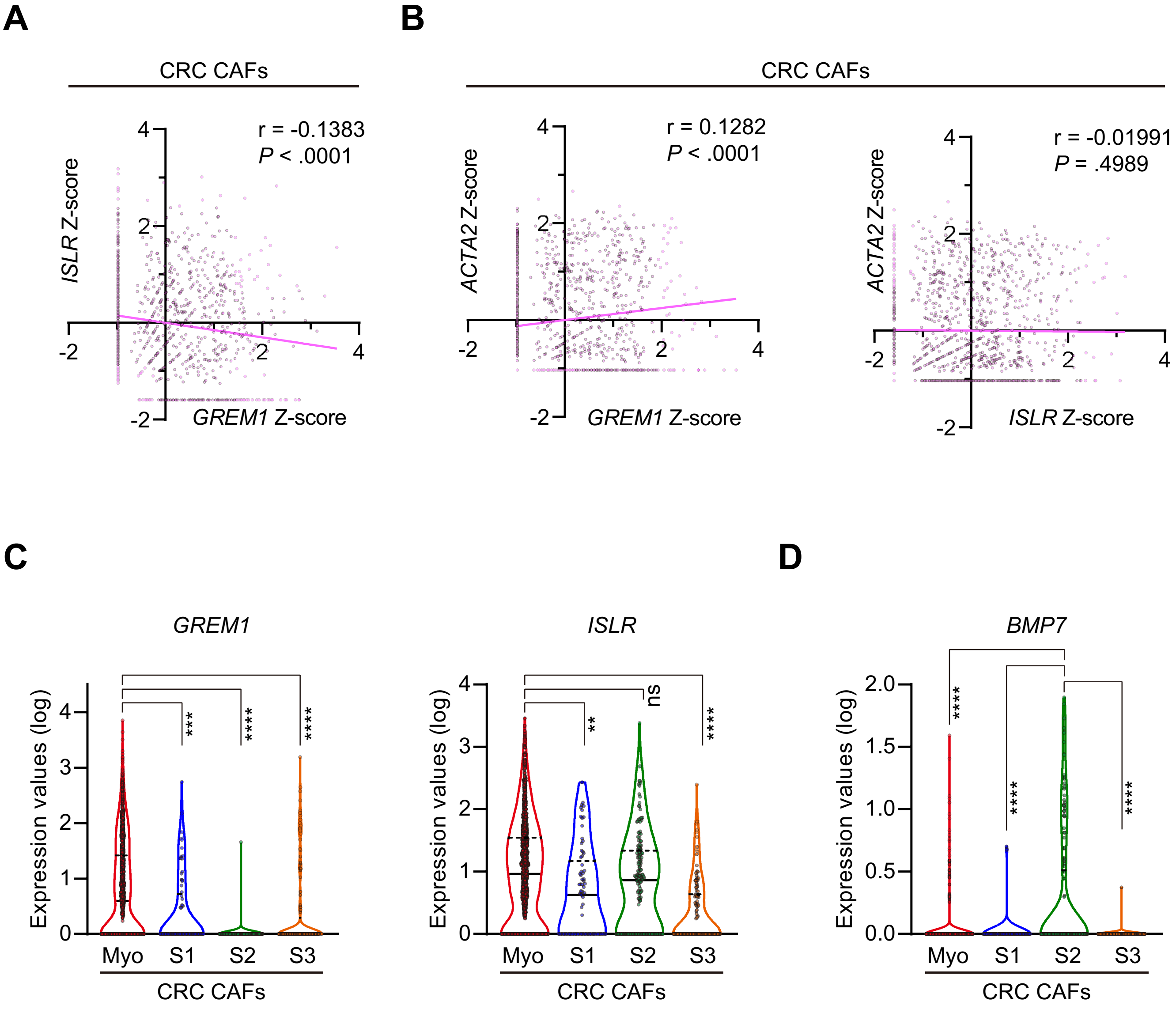

Supplementary Figure 14. ScRNA-seq from human CRC tissues shows that *GREM1* identifies a myofibroblastic CAF subpopulation that is distinct from *ISLR*$^{+}$ CAFs. (*A*, *B*) Spearman correlation analyses of gene expression levels in scRNA-seq data from human CRC tissues (GSE132465).[22] (*A*) *GREM1* transcript levels are inversely correlated with *ISLR* expression in human CRC CAFs. (*B*) *GREM1*, but not *ISLR*, transcripts are positively correlated with *ACTA2* transcripts. In a total of 1501 CAFs, 1156 human CRC CAFs that express *GREM1* or *ISLR* transcripts were analyzed. Spearman *r* and *P* values are reported in the figure. Solid pink line, linear regression. (*C*, *D*) Violin plots depicting *GREM1* and *ISLR* expression (*C*) and BMP7 expression (*D*) in 4 different CRC CAF subclusters[22]; n = 1146 (myofibroblasts), 73 (S1 fibroblasts), 158 (S2 fibroblasts), and 124 cells (S3 fibroblasts). Kruskal-Wallis test followed by Dunn post hoc multiple comparisons. ****$P < .0001$; ***$P = .0005$; **$P = .0098$; ns, $P = .1375$. Solid black lines, median; dotted black lines, quartiles. Myo, myofibroblasts; S1, stromal 1 fibroblasts; S2, stromal 2 fibroblasts; S3, stromal 3 fibroblasts.

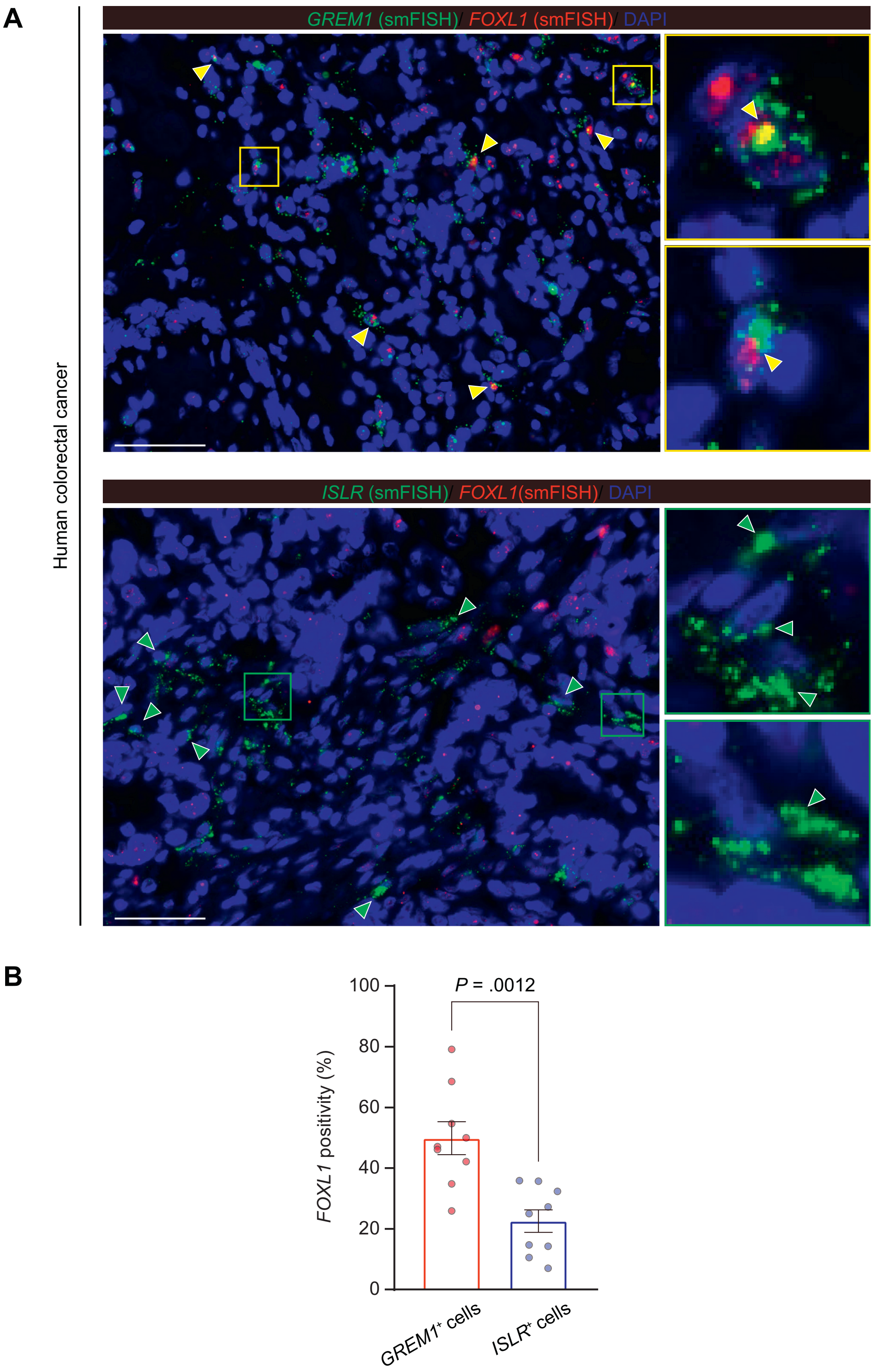

Supplementary Figure 15. *GREM1*$^{+}$ CAFs shows higher positivity for *FOXL1* in human CRC than *ISLR*$^{+}$ CAFs. (*A*, *B*) Dual smFISH for *GREM1*/*FOXL1* (*upper panel in A*) and *ISLR/FOXL1* (*lower panel in A*) in human CRC samples. (*A*) Representative pictures. Yellow and green arrowheads denote *GREM1*$^{+}$*FOXL1*$^{+}$ cells and *ISLR*$^{+}$*FOXL1*$^{-}$ cells, respectively. The boxed areas are magnified in the adjacent panels. (*B*) Semiquantification of *FOXL1* positivity in *GREM1*$^{+}$ cells and *ISLR*$^{+}$ cells. Three HPFs/patient, 3 patients. Mean ± SEM. Mann-Whitney *U* test. Scale bars, 50 μm.

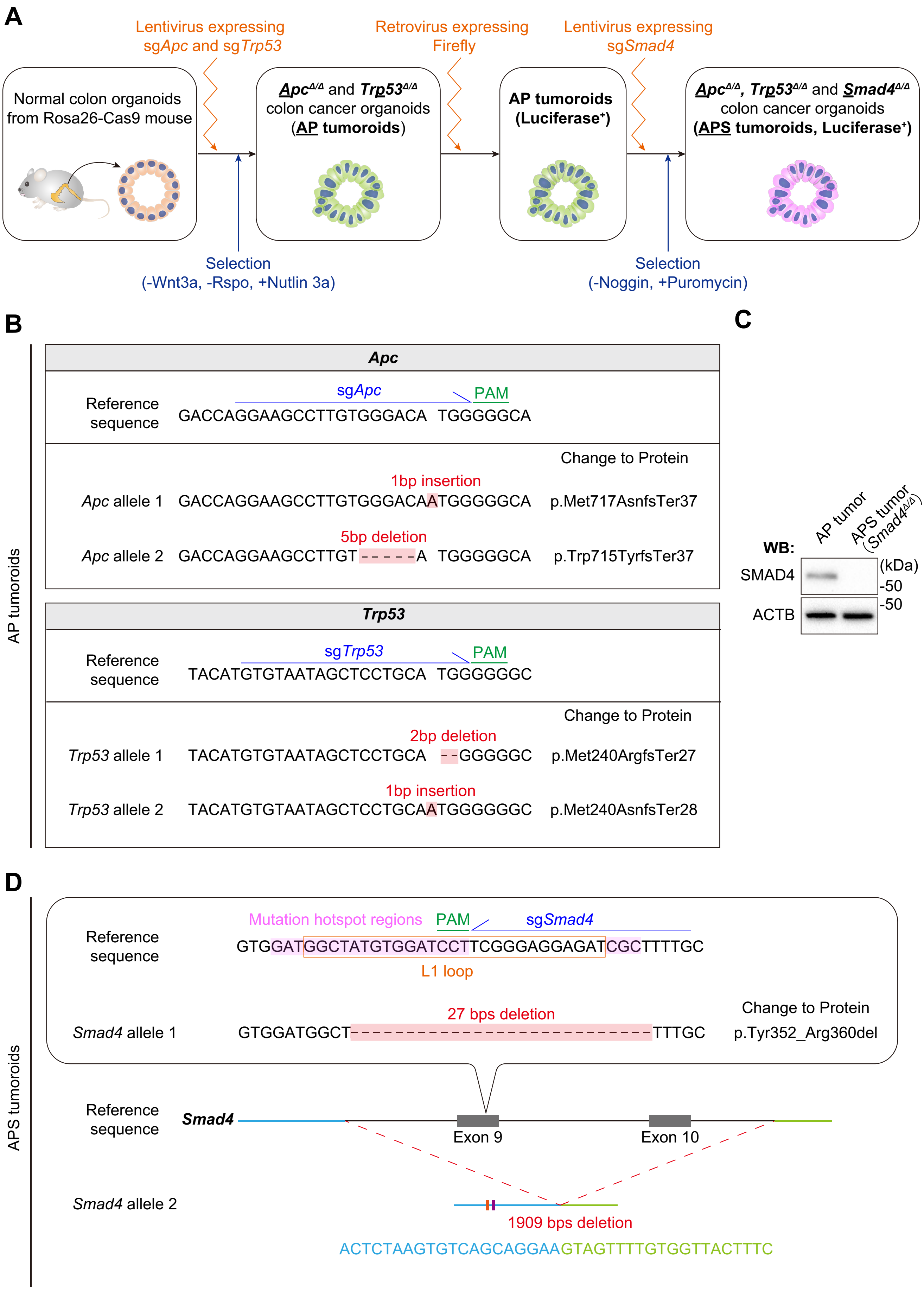
A
Lentivirus expressing sgApc and sgTrp53
Retrovirus expressing Firefly
Lentivirus expressing sgSmad4
Normal colon organoids from Rosa26-Cas9 mouse
Apc$^{\Delta/\Delta}$ and Trp53$^{\Delta/\Delta}$ colon cancer organoids (AP tumoroids)
AP tumoroids (Luciferase^{+})
Apc$^{\Delta/\Delta}$, Trp53$^{\Delta/\Delta}$ and Smad4$^{\Delta/\Delta}$ colon cancer organoids (APS tumoroids, Luciferase^{+})
Selection (-Wnt3a, -Rspo, +Nutlin 3a)
Selection (-Noggin, +Puromycin)
B
AP tumoroids
Apc
Reference sequence
sgApc
PAM
GACCAGGAAGCCTTGTGGGACA TGGGGGCA
Change to Protein
1bp insertion
Apc allele 1 GACCAGGAAGCCTTGTGGGACAATGGGGGCA p.Met717AsnfsTer37
5bp deletion
Apc allele 2 GACCAGGAAGCCTTGT-----A TGGGGGCA p.Trp715TyrfsTer37
Trp53
Reference sequence
sgTrp53
PAM
TACATGTGTAATAGCTCCTGCA TGGGGGGC
Change to Protein
2bp deletion
Trp53 allele 1 TACATGTGTAATAGCTCCTGCA --GGGGGC p.Met240ArgfsTer27
1bp insertion
Trp53 allele 2 TACATGTGTAATAGCTCCTGCAATGGGGGGC p.Met240AsnfsTer28
C
WB:
AP tumor
APS tumor (Smad4$^{\Delta/\Delta}$)
SMAD4
ACTB
(kDa)
-50
-50
D
APS tumoroids
Reference sequence
Mutation hotspot regions
PAM
sgSmad4
GTGGATGGCTATGTGGATCCTTCGGGAGGAGATCGCTTTTGC
L1 loop
27 bps deletion
Change to Protein
Smad4 allele 1 GTGGATGGCT---------------------------TTTGC p.Tyr352_Arg360del
Reference sequence
Smad4
Exon 9
Exon 10
Smad4 allele 2
1909 bps deletion
ACTCTAAGTGTCAGCAGGAAGTAGTTTTGTGGTTACTTTC

Supplementary Figure 16. Generation of *Apc* $^{\Delta/\Delta}$,*Trp53* $^{\Delta/\Delta}$ organoids (AP tumoroids) and *Smad4*-mutant AP tumoroids (APS tumoroids). (*A*) Schematic illustration of the CRISPR/Cas9–mediated sequential engineering of AP tumoroids and APS tumoroids from normal colon organoids isolated from a Rosa26-Cas9 mouse. Correctly targeted clones were enriched by selection medium as indicated. Monoclonal lines were handpicked after each transduction step. (*B*) *Apc* and *Trp53* DNA sequence verification of biallelic insertion/deletion mutations, which result in prematurely truncated proteins. (*C*) Decreased SMAD4 protein expression in APS tumoroids was validated by WB. (*D*) *Smad4* DNA sequence verification of biallelic mutations, resulting in disruption of the sequence of L1 loop protein, which is involved in the formation of SMAD protein complex formation.[32] sg*Smad4* was designed to target the genomic sequence corresponding to *SMAD4* mutation hotspot regions in human CRC,[32] which were mapped to the L1 loop and its adjacent region.[32] To simplify graphical presentation, the following mutations were not shown in the schematics in *D*: *Smad4* allele 1, c.1005A>G (silent mutation in exon 9) ; *Smad4* allele 2, c.953-831A>T (mutation in an intron; *orange bar*); and c.953-805_953-804insAAAAAATAAATAAAAAAAATAAAT (mutation in an intron; *Magenta bar*). Changes to proteins are reported according to the Human Genome Variation Society nomenclature. Reference sequences are as follows: *Apc*, NM_001360980.1; *Trp53*, NM_011640.3; *Smad4*, NM_008540.3. fs, frameshift mutation; PAM, protospacer adjacent motif; sg, single guide RNA; Ter, translation termination (stop) codon.

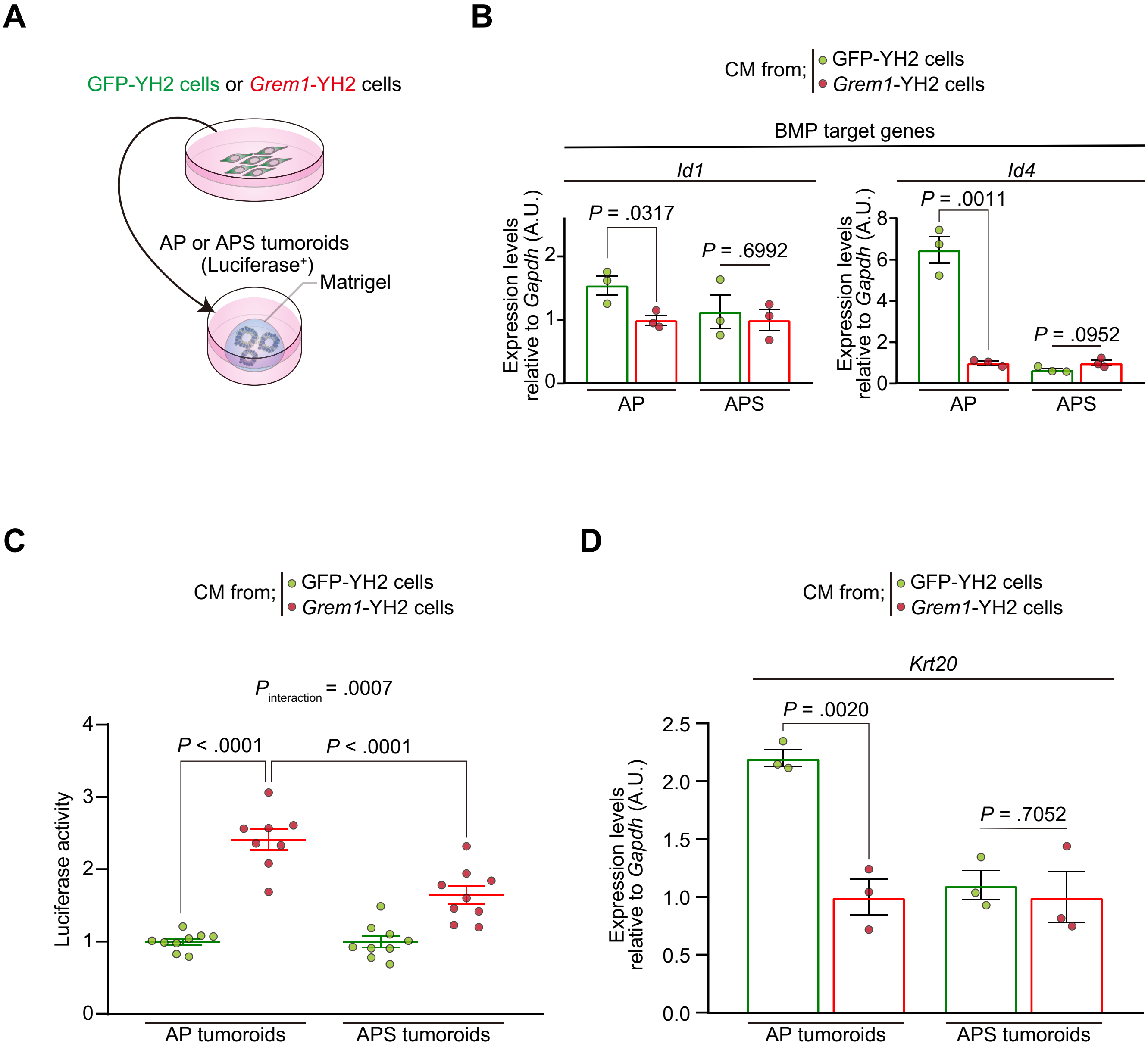

Supplementary Figure 17. CM transfer from *Grem1*-overexpressing YH2 cells decreases BMP signaling and accelerates organoid growth in AP tumoroids, but not in APS tumoroids. (*A*) Experimental schematic depicting CM transfer from *Grem1*-overexpressing YH2 cells or GFP-overexpressing YH2 cells to AP (*Apc*$^{\Delta/\Delta}$,*Trp53* $^{\Delta/\Delta}$) tumoroids or APS (*Apc*$^{\Delta/\Delta}$,*Trp53*$^{\Delta/\Delta}$,*Smad4*$^{\Delta/\Delta}$) tumoroids. (*B*) qRT-PCR for BMP target genes in AP and APS tumoroids (n = 3). (*C*) Luciferase signals from AP tumoroids and APS tumoroids (n $\geq$ 8). (*D*) qRT-PCR for *Krt20* in AP tumoroids and APS tumoroids (n = 3). Note that data normalization was performed within the AP and APS tumoroid groups separately (*B*–*D*). Statistical analyses were performed by using the 2-tailed unpaired Student *t* test (*B* and *D*) and 2-way ANOVA with Tukey post hoc multiple comparisons (C). Data are represented as mean ± SEM.

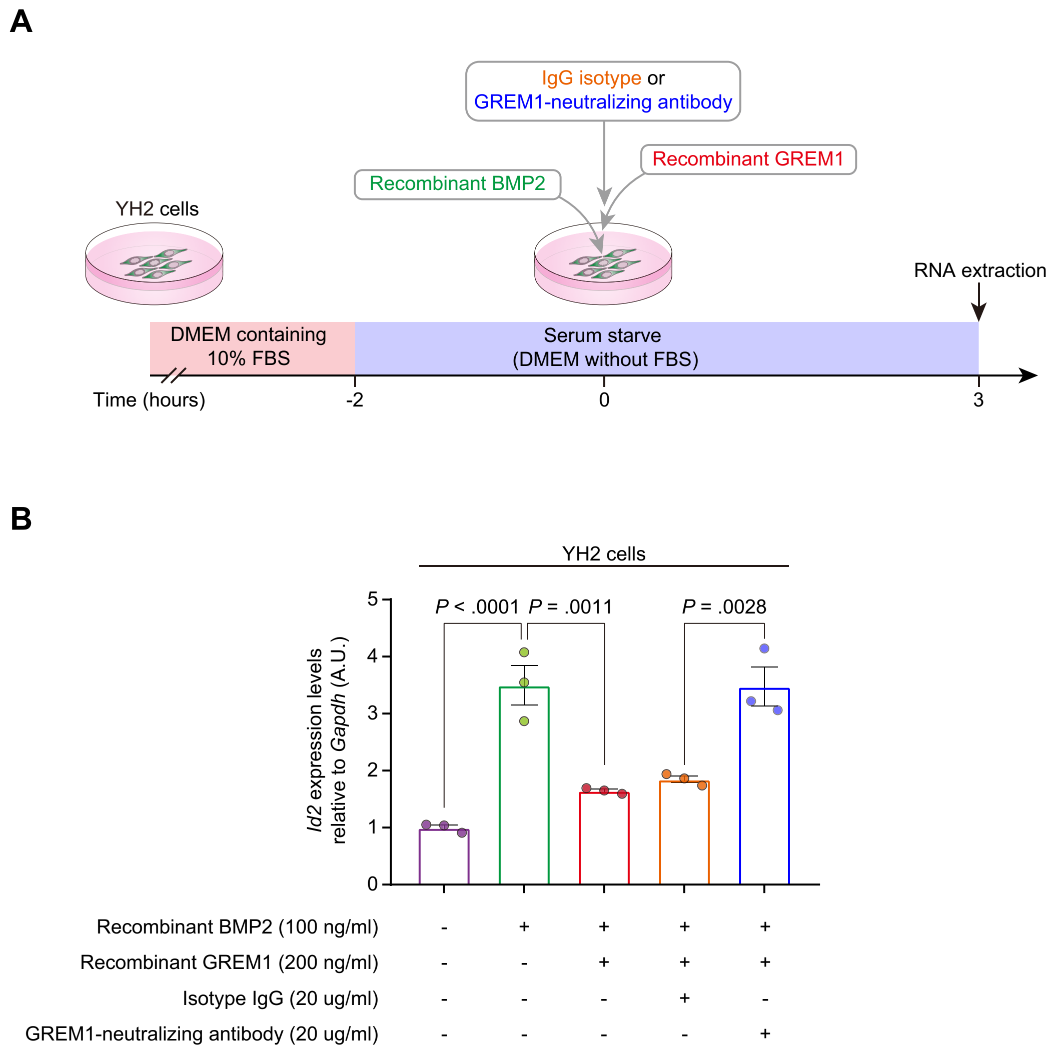

Supplementary Figure 18. Treatment with a GREM1-neutralizing antibody blocks GREM1-mediated inhibition of BMP2 signaling in vitro. (*A*, *B*) Validation of the GREM1-neutralizing antibody using YH2 cells. (*A*) Experimental schematic. YH2 cells were serum-starved for 2 hours before treating with the indicated proteins for 3 hours. (*B*) qRT-PCR of *Id2* in YH2 cells was performed to confirm the effect of the GREM1-neutralizing antibody in neutralizing recombinant GREM1 and restoring BMP signaling. n = 3 each. Data are represented as mean ± SEM. Statistical analyses were performed by using 1-way ANOVA with Tukey post hoc multiple comparisons (*B*).

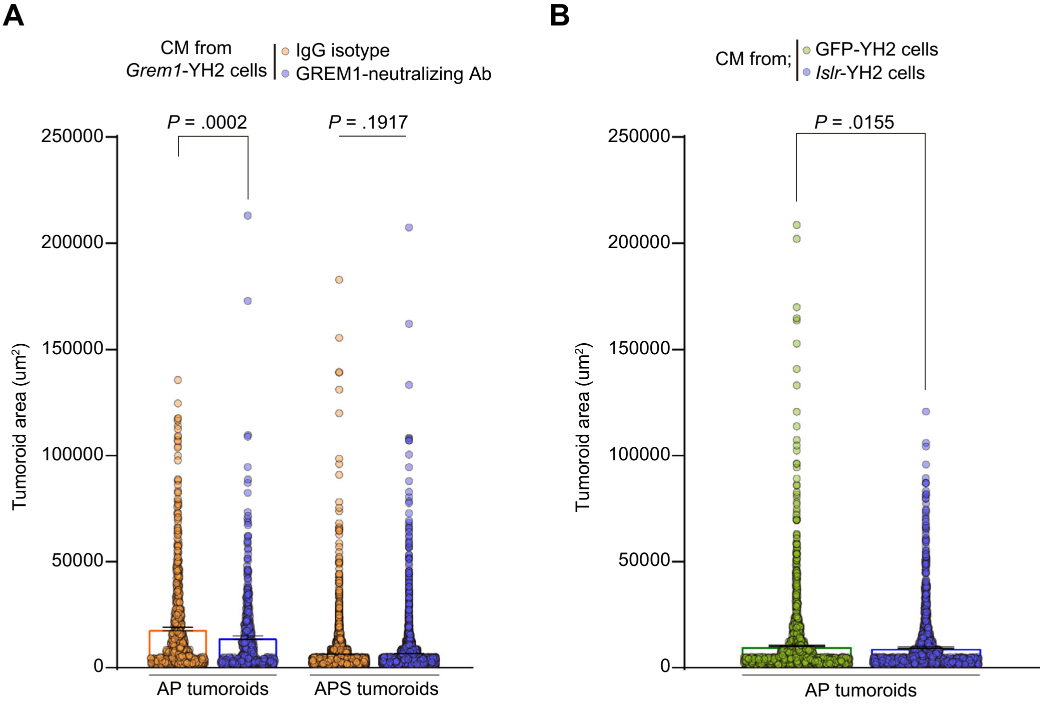

Supplementary Figure 19. Treatment with a GREM1-neutralizing antibody or CM from *Islr*-overexpressing intestinal fibroblasts attenuates tumoroid growth as assessed by tumoroid area. (*A*) Treatment with a GREM1-neutralizing antibody reduces AP tumoroid areas. n = 790, 522, 3571, and 3494 tumoroids (*left to right*) from 6 (AP tumoroids) and 9 independent replicates (APS tumoroids). Note that AP tumoroid and APS tumoroids were evaluated 8 and 12 days after being plated. In this experimental condition, APS tumoroids generated smaller tumoroid areas than AP tumoroids when treated with an IgG isotype ($P < .0001$, Mann-Whitney *U* test). (*B*) CM transfer from *Islr*-overexpressing YH2 cells decreases AP tumoroid areas. n = 1873 (GFP-YH2 cells) and 1713 tumoroids (*Islr*-YH2 cells) from 9 independent replicates. The AP tumoroids were evaluated 7 days after being plated. Tumoroid areas were assessed by Image J. Mean ± SEM, Mann-Whitney *U* test (*A* and *B*). Ab, antibody.

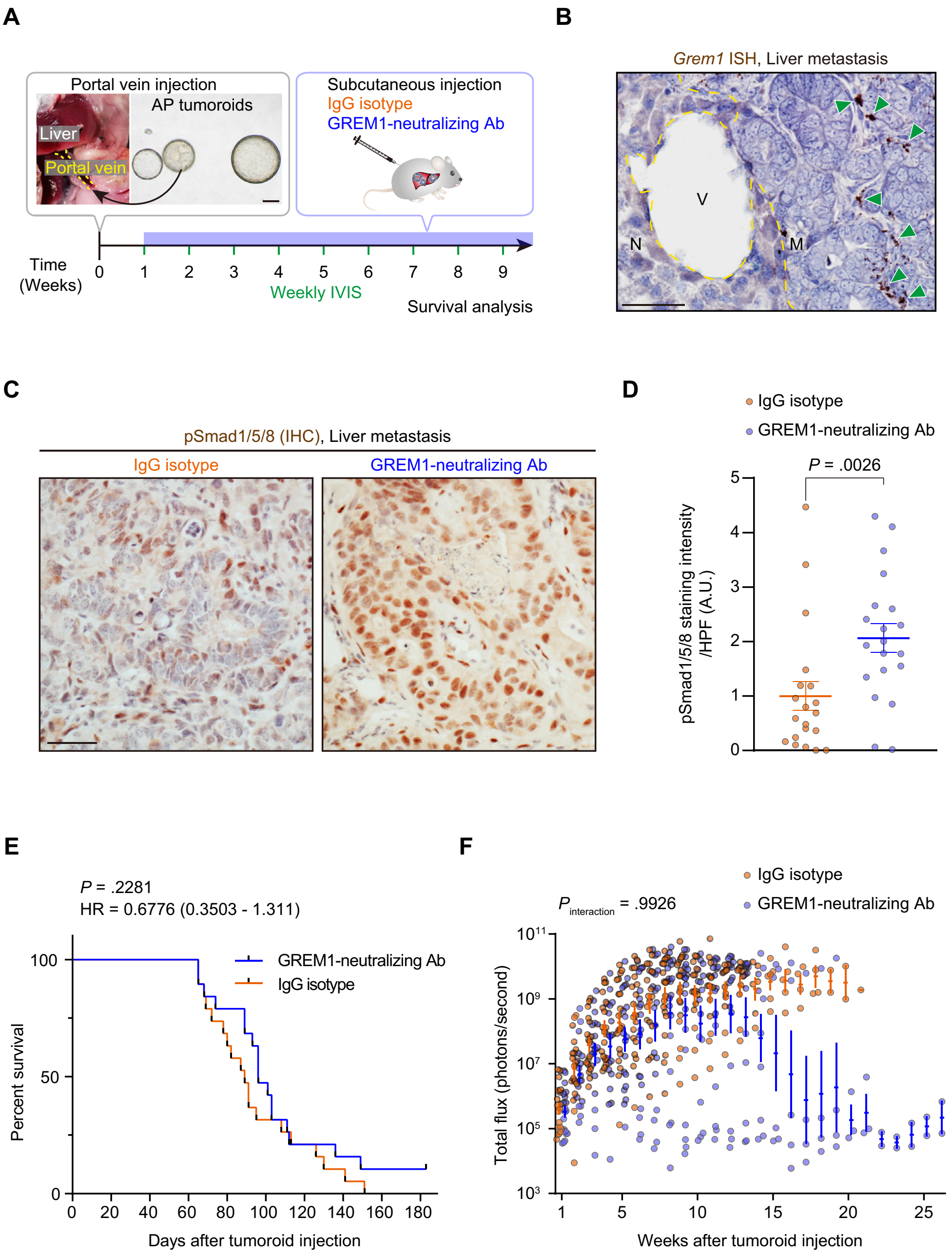

Supplementary Figure 20. A GREM1-neutralizing antibody–treated group showed enhanced BMP signaling and a trend toward prolonged survival in a mouse model of CRC liver metastasis. (*A*) Experimental scheme for portal vein injection of AP tumoroids and subsequent GREM1-neutralizing antibody administration. The GREM1-neutralizing antibody was subcutaneously administered 1 week after portal vein injection of AP tumoroids until mice reached humane endpoints. Yellow dotted lines outline the portal vein. Scale bar, 200 μm. (*B*) ISH for *Grem1* in mouse CRC liver metastasis. *Grem1* was expressed by the fibroblastic cells in the liver metastasis mesenchyme (M) but not in the normal liver (N). Yellow dotted lines demarcate the normal liver (N) and CRC liver metastasis (M). Green arrowheads indicate *Grem1* expression, and V indicates a blood vessel. n = 3 mice. Scale bar, 50 μm. (*C*, *D*) IHC for pSmad1/5/8 in the liver metastases developed in IgG isotype-treated or GREM1-neutralizing antibody–treated mice. (*C*) Representative pictures. Scale bar, 50 μm. (*D*) Quantification of 3,3′-DAB intensity by ImageJ; 4 HPFs/mouse, 5 mice each. (*E*) Kaplan-Meier survival curves. n = 19 (IgG isotype) and 19 mice (GREM1-neutralizing antibody). Hazard ratio (HR) and 95% confidence interval of the HR are shown (log-rank method). (*F*) The growth kinetics of CRC hepatic metastases were assessed during the survival analysis in *E*. AP tumoroid–derived luciferase signals were evaluated by using an IVIS. Mean ± SEM. Mann-Whitney *U* test (*D*), log-rank test (*E*), and 2-way repeated-measures ANOVA (*F*). In *F*, to exclude the effect of loss of samples during the experiment, statistical analysis was performed by using IVIS signal values week before 10, when the first mouse reached a humane endpoint. Note that the same pictures were used in Figure 6*A* and Supplementary Figure 20*A* to show the procedure of portal vein injection.

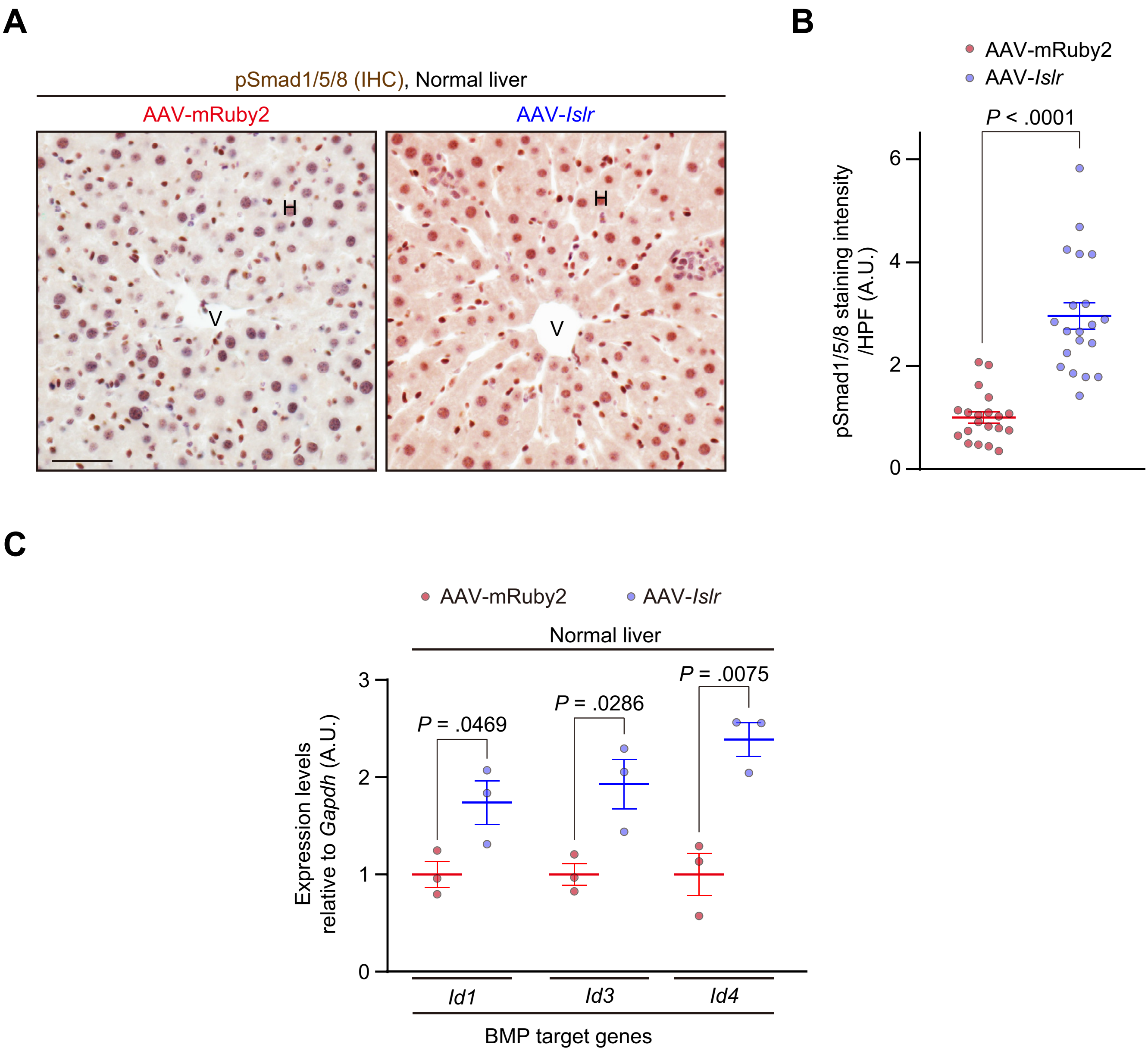

Supplementary Figure 21. AAV-*Islr* treatment augments BMP signaling in the normal mouse liver. (*A*, *B*) IHC for pSmad1/5/8 in the normal liver from AAV-*Islr*–treated or AAV-mRuby2–treated mice. (*A*) Representative pictures. Scale bar, 50 μm. H, hepatocytes; V, central vein. (*B*) Quantification of 3,3′-DAB intensity by ImageJ; 5 HPFs/mouse, 4 mice each. (*C*) qRT-PCR for BMP target genes using normal liver tissue lysates; n = 3 mice each. Mice were harvested 5–6 weeks after tail vein injection (3–4 weeks after tumor injection) (*A*–*C*). Mean ± SEM. Mann-Whitney *U* test (*B*) and 2-tailed unpaired Student *t* test (*C*).

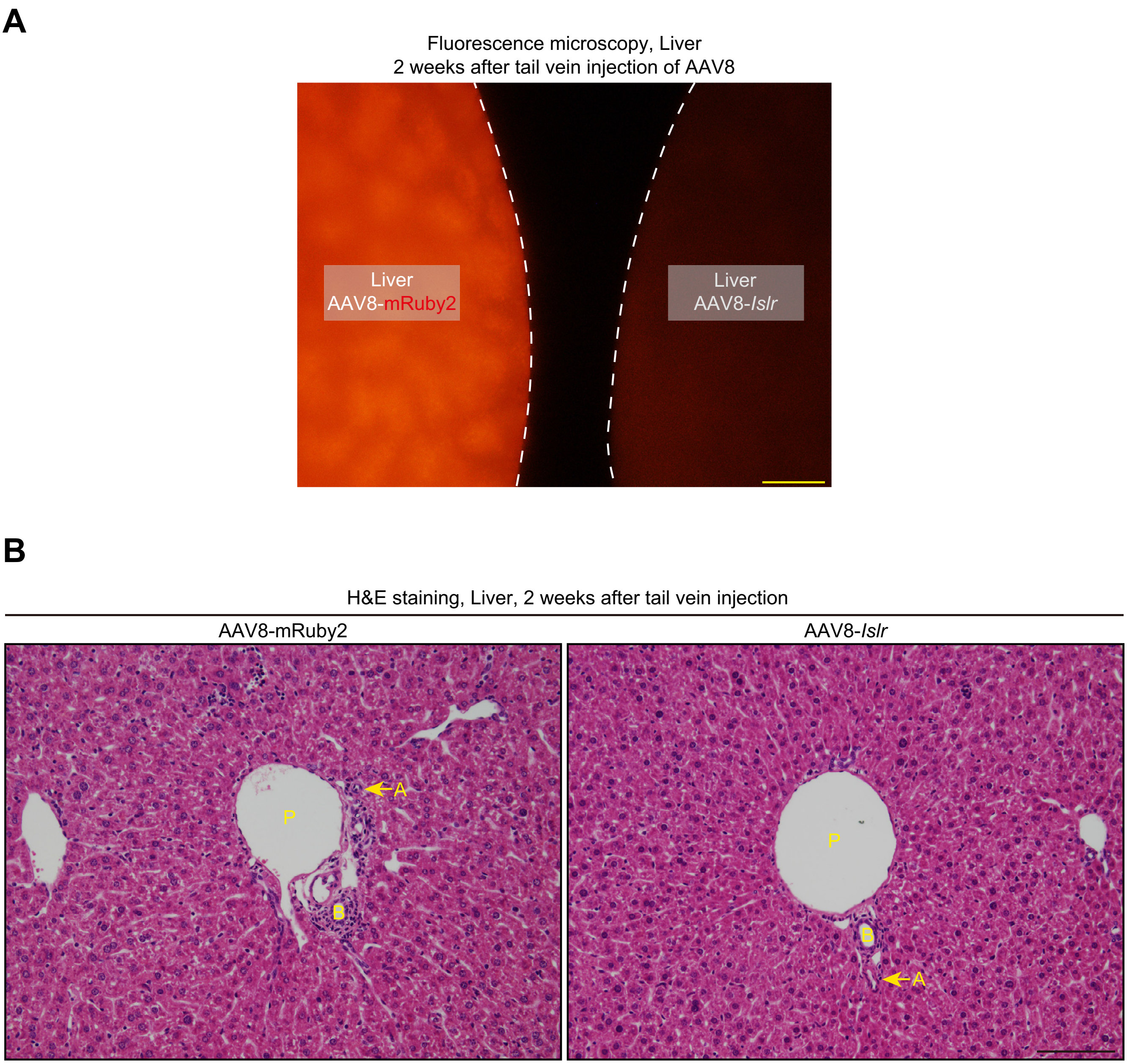

Supplementary Figure 22. No liver injury was apparent in mice administered AAV8-*Islr* via histologic analysis. (*A*) Fluorescent microscopy image showing expression of a red fluorescent protein (mRuby2) in the liver of mice harvested 2 weeks after tail vein injection of AAV8-mRuby2 and AAV8-*Islr*. White dotted lines indicate the edges of the livers. Yellow scale bar, 500 μm. (*B*) H&E staining of the liver section from mice harvested 2 weeks after tail vein injection of AAV8-mRuby2 and AAV8-*Islr*; n = 3 mice each. A, hepatic artery (arrows); B, bile duct; P, portal vein. Scale bar, 100 μm.

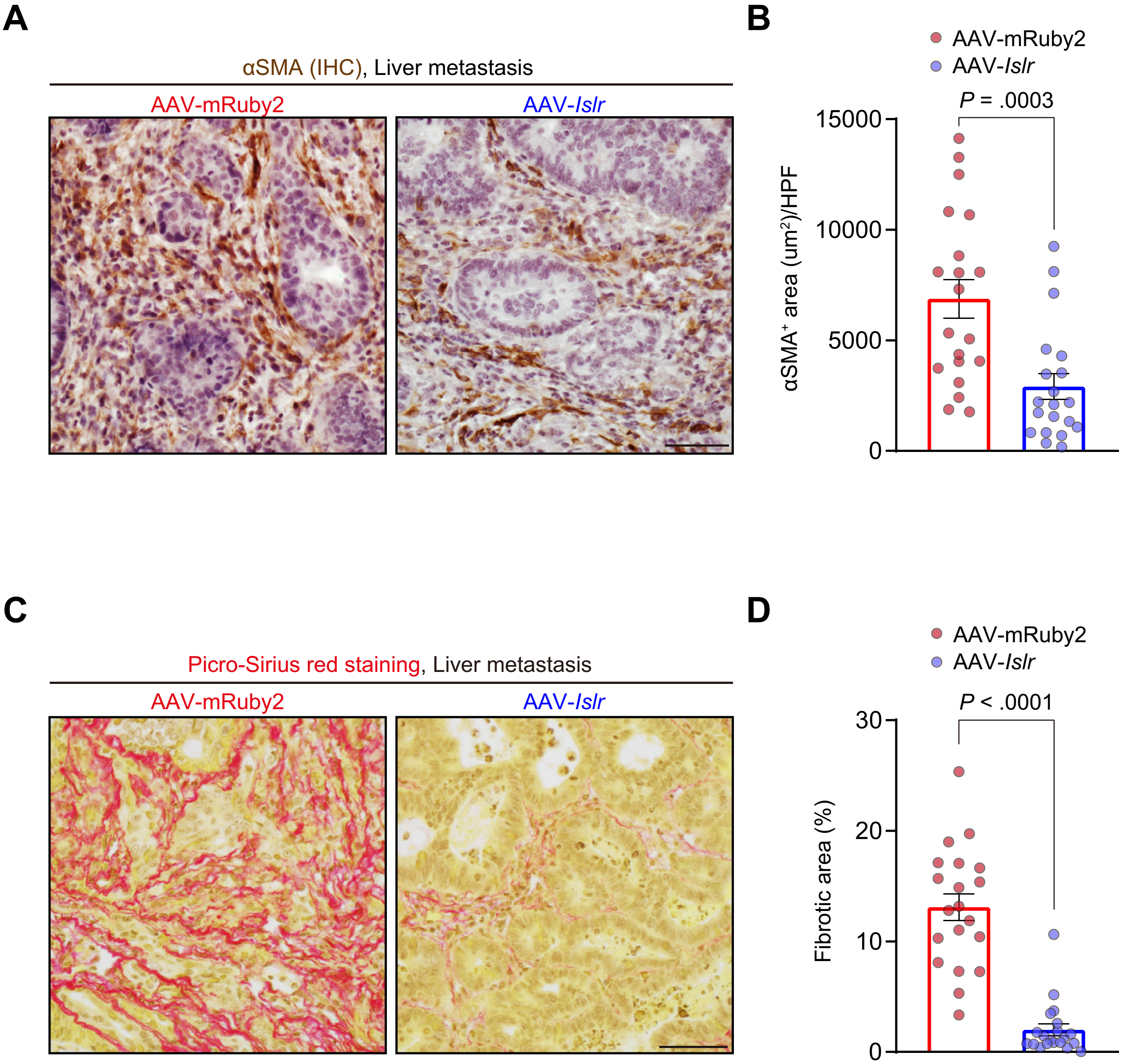

Supplementary Figure 23. Mice administered AAV8-*Islr* show reduced fibrosis in CRC liver metastases. (*A*, *B*) IHC for αSMA using CRC liver metastases in mice administered AAV8-*Islr* or AAV8-mRuby2. (*A*) Representative pictures. (*B*) Quantification of 3,3′-DAB^{+} areas by ImageJ; 5 HPFs/mouse, 4 mice each. (*C*, *D*) Picro-Sirius red staining for collagen using the CRC liver metastasis sections. (*C*) Representative pictures. (*D*) Quantification of Picro Sirius red^{+} areas by ImageJ; 5 HPFs/mouse, 4 mice each. Mean $\pm$ SEM. Mann-Whitney *U* test (*B* and *D*). Scale bars, 50 μm.

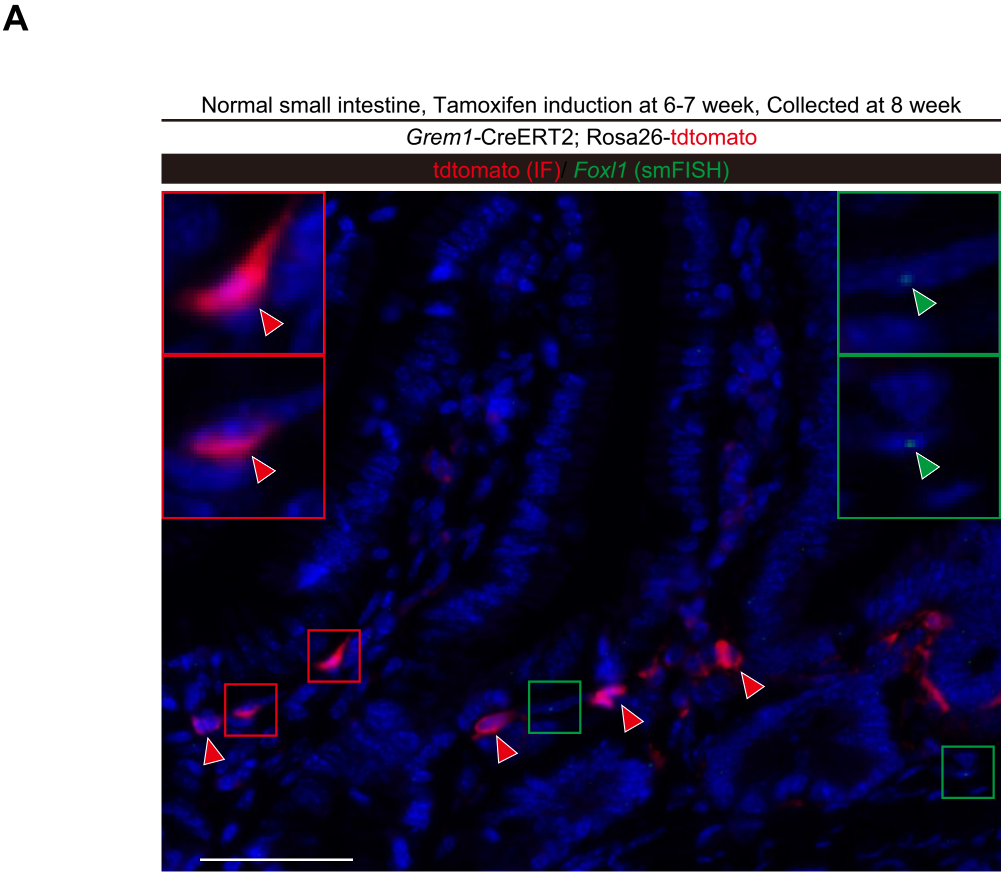

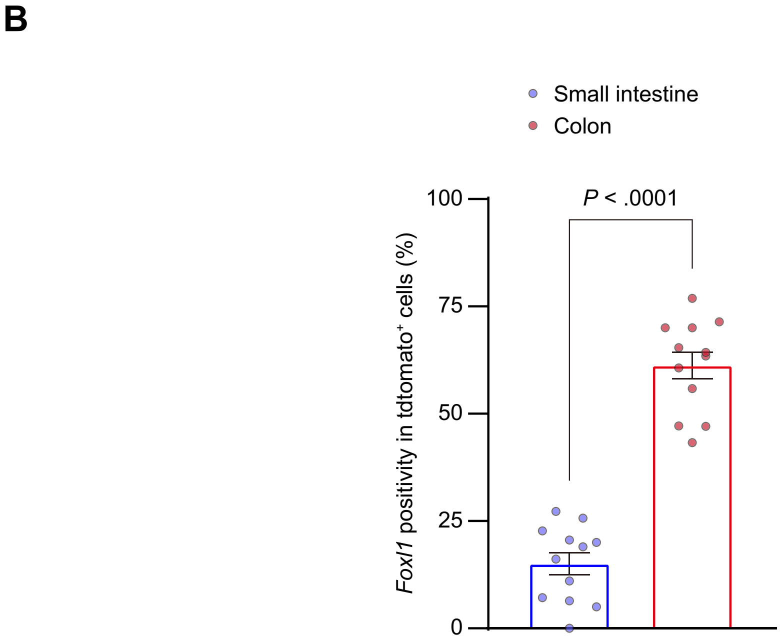

Supplementary Figure 24. *Grem1*$^+$ cells in the small intestine show lower *Foxl1* positivity than those in the colon. (*A*, *B*) smFISH for *Foxl1* and IF for tdtomato in the small intestine from *Grem1*-CreERT2;Rosa26-tdtomato mice. (*A*) Representative picture. Red and green arrowheads denote *Grem1*$^+$*Foxl1*$^-$ cells and *Grem1*$^-$*Foxl1*$^+$ cells, respectively. The boxed areas are magnified in the insets. (*B*) *Foxl1* positivity in the *Grem1*$^+$ cells in the small intestine (*blue*). For statistical comparison, the result with the colon from Figure 3*D* is shown on the right (*red*). Four HPFs (400×)/mouse, 3 mice each. Two-tailed unpaired Student *t* test. Scale bars, 50 μm.
